# Ferrocene donor linked to pyridine/pyridinium acceptor *via* a systematically enlarged π-linker†

**DOI:** 10.1039/d1ra08186a

**Published:** 2021-12-03

**Authors:** Jiří Kulhánek, Milan Klikar, Oldřich Pytela, Zdenka Růžičková, Filip Bureš

**Affiliations:** Institute of Organic Chemistry and Technology, Faculty of Chemical Technology, University of Pardubice Studentská 573 Pardubice 53210 Czech Republic filip.bures@upce.cz; Department of General and Inorganic Chemistry, Faculty of Chemical Technology, University of Pardubice Studentská 573 Pardubice 53210 Czech Republic

## Abstract

Nine chromophores with ferrocene donor and pyridine/pyridinium acceptors have been prepared and further investigated. The performed X-ray analysis showed partially polarized and geometrically oblate pyridine unit. An extension of the π-system and *N*-quaternization were revealed as suitable tools for exclusive manipulation of the LUMO with the almost steady HOMO. Whereas the electrochemical HOMO–LUMO gap can be tuned from 3.01 to 1.49 eV, the high- and low-energy absorption bands were found within the range of 280–402/456–547 nm. The pyridinium chromophores showed distinct negative solvatochromism. A thorough DFT analysis has been performed; it turned out that ferrocene donor is capable of two principal D–A interactions, whose employment depends on the appended electron-withdrawing moiety.

## Abstract 2

Nine chromophores with ferrocene donor and pyridine/pyridinium acceptors have been prepared and further investigated.

## Graphical abstract

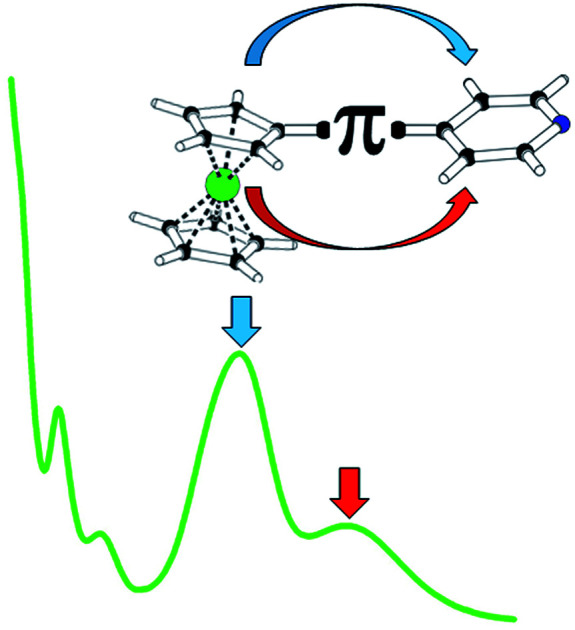

## Introduction

Due to their D–π–A arrangement (D = donor, π = conjugated system and A = acceptor), organic push–pull molecules represent a unique class of organic π-conjugated systems.^1^ A direct electron communication between the D and A parts called intramolecular charge-transfer (ICT) imparts push–pull molecules distinct properties such as dipolar structure, optical properties and colour, supramolecular arrangement, conductivity, solubility *etc.* Moreover, the ICT in organic D–π–A molecules is tuneable within a broad range, which allows tailoring their properties towards particular applications across organic electronics and photonics. Push–pull molecules are commonly found as active substances of organic light-emitting diodes (OLED),^2^ organic field-effect transistors (OFET),^3^ organic photovoltaic cells (OPVC)^4^ and dye-sensitized solar cells (DSSC).^5^ Due to their dipolar and polarizable structure, organic push–pull chromophores found also numerous applications across nonlinear optics (NLO).^1,6^

In contrast to inorganic systems, organic D–π–A molecules with well-defined structure are often easy to synthesize and processed/engineered. In principle, their property tuning involves modification of D, π and A parts as well as their overall arrangement.^7^ Whereas electron releasing D part is commonly introduced by appending groups with positive electronic effects (+I and +M; *e.g.* dialkylamino/alkoxy groups), electron withdrawing groups possess opposite effects (*e.g.* cyano/nitro groups). In addition to these common donors and acceptors, complex heterocyclic units such as proaromatic pyranylidene, thienothiophenes, carbazole and dicyanovinyl, dicyanoimidazole, tricyanofuran, indandione, (thio)barbituric acid, ThDione, *etc.* were also widely employed in push–pull molecules.^7,8^ The π-system involves multiple bonds, aromatic and heterocyclic units and their combinations.

Metallocenes, ferrocene (Fc) in particular, are electron-rich moieties employed as donors in push–pull chromophores.^9^ The electroactive ferrocene incorporated into a push–pull molecule brings defined redox process,^10^ chromophore nonplanarity^11^ and enhanced thermal stability.^12^ On the contrary, six-membered pyridine (Py) belongs to electron poor heterocycles, which may be utilized as an electron acceptor.^1,13^ A mutual conjugated interconnection of Fc and Py moieties renders a push–pull system. Fc–π–Py molecules were widely utilized as tailored ligands chelating (transition) metals.^14^ The resulting complexes often showed cytotoxicity or distinct electrochemical sensing towards metal cations.

Various dyes, mostly in push–pull arrangement, were constructed from the Fc and Py moieties.^15^ Many of them showed very interesting and pronounced NLO properties.^16^ Very nice example of a functionalized Fc-derivative with two linked Py-pendants, featuring photoelectric properties adjustable by pH, has recently been reported by Shi *et al.*^17^ In 2013, ferrocene electron donor has been utilized for the construction of push–pull dyes also in our laboratory.^18^

**Fig. 1:**
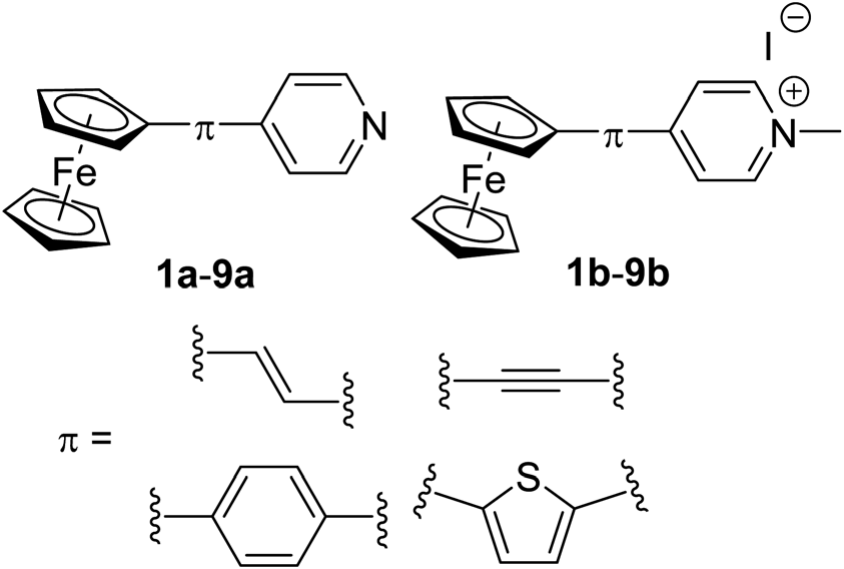
General structure of investigated Fc–π–Py and Fc–π–Py^+^ push–pull chromophores.

In order to extend these studies and in view of the pyridine facile quaternization capability,^15*a*,19^ we report herein Fc-derived push–pull molecules with pyridyl (Py) and pyridinium (Py^+^) acceptors (Fig. 1). Besides employing two related Py/Py^+^ acceptors, further property tuning has been achieved by altering the π-conjugated pathway.

## Results and discussion

### Synthesis

**Scheme 1:**
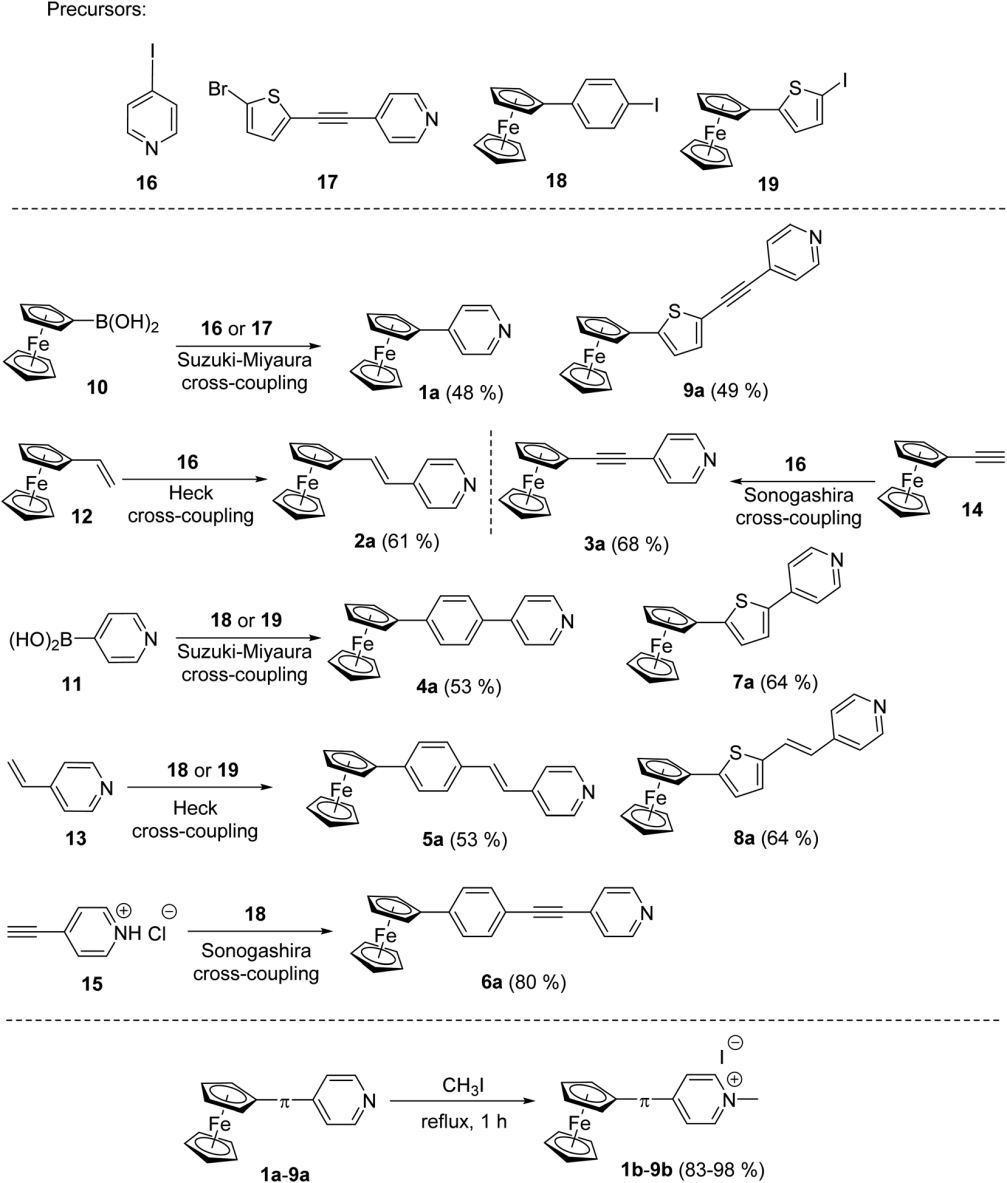
Synthetic strategy towards Fc–π–Py push–pull chromophores 1a–9a with systematically enlarged π-system and their subsequent *N*-quaternization to 1b–9b.

The synthesis of pyridine (a) and pyridinium (b) chromophores 1–9 involved preparation of Fc–π–Py compounds and their subsequent quaternization using iodomethane. The synthesis of compounds 1a–9a utilized three cross-coupling reactions as outlined in Scheme 1. Compounds 1a and 9a were prepared using ferroceneboronic acid and commercially available 4-iodopyridine 16 and intermediate 17 (see the ESI† for its synthesis). Similar Suzuki–Miyaura reaction^14*c*^ between commercial pyridin-4-ylboronic acid 11 and precursors 18 (see the ESI†) or 19 (ref. 18*b*) has also been employed for the construction of compounds 4a and 7a. Heck olefination^18*a*^ between vinylferrocene 12 or 4-vinylpyridine 13 with 4-iodopyridine 16 and precursors 18 and 19 afforded target Fc–π–Py chromophores 2a (61%), 5a (53%) and 8a (64%). A modified procedure for Sonogashira reaction^18*a*^ has been employed for preparation of compounds 3a and 6a. Commercially available ethynylferrocene 14 and precursor 16 afforded 3a in 68% yield, while chromophore 6a was prepared by reacting 4-ethynylpyridine hydrochloride 15 with 18. The pyridinium salts 1b–9b were gained by reacting 1a–9a with boiling iodomethane in the yield ranging between 83 and 98% (Scheme 1).

### X-ray analysis

**Fig. 2:**
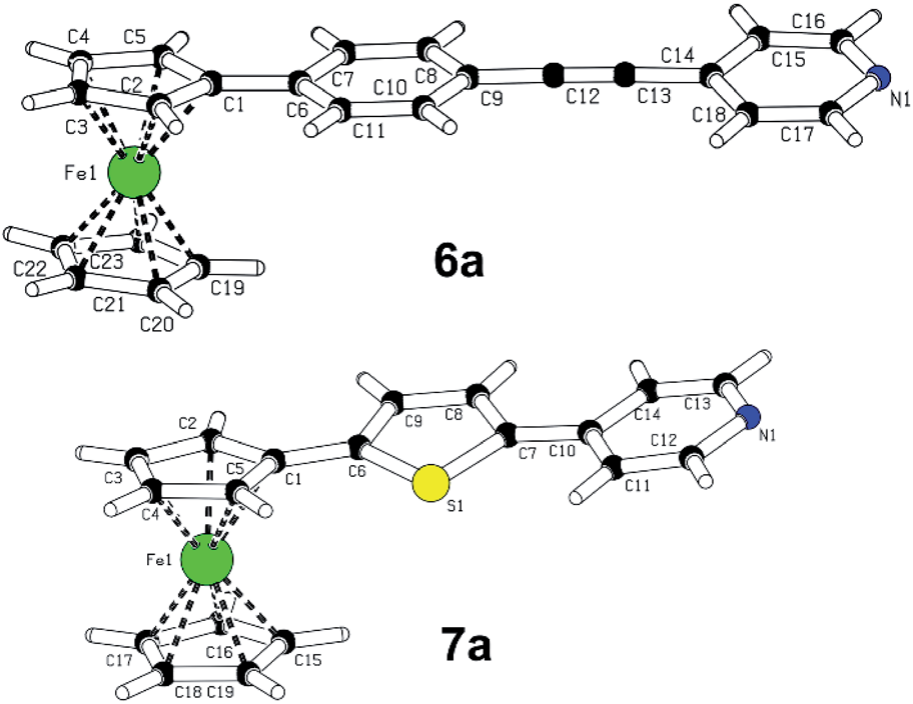
X-ray molecular representation of chromophore 6a (CCDC 2110543) and 7a (CCDC 2110542).

A slow evaporation of dichloromethane solutions of compounds 6a and 7a provided mono-crystals suitable for X-ray analysis, see the ESI† for complete details. The plots shown in Fig. 2 confirm their molecular structure and the solid-state arrangement. Their structures are composed of almost coplanar arrangement of the ferrocene Cp ring and the appended π-system with diminished interplanar angles ranging from 0.5 to 12°. The extent of the ICT in 6a and 7a may be assessed by calculating bond-length alternation of the particular π-system. Using Bird index (*I*_6_/*I*_5_),^20^ the indexes *I*_6_/*I*_5_ of unsubstituted benzene/thiophene are 100 and 66, respectively. The experimental values obtained for 1,4-phenylene (92.8) and 2,5-thienylene (60.5) moieties in 6a and 7a imply that both molecules are polarized and partially adopt quinoid character. Moreover, thiophene polarization in 6a is not far from that observed for T-shaped chromophores (*I*_5_ ∼58) featuring very efficient ICT.^21^ On the contrary, the *I*_6_ values of the terminal pyridine rings (94.3 and 91.6) are higher than expected for unsubstituted pyridine (*I*_6_ = 85.7). However, when employing harmonic oscillator model of aromaticity (HOMA),^22^ the obtained values 6a and 7a are 0.875 and 0.897, while unsubstituted pyridine possesses the HOMA within the range of 0.944–1 (based on the source data). Hence, the pyridine rings in 6a/7a are considered as partially polarized and geometrically oblate with the shortest C–N bonds (1.33–1.39 Å). The supramolecular arrangement of 6a revealed two-component inversion twin.

### Electrochemistry

The electrochemical characteristics of pyridine derivatives 1a–9a as well as pyridinium salts 1b–9b were investigated in acetonitrile containing 0.1 M Bu_4_NPF_6_ in a three-electrode cell by cyclic voltammetry (CV). The acquired data are summarized in Table 1, see the ESI† for a complete list of CV diagrams (Fig. S7–12†).

**Table 1:** Experimentally obtained electrochemical and optical parameters of chromophores 1a–9a and 1b–9b

Comp.	*E*_pa(ox1)_ a [V]	*E*_pc(ox1)_ a [V]	*E*_1/2(ox1)_ b [V]	*E*_pc(red1)_ a [V]	*E*_pa(red1)_ a [V]	*E*_1/2(red1)_ b [V]	Δ*E*^d^ [eV]	*E*_HOMO_ e [eV]	*E*_LUMO_ e [eV]	*λ* ^HE^_max_ [nm (eV)]/*ε* (×10^3^)^f^ [M^−1^ cm^−1^]	*λ* ^LE^_max_ [nm (eV)]/*ε* (×10^3^)^f^ [M^−1^ cm^−1^]
1a	0.45	0.38	0.42	−2.56	c	—	3.01	−4.92	−1.91	280 (4.43)/10.5	456 (2.72)/0.6
2a	0.37	0.30	0.34	−2.22	c	—	2.59	−4.84	−2.25	312 (3.97)/22.8	461 (2.69)/1.9
3a	0.5	0.42	0.46	−2.28	c	—	2.78	−4.97	−2.19	303 (4.09)/15.5	457 (2.71)/1.1
4a	0.38	0.32	0.35	−2.32	c	—	2.70	−4.85	−2.15	300 (4.13)/23.3	460 (2.70)/1.3
5a	0.36	0.29	0.33	−2.03	c	—	2.39	−4.83	−2.44	331 (3.75)/34.5	457 (2.71)/2.5
6a	0.39	0.32	0.36	−2.09	c	—	2.48	−4.86	−2.38	317 (3.91)/32.7	460 (2.70)/2.0
7a	0.41	0.33	0.37	−2.19	−2.04	−2.12	2.60	−4.88	−2.28	336 (3.69)/19.3	460 (2.70)/2.0
8a	0.39	0.31	0.35	−1.97	−1.86	−1.92	2.36	−4.86	−2.50	366 (3.39)/29.2	462 (2.68)/4.3
9a	0.43	0.35	0.39	−2.06	−1.91	−1.99	2.49	−4.90	−2.41	345 (3.59)/25.9	464 (2.67)/3.0
1b	0.63	0.55	0.59	−1.46	−1.37	−1.415	2.09	−5.10	−3.01	313 (3.96)/15.9	525 (2.36)/2.7
2b	0.47	0.4	0.435	−1.24	c	—	1.71	−4.94	−3.23	361 (3.43)/25.1	547 (2.27)/6.3
3b	0.58	0.5	0.54	−1.15	c	—	1.73	−5.05	−3.32	343 (3.62)/20.1	531 (2.34)/5.1
4b	0.42	0.35	0.385	−1.32	−1.24	−1.28	1.74	−4.89	−3.15	344 (3.60)/22.2	501 (2.48)/4.1
5b	0.38	0.31	0.345	−1.12	c	—	1.5	−4.85	−3.35	378 (3.28)/33.9	508 (2.44)/7.2
6b	0.41	0.35	0.38	−1.08	c	—	1.49	−4.88	−3.39	362 (3.43)/29.7	504 (2.46)/5.8
7b	0.48	0.41	0.445	−1.26	−1.18	−1.22	1.74	−4.95	−3.21	390 (3.18)/22.6	537 (2.31)/6.2
8b	0.43	0.36	0.395	−1.08	c	—	1.51	−4.90	−3.39	426 (2.91)/32.3	532 (2.33)/11.6
9b	0.45	0.39	0.42	−1.05	c	—	1.5	−4.92	−3.42	402 (3.08)/24.7	523 (2.37)/8.0

a *E*_pa/c(ox1)_ and *E*_pa/c(red1)_ are anodic or cathodic peak potentials of the first oxidation and reduction, respectively, measured by CV at scan rate 100 mV × s^−1^; all potentials are given *vs.* SSCE (ACN).

b *E*_1/2(ox1)_ and *E*_1/2(red1)_ are half-wave potentials of the first oxidation and reduction, respectively, for reversible or quasi-reversible processes; *E*_1/2(ox1/red1)_ ≈ (*E*_pa(ox1/red1)_ + *E*_pc(ox1/red1)_)/2.

c Irreversible first reductions.

d Δ*E* = *E*_pa(ox1)_ − *E*_pc(red1)_.

e −*E*_HOMO/LUMO_ = (*E*_pa(ox1)_ + 0.036) or (*E*_pc(red1)_ + 0.036) + 4.429 (*vs.* SCE).^24^ The increment of +0.036 V corresponds to the difference between SCE (0.241 *vs.* SHE) and SSCE (0.205 *vs.* SHE).^25^

f Measured in ACN.

**Fig. 3:**
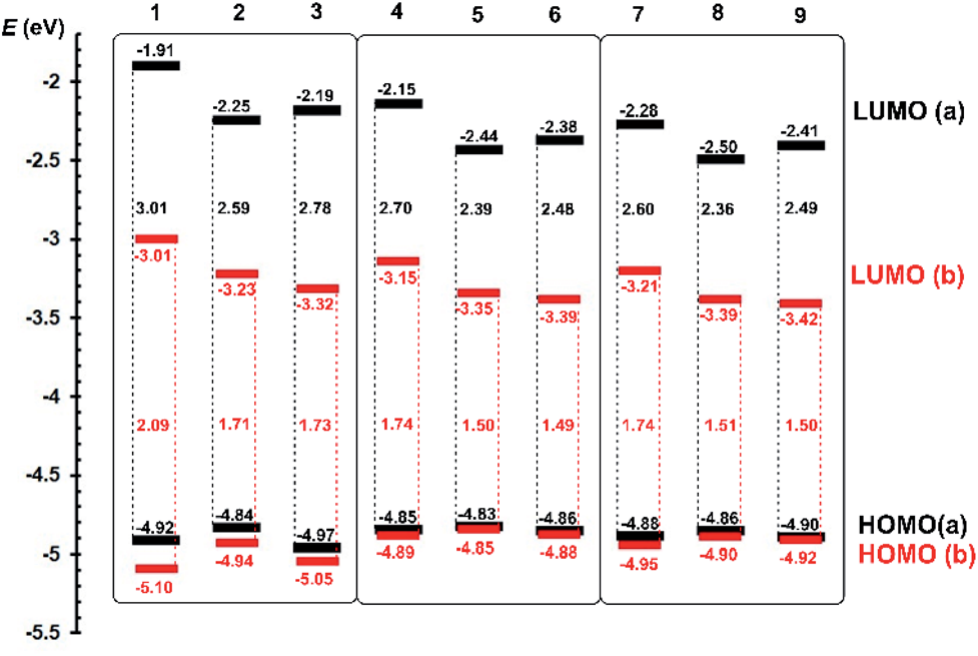
Energy level diagram of electrochemically determined HOMO and LUMO levels for Fc–π–Py (black) and Fc–π–Py^+^ (red) chromophores 1a–9a and 1b–9b.

Push–pull character of Fc–π–Py chromophores 1a–9a implies that the first oxidation most likely takes place on the ferrocene donor, while the first reduction involves the pyridine acceptor and adjacent π-linker. Due to ferrocene presence, the first oxidation was always determined as reversible process with peak-to-peak separation within the 60–80 mV implying one-electron transfer. On the other hand, the first reductions were recorded as irreversible or quasi-reversible processes followed by subsequent reductions with more negative potentials. As compared to *i*_pa(ox1)_ values, the first reductions possess two/three times higher cathodic peak current *i*_pc(red1)_, which corresponds to the two-electron reduction; three electron reductions were recorded for acetylene derivatives 3a, 6a and 9a. A precedent reduction process, seen as a shoulder accompanying the anodic peak of the ferrocene oxidation, has been observed for compounds bearing double/triple bonds. The peak potentials of the first oxidation/reduction *E*_p(ox1/red1)_ were recalculated to energies of the HOMO and LUMO (*E*_HOMO/LUMO_) (Table 1). These were further visualized in the energy level diagram shown in Fig. 3.

When going from 1a to 9a, the HOMO remained almost unaltered and principal changes are seen in the LUMO level. With the same donor and acceptor, these changes reflect composition of the π-linker. In chromophore threesomes 1a–3a, 4a–6a and 7a–9a, the lowest LUMO (and also the HOMO–LUMO gap Δ*E*) was recorded for compounds 2a, 5a and 8a bearing olefinic subunit. This is mostly due to its ICT transparency and planarization effect.^1^ When going from 4a–6a to 7a–9a, the latter showed narrowed Δ*E*, which obeys our comparison of 1,4-phenylene and 2,5-thienylene moieties embedded in push–pull molecules.^23^ Hence, by gradually extending the π-system (1a → 8a), the HOMO–LUMO gap may be tuned from 3.01 to 2.36 eV.

Electrochemical investigation of Fc–π–Py^+^ chromophores 1b–9b revealed additional I^−^ → I_3_^−^ oxidation located at around +400 mV appearing as a single wave (1b–3b and 7b–9b) or shoulder accompanying Fc redox process (4b–6b). In order to properly distinguish both redox processes, a joint CV diagram of representative compound 1b with its analogue quaternized by dimethylsulfate (containing electrochemically inactive MeOSO_3_^−^ anion instead of I^−^) along with the electrochemical oxidation of I^−^ (KI) is provided in the ESI (Fig. S13†). As can be seen from the energy level diagram in Fig. 3, the HOMO levels of pyridiniums 1b–9b remained almost unaltered as compared to the original pyridines 1a–9a. *N*-Quaternization significantly affected the LUMO levels that generally dropped by ∼1 eV.

Moreover, a combination of the pyridinium acceptor along with electronegative acetylenic unit (as in 6b and 9b) bring the lowest HOMO–LUMO gap of 1.49 and 1.50 eV. This is in contrast to aforementioned pyridine series a in which the lowest Δ*E* has been recorded for olefinic chromophores. Overall, by varying the π-system and *via N*-quaternization, the HOMO–LUMO gap in 1–9 may be tuned within the broad range of 3.01 to 1.49 eV.

### Electronic absorption spectra

**Fig. 4:**
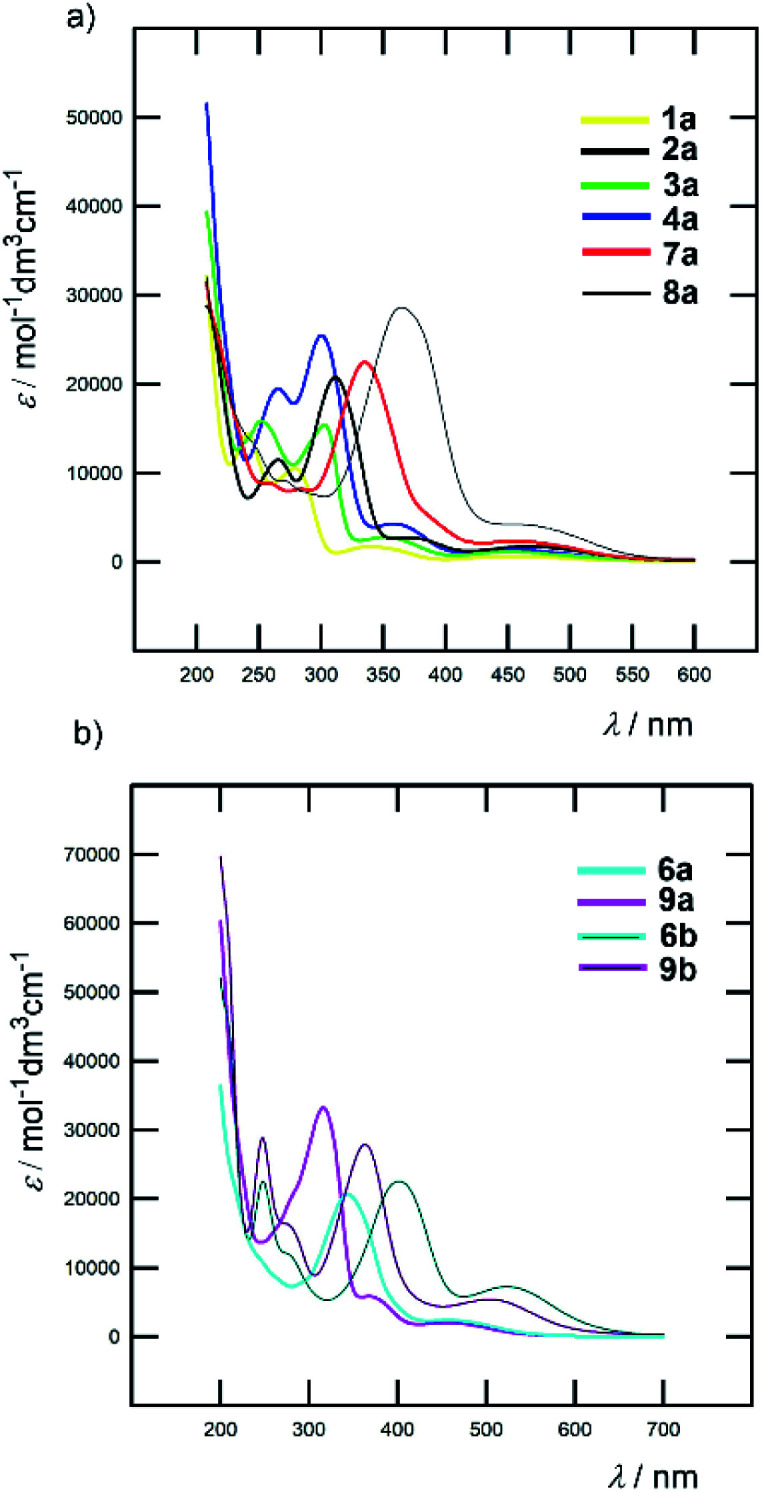
Effect of π-system extension (a) and *N*-quaternization (b) on optical properties of selected Fc–π–Py and Fc–π–Py^+^ chromophores (ACN).

All Fc–π–Py and Fc–π–Py^+^ derivatives 1–9 are intensively coloured compounds. Their colour ranges from orange to dark red, while none emissive properties were observed. Table 1 lists the longest wavelength absorption maxima and the corresponding molar absorption coefficients and Fig. 4 shows absorption spectra of representative chromophores in acetonitrile (ACN); see the ESI† for complete spectra. For Fc-derivatives, two electronic transitions and two particularly developed bands are generally seen.^18,26^ Whereas the low-energy (LE) band corresponds to the transition from the Fe-centred HOMO to the LUMO, the high-energy (HE) band originates from the interaction of the Cp/π-localized HOMO−3 and the LUMO. According to this model, an extension of the π-system should primarily affect the position of the HE band. Indeed, when comparing the *λ*_max_ values of the HE and LE bands listed in Table 1, the principal changes are seen in the position of the HE band (Δ*λ*^HE^_max_ is 86 and 113 nm for Fc–π–Py and Fc–π–Py^+^ chromophores). When going from parent 1a/1b and gradually extending and varying the π-system, the HE band shifted from 280/313 to 366/426 nm of 8a/8b bearing polarizable and planarized ethenylthiophene π-linker (Fig. 4a). On the contrary, variation in the *λ*^LE^_max_ is rather low (8/48 nm within the series a and b). Upon *N*-quaternization (a → b), both HE and LE bands shifted bathochromically by 30–60 and 70–90 nm, respectively. The *N*-quaternization affected the position of the LE band more significantly; see Fig. 4b for representative examples.

Besides acetonitrile, the spectra of pyridine chromophores were also recorded in dichloromethane (DCM). An inspection of the *λ*_max_ values gathered in Table S3† revealed that the solvatochromic behaviour of 1a–9a is diminished with the Δ*λ*^HE/LE^_max_ of 1–3/1–14 nm. On the contrary, the ionic Fc–π–Py^+^ chromophores 1b–9b showed hypsochromically-shifted HE and LE bands with increasing polarity of the solvent (Table S3†). For instance, the HE/LE bands of chromophore 8b shifted from 455/558 to 426/532 and 432/437 nm when going from DCM to ACN and MeOH, respectively. This negative solvatochromism, a typical feature of pyridinium salts,^27^ indicates that the excited state of Fc–π–Py^+^ chromophores (b) is less polar as compared to the ground state.

### DFT analysis

Pyridine as well as pyridinium derivatives 1–9 were theoretically investigated at the DFT level by using the Gaussian® 16 software package.^28^ Their geometries were optimized using the DFT B3LYP/6-311+G(2df,p) method; energies of the frontier molecular orbitals and ground-state dipole moments *μ* were calculated using DFT B3LYP/6-311+G(2df,p) level with acetonitrile as solvent. First hyperpolarizabilities *β* were calculated using DFT B3LYP/6-311+G(2df,p) method in vacuum at 1064 nm. The electronic absorption spectra and the corresponding longest-wavelength absorption maxima and electron transitions were gained by TD-DFT B3LYP/6-311+G(2df,p) (*n*_states_ = 8) method. All calculated data are gathered in Table 2.

**Table 2:** DFT calculated properties of chromophores 1a–9a and 1b–9b^a^

Comp.	*E*_HOMO−3_ [eV]	*E*_HOMO−2_ [eV]	*E*_HOMO−1_ [eV]	*E*_HOMO_ [eV]	*E*_LUMO_ [eV]	*E*_LUMO+1_ [eV]	*E*_LUMO+2_ [eV]	*E*_LUMO+3_ [eV]	*μ* [D]	*λ* ^HE/LE^_max_ [nm (eV)]	*β* (−2ω, ω, ω)^b^ [× 10^−30^ esu]
1a	−6.71	−6.52	−5.77	−5.73	−1.49	−0.77	−0.56	−0.45	4.6	324 (3.83)/—	7
2a	−6.54	−6.25	−5.74	−5.64	−1.93	−0.72	−0.59	−0.51	5.3	362 (3.43)/592 (2.09)	31
3a	−6.66	−6.47	−5.83	−5.77	−1.91	−0.84	−0.69	−0.63	5.2	342 (3.63)/—	28
4a	−6.50	−6.26	−5.69	−5.62	−1.79	−0.85	−0.66	−0.62	4.8	352 (3.52)/—	33
5a	−6.49	−6.02	−5.69	−5.58	−2.23	−0.84	−0.75	−0.68	5.7	382 (3.25)/580 (2.14)	124
6a	−6.52	−6.17	−5.72	−5.63	−2.19	−0.95	−0.87	−0.76	5.9	382 (3.25)/589 (2.10)	101
7a	−6.54	−6.06	−5.75	−5.60	−2.06	−0.85	−0.65	−0.60	5.6	385 (3.22)/591 (2.10)	45
8a	−6.53	−5.91	−5.75	−5.50	−2.31	−0.95	−0.72	−0.60	6.5	420 (2.95)/598 (2.07)	137
9a	−6.56	−6.02	−5.77	−5.60	−2.27	−1.06	−0.85	−0.62	6.4	393 (3.15)/593 (2.09)	118
1b	−7.14	−6.81	−6.07	−6.06	−2.71	−1.71	−0.94	−0.82	12.5	374 (3.32)/618 (2.00)	24
2b	−6.81	−6.59	−5.97	−5.89	−2.98	−1.66	−0.90	−0.78	17.6	387 (3.20)/642 (1.93)	107
3b	−6.97	−6.74	−6.04	−6.02	−3.10	−1.73	−0.94	−0.89	18.4	375 (3.31)/624 (1.99)	176
4b	−6.64	−6.47	−5.81	−5.75	−2.95	−1.79	−1.10	−0.90	24.1	396 (3.13)/612 (2.03)	116
5b	−6.59	−6.27	−5.80	−5.71	−3.14	−1.68	−1.39	−0.86	28.8	445 (2.79)/629 (1.97)	255
6b	−6.61	−6.39	−5.81	−5.74	−3.28	−1.84	−1.47	−1.04	29.7	445 (2.79)/640 (1.94)	4166
7b	−6.71	−6.37	−5.91	−5.80	−3.04	−1.71	−1.20	−0.75	20.5	425 (2.92)/633 (1.96)	2218
8b	−6.66	−6.14	−5.88	−5.71	−3.14	−1.63	−1.53	−0.72	25.0	467 (2.66)/642 (1.93)	503
9b	−6.68	−6.25	−5.89	−5.76	−3.22	−1.77	−1.62	−0.73	26.8	463 (2.68)/639 (1.94)	203

a All data were calculated at the DFT B3LYP/6-311+G(2df,p) level with acetonitrile as solvent.

b In vacuum at 1064 nm.

Relatively weak correlations have been found for the DFT-calculated and electrochemically-derived energies of the HOMO (Fig. S16 and S18†), which implies that the redox process, and subsequently also the ICT, are not solely dominated by a transition from the HOMO. This observation agrees with the aforementioned theory on multiple interaction of the ferrocene donor. On the contrary, the LUMO energies showed tight correlations (Fig. S17 and S19†), which means that the acceptor-centred LUMO is directly involved in the redox process and the ICT. Hence, the DFT-calculated LUMO energies obey the same trends as seen for the electrochemical *E*_LUMO_ values. A visualization of the frontier molecular orbitals (HOMO → HOMO−4 and LUMO → LUMO+4) are provided for the particular chromophore in Fig. S20–S37.† Except for acetylenic compounds 3b, the HOMO and HOMO−1 are solely localized on the ferrocene Fe atom. On the contrary, the HOMO−2 is distributed further on the ferrocene Cp ring(s) and may also involve the appended π-system.

Surprisingly and in contrast to the aforementioned theory, the HOMO−3 mostly occupies the ferrocene Fe atom with a minimal distribution on the Cp ring(s). The HOMO−4 is spread over the whole Fc moiety and the appended π-system. The LUMO is localized either on the pyridine/pyridinium acceptor or on the appended π-linker. The higher vacant orbitals are variously distributed over the whole π-system including the ferrocene Cp ring. As expected, variation in the calculated ground state dipole moment within the series a is minimal (4.6–6.5 D), which reflects only extension of the π-system. However, upon *N*-quaternization, the dipole moment has significantly raised to 12.5–29.7 D, which is due to ionic nature of the pyridinium acceptor including bulky I^−^ anion in compounds b.

The calculated electronic absorption spectra along with the spectra recorded in ACN are shown in Fig. S38 and 39,† while the longest-wavelength absorption maxima are listed in Table 2. As compared to the experimental data, the calculated spectra are slightly bathochromically shifted but possess the same features and bands. The spectra of chromophores 1a, 3a and 4a with short, less polarizable or nonplanar π-linker possess diminished LE band. An analysis of the transitions forming these bands revealed close to zero oscillator strengths. The remaining Fc–π–Py chromophores possess both bands. The LE bands are dominated by transitions from the HOMO−1 or the HOMO−2 to the LUMO or higher vacant orbitals, typically, HOMO−1/2 → LUMO+2/3. Considering the different localizations of both HOMOs on the Fe-atom and the adjacent Cp ring (see above and Fig. S20–37†), mixed interactions were found to form the LE band of 2a and 5a–9a. The HE bands of compounds 1a–9a are formed by the HOMO → LUMO and eventually the HOMO−2 → LUMO transitions and again both Fe → Py and Cp → Py interactions were identified. Hence, ferrocene push–pull molecules a bearing weak pyridine acceptor do not obey the aforementioned model on two separate interactions resulting in two bands. On the contrary, the spectra of pyridinium chromophores 1b–9b feature bathochromically and hyperchromically shifted LE bands, which are mostly formed by the HOMO → LUMO transition eventually also accompanied by the transition from the HOMO−1, thus both Fe-centered orbitals. The HE band is composed of two dominant transitions – HOMO−2 → LUMO and HOMO−3 → LUMO. Hence, we can conclude that in Fc–π–Py^+^ chromophores b two separate Fe → Py^+^ and Cp → Py^+^ interactions resulting in the LE and HE bands can be identified.

The first-order hyperpolarizabilities *β* (−2ω, ω, ω) were calculated for both series of chromophores (Table 2). Considering the overestimated *β* values of 6b/7b as outliers, the calculated hyperpolarizabilities are slightly higher to those calculated for previous Fc–π–imidazole derivatives.^18*a*^ The hyperpolarizabilities increase with enlarged and planarized π-system. For instance, *β* values of 1a, 2a and 3a (7, 31 and 28 × 10^−30^ esu) reflect extension and planarization by adding olefinic spacer as compared to the acetylenic one. However, when interconnecting the electronegative acetylene and pyridinium moieties as in 3b, a strong electron-withdrawing group is formed with increased hyperpolarizability to 176 × 10^−30^ esu. A comparison of 4a and 7a clearly illustrates a beneficial role of 2,5-thienylene over 1,4-phenylene spacers. In general, *N*-quaternized chromophores in series b showed enhanced nonlinearity with the largest first-order hyperpolarizability of 503 × 10^−30^ esu calculated for 8b bearing polarizable thiophene and olefinic π-system.

## Conclusion

A series of push–pull molecules with ferrocene donor, pyridine/pyridinium acceptors and systematically enlarged π-system has been designed and prepared. The synthesis involved Suzuki–Miyaura, Sonogashira or Heck cross-coupling reactions. Two chromophores were successfully crystallized and their molecular structure was confirmed by X-ray analysis. Electrochemical measurements revealed that structural tuning *via* π-system extension and *N*-quaternization principally affects the LUMO with the almost steady HOMO. The HOMO–LUMO gap of chromophores 1–9 can be tuned within a broad range of 3.01 to 1.49 eV. Two LE and HE bands were identified in the electronic absorption spectra; the latter was significantly affected by the π-system extension. The *N*-quaternization red-shifted both bands by *ca.* 30–90 nm. A diminished solvatochromism was observed for pyridine chromophores 1a–9a, while pyridinium compounds 1b–9b showed distinct negative solvatochromism. The performed DFT analysis corroborated the experimental observations and especially shed light on different D–A interactions seen for Fc–π–Py and Fc–π–Py^+^ chromophores. The ferrocene turned out to be an electron donor with two principal charge-transfers that, based on the appended acceptor, are variously employed.

## Experimental

All reagents and solvents were reagent grade and were purchased from Penta, Aldrich, and TCI and used as received. The starting ferroceneboronic acid 10, 4-pyridinylboronic acid 11, vinylferrocene 12, 4-vinylpyridine 13, ethynylferrocene 14, 4-ethynylpyridine hydrochloride 15 and 4-iodopyridine 16 were commercially available. Compound 19 was reported previously.^18*b*^ All cross-coupling reactions were carried out in flame-dried flasks under an argon atmosphere. Thin layer chromatography (TLC) was conducted on aluminum sheets coated with silica gel 60 F254 with visualization by a UV lamp (254 or 360 nm). Column chromatography was carried out with silica gel 60 (particle size 0.040–0.063 mm, 230–400 mesh) and commercially available solvents. ^1^H and ^13^C NMR spectra were recorded on a Bruker AVANCE II/III 400/500 spectrometer (400/500 MHz or 100/125 MHz, respectively). Chemical shifts are reported in ppm relative to the signal of Me_4_Si (0.00 ppm). The residual solvent signal was used as an internal reference (CDCl_3_ 7.25 and 77.23 ppm; DMSO-*d*_6_ 2.55 and 39.52). Apparent resonance multiplicities are described as s (singlet), d (doublet), dd (doublet of doublet), and m (multiplet). ^1^H NMR signals of pyridine, thiophene and cyclopentadienyl are denoted as Py, Th and Cp. The coupling constants, *J*, are reported in Hertz (Hz). High-resolution MALDI mass spectroscopy data were collected on an LTQ Orbitrap XL. Absorption spectra were measured on a UV/vis HP 8453 spectrophotometer at room temperature.

### General procedure for the Suzuki–Miyaura cross-coupling (compounds 1a, 4a, 7a and 9a)

In a Schlenk flask, ferroceneboronic acid 10 (276 mg, 1.0 mmol) or 4-pyridinylboronic acid 11 (148 mg, 1.2 mmol) and corresponding halogen derivative (1.0 mmol) were dissolved in 1,2-dimethoxyethane (10 mL) or in a mixture of 1,4-dioxane/H_2_O (25 mL, 4 : 1). Argon was bubbled through the solution for 10 min, whereupon PdCl_2_[Fe(PPh_3_)_2_]_2_ (35 mg, 0.05 mmol, 5%) along with a solution of sodium hydroxide (1 mL of 10% solution, 3 mmol) or PdCl_2_(PPh_3_)_2_ (35 mg, 0.05 mmol, 5%) and K_2_CO_3_ (150 mg, 1.1 mmol) were added and the resulting reaction mixture was stirred at 85 °C for 12 h. The reaction mixture was diluted with water (100 mL) and extracted with CH_2_Cl_2_ (3 × 50 mL). The combined organic extracts were washed with water, dried over anhydrous Na_2_SO_4_, the solvents were evaporated *in vacuo* and the crude product was purified by column chromatography.

### General procedure for the Heck cross-coupling (compounds 2a, 5a, 8a)

In a Schlenk flask, vinylferrocene 12 (424 mg, 2.0 mmol) or vinylpyridine 13 (210 mg, 2.0 mmol) and corresponding iodo derivative (2.0 mmol) were dissolved in a DMF (10 mL). Ethyldiisopropylamine (0.4 mL, 2.3 mmol) was added and argon was bubbled through the solution for 10 min, whereupon [*t*Bu_3_P]_2_Pd (50 mg, 0.1 mmol, 5%) was added and the resulting reaction mixture was stirred at 85 °C for 12 h. The reaction mixture was diluted with water (100 mL) and extracted with EtAc (3 × 50 mL). The combined organic extracts were washed with water, dried over anhydrous Na_2_SO_4_, the solvents were evaporated *in vacuo* and the crude product was purified by column chromatography.

### General procedure for the Sonogashira cross-coupling (compounds 3a and 6a)

In a Schlenk flask, ethynylferrocene 14 (315 mg, 1.5 mmol) or ethynylpyridine hydrochloride 15 (210 mg, 1.5 mmol) and corresponding iodo derivate (1.0 mmol) were dissolved in a THF (15 mL). Ethyldiisopropylamine (2 mL, 11.5 mmol)) was added and argon was bubbled through the solution for 10 min, whereupon PdCl_2_(PPh_3_)_2_ (35 mg, 0.05 mmol, 5%) and copper iodide (15 mg, 0.025 mmol, 2.5%) were added and the resulting reaction mixture was stirred at 60 °C for 12 h. The reaction mixture was diluted with water (100 mL) and extracted with CH_2_Cl_2_ (3 × 50 mL). The combined organic extracts were dried over anhydrous Na_2_SO_4_, the solvents were evaporated *in vacuo* and the crude product was purified by column chromatography.

### General procedure for quaternization (compounds 1b–9b)

In a 10 mL flask, ferrocenylpyridine derivates 1a–9a (0.3 mmol) and methyliodide (6 mL, 9.7 mmol) were stirred under reflux for 1 h. The reaction mixture was cooled to 25 °C and the formed quaternary salt was filtered off and washed with ether.

#### 4-Ferrocenylpyridine (1a)

The title compound was prepared from 10 (230 mg, 1.0 mmol) and 16 (246 mg, 1.2 mmol) following the general method for the Suzuki–Miyaura reaction (DME, PdCl_2_[Fe(PPh_3_)_2_]_2_, NaOH solution). The crude product was purified by column chromatography (SiO_2_, CH_2_Cl_2_/EtAc, 5 : 2). Yield: 126 mg (48%); orange solid; *R*_f_ = 0.20 (SiO_2_; CH_2_Cl_2_/EtAc, 5 : 2); mp 139–140 °C; ^1^H NMR (400 MHz, CDCl_3_, 25 °C): *δ* = 4.03 (s, 5H; Cp), 4.42 (bs, 2H; Cp), 4.72 (bs, 2H, Cp), 7.33 (d, *J* = 4 Hz, 2H; PyH), 8.50 ppm (bs, 2H; PyH); ^13^C NMR (100 MHz, CDCl_3_, 25 °C): *δ* = 149.77, 148.98, 120.95, 81.09, 70.48, 70.18, 67.06 ppm; FTIR (HATR): *ν̃* = 3032, 1590, 1414, 1104, 996, 821, 484 cm^−1^; HR-FT-MALDI-MS (DHB): *m*/*z* calcd for C_15_H_13_FeN [M + H]^+^ 264.04757; found: 264.04713.

#### (*E*)-4-(2′-Ferrocenylethenyl)pyridine (2a)

The title compound was prepared from 12 (424 mg, 2.0 mmol) and 16 (410 mg, 2.0 mmol) following the general method for the Heck reaction. The crude product was purified by column chromatography (SiO_2_, CH_2_Cl_2_–EtAc, 5 : 2). Yield: 352 mg (61%); dark red solid; *R*_f_ = 0.35 (SiO_2_; CH_2_Cl_2_–EtAc, 5 : 2); mp 154–156 °C; ^1^H NMR (500 MHz, CDCl_3_, 25 °C): *δ* = 4.14 (s, 5H; Cp), 4.35 (bs, 2H; Cp), 4.49 (bs, 2H; Cp), 6.58 (d, *J* = 16 Hz, 1H; CH), 7.12 (d, *J* = 16 Hz, 1H; CH), 7.28 (bs, 2H; Py), 8.51 ppm (bs, 2H; Py); ^13^C NMR (125 MHz, CDCl_3_, 25 °C): *δ* = 149.90, 145.51, 132.94, 123.05, 120.36, 81.60, 70.06, 69.53, 67.61 ppm; FTIR (HATR): *ν̃* = 3080, 1625, 1585, 1410, 1191, 805, 476 cm^−1^; HR-FT-MALDI-MS (DHB): *m*/*z* calcd for C_17_H_15_FeN [M + H]^+^ 290.06322; found: 290.06265.

#### 4-Ferrocenylethynylpyridine (3a)

The title compound was prepared from 14 (315 mg, 1.5 mmol) and 16 (205 mg, 1.0 mmol) following the general method for the Sonogashira reaction. The crude product was purified by column chromatography (SiO_2_, CH_2_Cl_2_/EtAc, 5 : 2). Yield: 195 mg (68%); dark red solid; *R*_f_ = 0.45 (SiO_2_; CH_2_Cl_2_/EtAc, 5 : 2); mp 154–156 °C; ^1^H NMR (500 MHz, CDCl_3_, 25 °C): *δ* = 4.24 (s, 5H; Cp), 4.29 (bs, 2H; Cp), 4.53 (bs, 2H, Cp), 7.33 (bs, 2H; Py), 8.57 ppm (bs, 2H; Py); ^13^C NMR (125 MHz, CDCl_3_, 25 °C): *δ* = 149.76, 132.25, 125.52, 94.32, 83.54, 71.89, 70.23, 69.58, 63.71 ppm; FTIR (HATR): *ν̃* = 3062, 2206, 1588, 1407, 1168, 812, 482 cm^−1^; HR-FT-MALDI-MS (DHB): *m*/*z* calcd for C_17_H_13_FeN [M + H]^+^ 288.04757; found: 288.04578.

#### 4-(4′-Ferrocenylphenyl)pyridine (4a)

The title compound was prepared from 11 (148 mg, 1.2 mmol) and 18 (388 mg, 1.0 mmol) following the general method for the Suzuki–Miyaura reaction (1,4-dioxane/H_2_O, PdCl_2_(PPh_3_)_2_, K_2_CO_3_). The crude product was purified by column chromatography (SiO_2_, CH_2_Cl_2_/EtAc, 5 : 2). Yield: 180 mg (53%); orange-red solid; *R*_f_ = 0.25 (SiO_2_; CH_2_Cl_2_/EtAc, 5 : 2); mp 218–219 °C; ^1^H NMR (400 MHz, CDCl_3_, 25 °C): *δ* = 4.06 (s, 5H; Cp), 4.36 (bs, 2H; Cp), 4.69 (bs, 2H; Cp), 7.54–7.57 (m, 6H; ArH, Py), 8.66 ppm (bs, 2H; Py); ^13^C NMR (125 MHz, CDCl_3_, 25 °C): *δ* = 150.21, 148.04, 140.86, 135.18, 126.91, 126.66, 84.09, 69.73, 69.39, 66.63 ppm; FTIR (HATR): *ν̃* = 3029, 1588, 1403, 1230, 994, 806, 491 cm^−1^; HR-FT-MALDI-MS (DHB): *m*/*z* calcd for C_21_H_17_FeN [M + H]^+^ 340.07887; found: 340.07854.

#### (*E*)-4-(4′-Ferrocenylstyryl)pyridine (5a)

The title compound was prepared from 13 (210 mg, 2.0 mmol) and 18 (776 mg, 2.0 mmol) following the general method for the Heck reaction. The crude product was purified by column chromatography (SiO_2_, CH_2_Cl_2_/EtAc, 5 : 2). Yield: 387 mg (53%); red solid; *R*_f_ = 0.40 (SiO_2_; CH_2_Cl_2_/EtAc, 5 : 2); mp 236–238 °C; ^1^H NMR (500 MHz, CDCl_3_, 25 °C): *δ* = 4.05 (s, 5H; Cp), 4.36 (bs, 2H; Cp), 4.69 (bs, 2H; Cp), 7.01 (d, *J* = 16 Hz, 1H; CH), 7.29 (d, *J* = 16 Hz, 1H; CH), 7.40 (bs, 2H; Py), 7.45–7.49 (m, 4H; ArH), 8.59 ppm (bs, 2H; Py); ^13^C NMR (125 MHz, CDCl_3_, 25 °C): *δ* = 150.15, 145.11, 140.62, 133.72, 133.30, 127.27, 126.45, 124.97, 120.96, 84.48, 69.95, 69.49, 66.65 ppm; FTIR (HATR): *ν̃* = 3020, 1582, 1411, 1276, 1106, 826, 475 cm^−1^; HR-FT-MALDI-MS (DHB): *m*/*z* calcd for C_23_H_19_FeN [M + H]^+^ 366.09452; found: 366.09330.

#### 4-((4′-Ferrocenylphenyl)ethynyl)pyridine (6a)

The title compound was prepared from 15 (208 mg, 1.5 mmol) and 18 (388 mg, 1.0 mmol) following the general method for the Sonogashira reaction. The crude product was purified by column chromatography (SiO_2_, CH_2_Cl_2_/EtAc, 5 : 2). Yield: 290 mg (80%); red solid; *R*_f_ = 0.45 (SiO_2_; CH_2_Cl_2_/EtAc, 5 : 2); mp 225–226 °C; ^1^H NMR (500 MHz, CDCl_3_, 25 °C): *δ* = 4.05 (s, 5H; Cp), 4.38 (bs, 2H; Cp), 4.68 (bs, 2H; Cp), 7.39 (bs, 2H; Py), 7.46 (bs, 4H; ArH), 8.60 ppm (bs, 2H; Py); ^13^C NMR (125 MHz, CDCl_3_, 25 °C): *δ* = 149.72, 141.34, 132.09, 131.97, 126.03, 125.67, 119.11, 94.84, 86.87, 83.91, 69.91, 69.72, 66.74 ppm; FTIR (HATR): *ν̃* = 3082, 2212, 1581, 1407, 1105, 816, 481 cm^−1^; HR-FT-MALDI-MS (DHB): *m*/*z* calcd for C_23_H_17_FeN [M]^+^ 363.07104; found: 363.07083.

#### 4-(5′-Ferrocenylthiophen-2-yl)pyridine (7a)

The title compound was prepared from 11 (148 mg, 1.2 mmol) and 19 (394 mg, 1.0 mmol) following the general method for the Suzuki–Miyaura reaction (1,4-dioxane/H_2_O, PdCl_2_(PPh_3_)_2_, K_2_CO_3_). The crude product was purified by column chromatography (SiO_2_, CH_2_Cl_2_/EtAc, 5 : 2). Yield: 221 mg (64%); dark red solid; *R*_f_ = 0.30 (SiO_2_; CH_2_Cl_2_/EtAc, 5 : 2); mp 187–188 °C; ^1^H NMR (500 MHz, CDCl_3_, 25 °C): *δ* = 4.11 (s, 5H; Cp), 4.34 (bs, 2H; Cp), 4.61 (bs, 2H; Cp), 7.01 (d, *J* = 3 Hz, 1H; ThH), 7.34 (d, *J* = 3 Hz, 1H; ThH), 7.45 (bs, 2H; Py), 8.57 ppm (bs, 2H; Py); ^13^C NMR (125 MHz, CDCl_3_, 25 °C): *δ* = 150.38, 146.34, 141.64, 137.96, 126.00, 123.51, 119.35, 79.13, 70.31, 69.31, 67.19 ppm; FTIR (HATR): *ν̃* = 3075, 1584, 1409, 987, 799, 485 cm^−1^; HR-FT-MALDI-MS (DHB): *m*/*z* calcd for C_19_H_15_FeNS [M + H]^+^ 346.03529; found: 346.03502.

#### (*E*)-4-(2′-(5′′-Ferrocenylthiophen-2′′-yl)ethenyl)pyridine (8a)

The title compound was prepared from 13 (210 mg, 2.0 mmol) and 19 (788 mg, 2.0 mmol) following the general method for the Heck reaction. The crude product was purified by column chromatography (SiO_2_, CH_2_Cl_2_/EtAc, 5 : 2). Yield: 441 mg (64%); dark red solid; *R*_f_ = 0.35 (SiO_2_; CH_2_Cl_2_/EtAc, 5 : 2); mp 164–165 °C; ^1^H NMR (400 MHz, CDCl_3_, 25 °C): *δ* = 4.11 (s, 5H; Cp), 4.33 (bs, 2H; Cp), 4.59 (bs, 2H; Cp), 6.72 (d, *J* = 16 Hz, 1H; CH), 6.91–6.95 (m, 2H; CH, Th), 7.31–7.38 (m, 3H; Th, Py), 8.58 ppm (bs, 2H; Py); ^13^C NMR (125 MHz, CDCl_3_, 25 °C): *δ* = 150.24, 145.08, 144.70, 139.10, 129.25, 126.52, 123.98, 122.86, 120.68, 79.37, 70.29, 69.28, 67.12 ppm; FTIR (HATR): *ν̃* = 3077, 1587, 1410, 1032, 942, 802, 477 cm^−1^; HR-FT-MALDI-MS (DHB): *m*/*z* calcd for C_21_H_17_FeNS [M + H]^+^ 372.05094; found: 372.05046.

#### 4-((5′-Ferrocenylthiophen-2′-yl)ethynyl)pyridine (9a)

The title compound was prepared from 10 (230 mg, 1.0 mmol) and 17 (318 mg, 1.2 mmol) following the general method for the Suzuki–Miyaura reaction (DME, PdCl_2_[Fe(PPh_3_)_2_]_2_, NaOH solution). The crude product was purified by column chromatography (SiO_2_, CH_2_Cl_2_/EtAc, 5 : 2). Yield: 181 mg (49%); orange red solid; *R*_f_ = 0.45 (SiO_2_; CH_2_Cl_2_/EtAc, 5 : 2); mp 149–150 °C; ^1^H NMR (500 MHz, CDCl_3_, 25 °C): *δ* = 4.09 (s, 5H; Cp), 4.32 (t, *J* = 2 Hz, 2H; Cp), 4.58 (t, *J* = 2 Hz, 2H; Cp), 6.90 (d, *J* = 4 Hz, 1H; Th), 7.15 (d, *J* = 4 Hz, 1H; Th), 7.35 (bs, 2H; Py), 8.58 ppm (bs, 2H; Py); ^13^C NMR (125 MHz, CDCl_3_, 25 °C): *δ* = 149.68, 147.89, 134.19, 131.63, 125.14, 122.37, 118.80, 90.84, 88.54, 78.70, 70.35, 69.41, 67.28 ppm; FTIR (HATR): *ν̃* = 3073, 2183, 1588, 1407, 1029, 803, 500 cm^−1^; HR-FT-MALDI-MS (DHB): *m*/*z* calcd for C_21_H_15_FeNS [M + H]^+^ 370.03529; found: 370.03484.

#### 1-Methyl-4-ferrocenylpyridinium iodide (1b)

The title compound was prepared from 1a (79 mg) following the general method for the quaternization. Yield: 119 mg (98%); dark red solid; ^1^H NMR (500 MHz, DMSO-*d*_6_, 25 °C): *δ* = 4.15–4.23 (m, 8H; Cp, CH_3_), 4.79 (bs, 2H; Cp), 5.29 (bs, 2H, Cp), 8.07 (bs, 2H; Py), 8.66 ppm (bs, 2H; Py); ^13^C NMR (125 MHz, DMSO-*d*_6_, 25 °C): *δ* = 159.73, 144.52, 122.61, 76.55, 73.84, 71.16, 69.34, 46.92 ppm; FTIR (HATR): *ν̃* = 3009, 1633, 1528, 1187, 998, 831, 482 cm^−1^; HR-FT-MALDI-MS (DHB): *m*/*z* calcd for C_16_H_16_FeN [M]^+^ 278.06322; found: 278.06257.

#### 1-Methyl-(*E*)-4-(2′-ferrocenylethenyl)pyridinium iodide (2b)

The title compound was prepared from 2a (87 mg) following the general method for the quaternization. Yield: 127 mg (98%); black solid; ^1^H NMR (500 MHz, DMSO-*d*_6_, 25 °C): *δ* = 4.22 (s, 3H; CH_3_), 4.27 (s, 5H; Cp), 4.65 (bs, 2H; Cp), 4.79 (bs, 2H; Cp), 7.02 (d, *J* = 16 Hz, 1H; CH), 7.93 (d, *J* = 16 Hz, 1H; CH), 8.11 (d, *J* = 6 Hz, 2H; Py), 8.79 ppm (d, *J* = 6 Hz, 2H; Py); ^13^C NMR (125 MHz, DMSO-*d*_6_, 25 °C): *δ* = 152.89, 145.18, 143.55, 122.75, 120.25, 80.49, 71.96, 70.14, 69.14, 47.04 ppm; FTIR (HATR): *ν̃* = 3012, 1643, 1597, 1474, 1176, 836, 485 cm^−1^; HR-FT-MALDI-MS (DHB): *m*/*z* calcd for C_18_H_18_FeN [M]^+^ 304.07887; found: 304.07818.

#### 1-Methyl-4-ferrocenylethynylpyridinium iodide (3b)

The title compound was prepared from 3a (86 mg) following the general method for the quaternization. Yield: 122 mg (95%); dark red solid; ^1^H NMR (500 MHz, DMSO-*d*_6_, 25 °C): *δ* = 4.31 (s, 3H; CH_3_), 4.39 (s, 5H; Cp), 4.62 (bs, 2H; Cp), 4.80 (bs, 2H, Cp), 8.17 (bs, 2H; Py), 8.96 ppm (bs, 2H; Py); ^13^C NMR (125 MHz, DMSO-*d*_6_, 25 °C): *δ* = 145.70, 138.29, 128.61, 105.76, 83.42, 72.89, 71.52, 70.77, 61.15, 47.96 ppm; FTIR (HATR): *ν̃* = 3064, 2190, 1629, 1516, 1159, 820, 477 cm^−1^; HR-FT-MALDI-MS (DHB): *m*/*z* calcd for C_18_H_16_FeN [M]^+^ 302.06322; found: 302.06212.

#### 1-Methyl-4-(4′-ferrocenylphenyl)pyridinium iodide (4b)

The title compound was prepared from 4a (102 mg) following the general method for the quaternization. Yield: 124 mg (86%); orange-red solid; ^1^H NMR (500 MHz, DMSO-*d*_6_, 25 °C): *δ* = 4.10 (s, 5H; Cp), 4.36 (s, 3H; CH_3_), 4.53 (bs, 2H; Cp), 5.04 (bs, 2H; Cp), 7.83 (d, *J* = 9 Hz, 2H; ArH), 8.08 (d, *J* = 9 Hz, 2H; ArH), 8.57 (d, *J* = 7 Hz, 2H; Py), 9.01 ppm (d, *J* = 7 Hz, 2H; Py); ^13^C NMR (125 MHz, DMSO-*d*_6_, 25 °C): *δ* = 154.24, 145.82, 144.98, 130.66, 128.59, 127.11, 123.69, 82.90, 70.47, 70.10, 67.36, 47.34 ppm; FTIR (HATR): *ν̃* = 3062, 1601, 1508, 1234, 810, 503 cm^−1^; HR-FT-MALDI-MS (DHB): *m*/*z* calcd for C_22_H_20_FeN [M]^+^ 354.09452; found: 354.09429.

#### 1-Methyl-(*E*)-4-(4′-ferrocenylstyryl)pyridinium iodide (5b)

The title compound was prepared from 5a (110 mg) following the general method for the quaternization. Yield: 132 mg (87%); red solid; ^1^H NMR (500 MHz, DMSO-*d*_6_, 25 °C): *δ* = 4.08 (s, 5H; Cp), *δ* = 4.29 (s, 3H; CH_3_), 4.49 (bs, 2H; Cp), 4.96 (bs, 2H; Cp), 7.55 (d, *J* = 16 Hz, 1H; CH), 7.69–7.74 (m, 4H; ArH), 8.05 (d, *J* = 16 Hz, 2H; CH), 8.26 (d, *J* = 7 Hz, 2H; Py), 8.89 ppm (d, *J* = 7 Hz, 2H; Py); ^13^C NMR (125 MHz, DMSO-*d*_6_, 25 °C): *δ* = 153.12, 145.46, 142.80, 141.15, 132.97, 128.82, 126.69, 123.70, 122.43, 83.77, 70.14, 70.03, 67.06, 47.29 ppm; FTIR (HATR): *ν̃* = 3013, 1597, 1520, 1180, 977, 821, 490 cm^−1^; HR-FT-MALDI-MS (DHB): *m*/*z* calcd for C_24_H_22_FeN [M]^+^ 380.11017; found: 380.10970.

#### 1-Methyl-4-((4′-ferrocenylphenyl)ethynyl)pyridinium iodide (6b)

The title compound was prepared from 6a (109 mg) following the general method for the quaternization. Yield: 138 mg (91%); orange-red solid; ^1^H NMR (500 MHz, DMSO-*d*_6_, 25 °C): *δ* = 4.01 (s, 5H; Cp), 4.36 (s, 3H; CH_3_), 4.52 (bs, 2H; Cp), 4.98 (bs, 2H; Cp), 7.67 (d, *J* = 8 Hz, 2H; ArH), 7.74 (d, *J* = 8 Hz, 2H; ArH), 8.28 (d, *J* = 7 Hz, 2H; Py), 9.03 ppm (d, *J* = 7 Hz, 2H; Py); ^13^C NMR (125 MHz, DMSO-*d*_6_, 25 °C): *δ* = 145.95, 143.79, 138.96, 133.04, 129.12, 126.62, 116.98, 103.58, 86.31, 83.09, 70.44, 70.11, 67.25, 48.15 ppm; FTIR (HATR): *ν̃* = 2991, 2213, 1627, 1527, 1140, 821, 486 cm^−1^; HR-FT-MALDI-MS (DHB): *m*/*z* calcd for C_24_H_20_FeN [M]^+^ 378.09452; found: 378.09409.

#### 1-Methyl-4-(5′-ferrocenylthiophen-2-yl)pyridinium iodide (7b)

The title compound was prepared from 7a (104 mg) following the general method for the quaternization. Yield: 137 mg (94%); dark red solid; ^1^H NMR (500 MHz, DMSO-*d*_6_, 25 °C): *δ* = 4.17–4.24 (m, 8H; Cp, CH_3_), 4.56 (bs, 2H; Cp), 4.90 (bs, 2H; Cp), 7.48 (bs, 1H; Th), 8.17 (bs, 1H; Th), 8.27 (bs, 2H; Py), 8.83 ppm (bs, 2H; Py); ^13^C NMR (125 MHz, DMSO-*d*_6_, 25 °C): *δ* = 152.40, 146.88, 144.72, 132.99, 132.91, 125.13, 120.50, 76.89, 69.84, 69.78, 67.08, 46.20 ppm; FTIR (HATR): *ν̃* = 3022, 1631, 1417, 1187, 812, 488 cm^−1^; HR-FT-MALDI-MS (DHB): *m*/*z* calcd for C_20_H_18_FeNS [M]^+^ 360.05094; found: 360.05045.

#### 1-Methyl-(*E*)-4-(2′-(5′′-ferrocenylthiophen-2′′-yl)ethenyl)pyridinium iodide (8b)

The title compound was prepared from 8a (111 mg) following the general method for the quaternization. Yield: 145 mg (94%); dark red solid; ^1^H NMR (500 MHz, DMSO-*d*_6_, 25 °C): *δ* = 4.17 (s, 5H; Cp), 4.25 (s, 3H; CH_3_), 4.52 (bs, 2H; Cp), 4.84 (bs, 2H; Cp), 7.11 (d, *J* = 16 Hz, 1H; CH), 7.29 (d, *J* = 4 Hz, 1H; Th), 7.39 (d, *J* = 4 Hz, 1H; Th), 8.18–8.23 (m, 3H, CH, Py), 8.82 ppm (d, *J* = 7 Hz, 2H; Py); ^13^C NMR (125 MHz, DMSO-*d*_6_, 25 °C): *δ* = 152.85, 148.86, 145.22, 138.23, 134.46, 133.88, 124.62, 123.17, 120.79, 79.63, 70.55, 70.22, 67.62, 47.15 ppm; FTIR (HATR): *ν̃* = 3014, 1596, 1417, 1184, 961, 811, 483 cm^−1^; HR-FT-MALDI-MS (DHB): *m*/*z* calcd for C_22_H_20_FeNS [M]^+^ 386.06659; found: 386.06630.

#### 1-Methyl-4-((5′-ferrocenylthiophen-2′-yl)ethynyl)pyridinium iodide (9b)

The title compound was prepared from 9a (111 mg) following the general method for the quaternization. Yield: 127 mg (83%); dark red solid; ^1^H NMR (500 MHz, DMSO-*d*_6_, 25 °C): *δ* = 4.09 (s, 5H; Cp), 4.26 (s, 3H; CH_3_), 4.46 (bs, 2H; Cp), 4.81 (bs, 2H; Cp), 7.27 (bs, 1H; Th), 7.54 (bs, 1H; Th), 8.16 (bs, 2H; Py), 8.92 ppm (bs, 2H; Py); ^13^C NMR (125 MHz, DMSO-*d*_6_, 25 °C): *δ* = 151.70, 145.80, 138.50, 138.23, 128.25, 124.30, 116.16, 97.56, 91.00, 77.82, 70.63, 70.35, 67.87, 48.04 ppm; FTIR (HATR): *ν̃* = 3058, 2184, 1629, 1467, 1210, 813, 485 cm^−1^; HR-FT-MALDI-MS (DHB): *m*/*z* calcd for C_22_H_18_FeNS [M]^+^ 384.05094; found: 384.05048.

## Author contributions

Funding acquisition: FB; investigation: JK, MK, OP, ZR; methodology: JK, FB; project administration: FB; writing – original draft: FB; writing – review & editing: FB.

## Conflicts of interest

There are no conflicts to declare.

## Supplementary Material

RA-011-D1RA08186A-s001

RA-011-D1RA08186A-s002
