# Supplementary material for: Ferrocene donor linked to pyridine/pyridinium acceptor via a systematically enlarged π-linker
Source: RSC Adv. 2021 Dec 3;11(61):38804–13. doi: 10.1039/d1ra08186a (PMC9044170; doi:10.1039/d1ra08186a)
Supplement: RA-011-D1RA08186A-s001 [file RA-011-D1RA08186A-s001.pdf]

Electronic Supplementary Material (ESI) for RSC Advances.
This journal is © The Royal Society of Chemistry 2021

Table of content

1. Synthesis and characterization 2

2. X-Ray analysis 3

3. Electrochemistry 9

4. Absorption spectra 13

5. DFT calculations 15

6. NMR spectra 29

1. Synthesis and characterization

4-((5´-Bromothiophen-2´-yl)ethynyl)pyridine 17

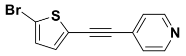

4-((5´-Bromothiophen-2´-yl)ethynyl)pyridine was synthesized via general procedure for Sonogashira cross-coupling from 5-bromo-2-iodothiophene[1] (576 mg, 2 mmol) and ethynylpyridine hydrochloride **15** (278 mg, 2 mmol). The crude product was purified by column chromatography (SiO_2, CH_2Cl_2-EtAc, 1:1). Yield: 390 mg (74 %); yellow solid; R_f = 0.45 (SiO_2; CH_2Cl_2-EtAc, 1:1); m.p. 131-132 °C; ^{1}H NMR (400 MHz, $CDCl_3$, 25 °C): δ = 6.99 (d, J = 4 Hz, 1H; Th), 7.08 (d, J = 4 Hz, 1H; Th), 7.33 (d, J = 5 Hz, 2H; Py), 8.60 ppm (bs, 2H; Py); ^{13}C NMR (100 MHz, $CDCl_3$, 25 °C): δ = 150.02, 133.76, 130.87, 130.55, 125.30, 123.87, 115.04, 91.48, 86.38 ppm; EI/MS (70EV): m/z (%): 265 (M^+,100), 263 (98), 184 (23), 140 (41); HR-FT-MALDI-MS (DHB): *m/z* calcd. for $C_{11}H_6BrNS$ $[M+H]^+$ 263.94826; found: 263.94794.

4-Iodophenylferrocene 18

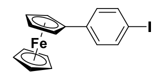

4-Iodophenylferrocene was synthesized via modification literature procedure.[2] 4-Iodoaniline (2.19 g, 10.0 mmol) was dissolved in a mixture of water (25 ml) and concentrated hydrochloric acid (3 ml). The resulting solution was cooled to 0 °C whereupon a solution of sodium nitrite (760 mg, 11.0 mmol) in water (50 ml) was added dropwise through a dropping funnel at a rate to keep the temperature below 5 °C. Subsequently, the reaction mixture was stirred for additional 30 min at 0 °C. A solution of ferrocene (1.86 g, 10.0 mmol) in toluene (100 ml) containing acetonitrile (3 ml) was added via a dropping funnel over a period of 15 min. After removal of the cooling bath, the reaction mixture was stirred for 12 h at 25 °C. The mixture was diluted with water (300 ml) and extracted with EtAc (3 × 100 ml). The combined organic layers were dried (Na_2SO_4) and the solvent was evaporated *in vacuo*. The crude product was purified by column chromatography (SiO_2, hexane). Yield: 700 mg (18 %); orange solid; R_f = 0.40 (SiO_2, hexane); m.p. 132-134 °C; ^{1}H NMR (400 MHz, $CDCl_3$, 25 °C): δ = 4.04 (s, 5H; Cp), 4.34 (bs, 2H; Cp), 4.62 (bs, 2H, Cp), 7.19 (d, J = 8 Hz, 2H; Ar), 7.58 ppm (d, J = 8 Hz, 2H; Ar); ^{13}C NMR (125 MHz, $CDCl_3$, 25 °C): δ = 139.17, 137.34, 127.91, 90.59, 84.22, 69.84, 69.38, 66.50 ppm; EI/MS (70EV): *m/z* (%): 388 (M^+,100), 261 (15), 205 (13), 139 (36); HR-FT-MALDI-MS (DHB): *m/z* calcd. for $C_{16}H_{13}FeI$ $[M]^+$ 387.94114; found: 387.94096.

[1] Y. Goldberg, H. Alper, *J. Org. Chem.* 1993, **58**, 3072.
[2] M. V. Makarov, V. P. Dyadchenko, M. Y. Antipin, *Russ. Chem. Bull. Int. Ed.* 2004, **53**, 2768.

2. X-Ray analysis

Table S1. Experimental details for **6a**.

Crystal data	
Chemical formula	$C_{23}H_{17}FeN$
M_r	363.23
Crystal system, space group	Monoclinic, $P2_1$
Temperature (K)	150
a, *b*, *c* (Å)	10.696 (4), 7.473 (2), 21.224 (7)
β (°)	94.182 (13)
V (Å^3)	1692.0 (9)
Z	4
Radiation type	Mo *K*α
μ (mm^{-1})	0.90
Crystal size (mm)	0.51 × 0.40 × 0.26
Data collection	
Diffractometer	Bruker D8 - Venture
Absorption correction	Multi-scan<br>*SADABS2016*/2 - Bruker AXS area detector scaling and absorption correction
T_{min}, T_{max}	0.647, 0.746
No. of measured, independent and observed [$I > 2\sigma(I)$] reflections	50731, 10207, 8477
R_{int}	0.049
$(\sin \theta/\lambda)_{max}$ (Å^{-1})	0.745
Refinement	
$R[F^2 > 2\sigma(F^2)]$, $wR(F^2)$, S	0.040, 0.075, 1.04
No. of reflections	10207
No. of parameters	452
No. of restraints	565
H-atom treatment	H-atom parameters constrained
$\Delta\rho_{max}$, $\Delta\rho_{min}$ (e Å^{-3})	0.34, −0.50
Absolute structure	Refined as an inversion twin.
Absolute structure parameter	0.129 (16)

Computer programs: Bruker Instrument Service vV6.2.3, *APEX3* v2018.1-0 (Bruker AXS), *SAINT* V8.38A (Bruker AXS Inc., 2016), *SHELXT* 2014/5 (Sheldrick, 2014), *SHELXL2017*/1 (Sheldrick, 2017), Bruker *SHELXTL*.

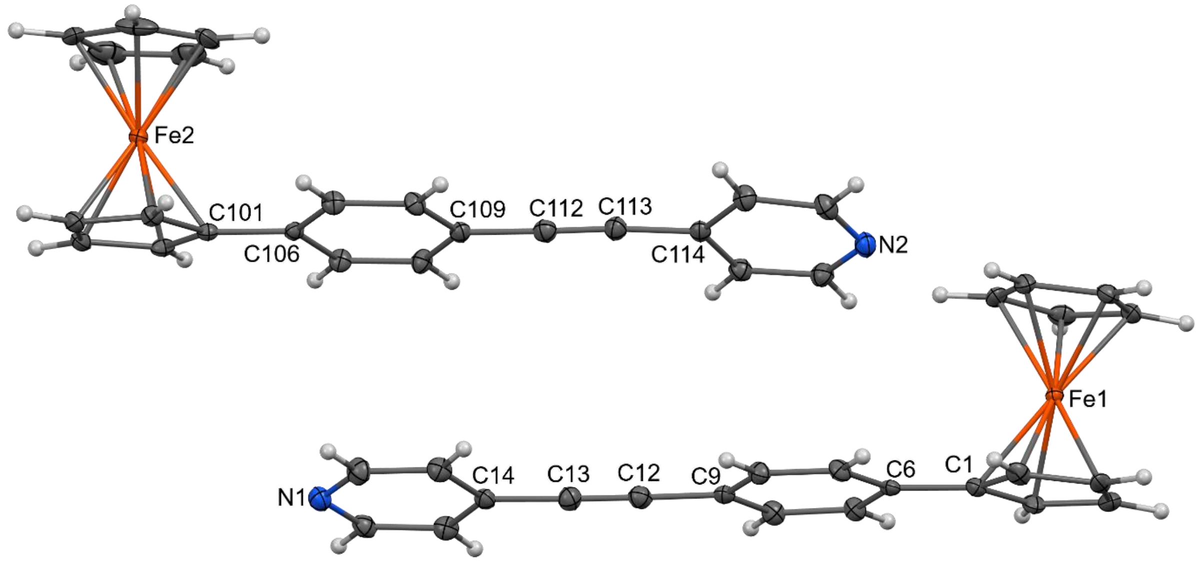

Figure S1. The molecular structure of **6a**, ORTEP view, 40% probability level. Two independent molecules are shown. Selected interatomic distances [Å]: C1—C6 1.469(4), C101—C106 1.474(4), C9—C12 1.435(4), C109—C112 1.439(4), C12—C13 1.195(4), C112—C113 1.199(4), C13—C14 1.441(4), C113—C114 1.435(4).

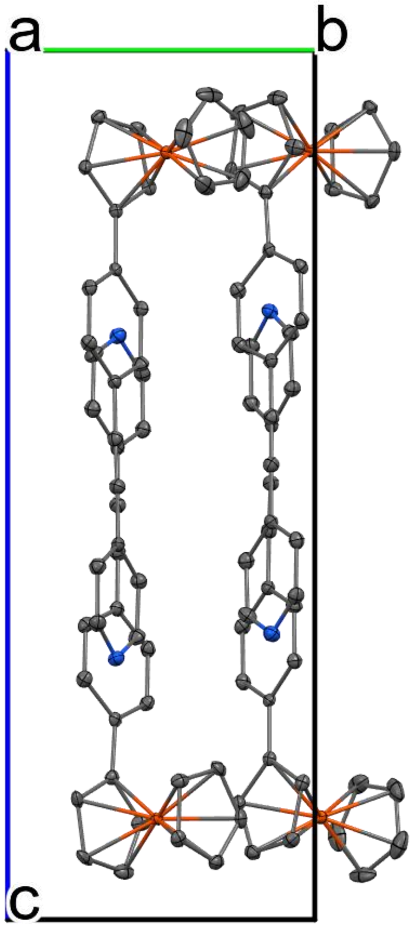

Figure S2. The supramolecular architecture of **6a**, view along *a*-axis.

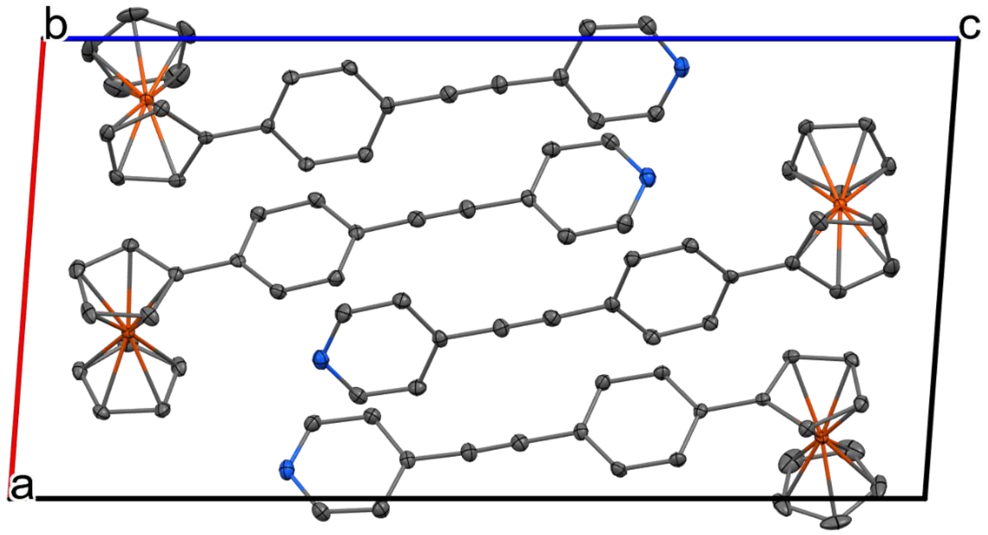

Figure S3. The supramolecular architecture of **6a**, view along *b*-axis.

Table S2. Experimental details for **7a**.

Crystal data	
Chemical formula	$C_{19}H_{15}FeNS$
M_r	345.23
Crystal system, space group	Orthorhombic, *Pbca*
Temperature (K)	150
a, *b*, *c* (Å)	10.7762 (9), 7.7571 (7), 35.821 (3)
V (Å^3)	2994.4 (4)
Z	8
Radiation type	Mo *K*α
μ (mm^{-1})	1.14
Crystal size (mm)	0.59 × 0.53 × 0.17
Data collection	
Diffractometer	Bruker D8 - Venture
Absorption correction	Multi-scan<br>*SADABS2016*/2 - Bruker AXS area detector scaling and absorption correction
T_{min}, T_{max}	0.441, 0.746
No. of measured, independent and observed [$I > 2\sigma(I)$] reflections	23270, 4603, 3508
R_{int}	0.051
$(\sin \theta/\lambda)_{max}$ (Å^{-1})	0.744
Refinement	
$R[F^2 > 2\sigma(F^2)]$, $wR(F^2)$, S	0.047, 0.094, 1.09
No. of reflections	4603
No. of parameters	199
No. of restraints	264
H-atom treatment	H-atom parameters constrained
$\Delta\rho_{max}$, $\Delta\rho_{min}$ (e Å^{-3})	0.38, −0.66

Computer programs: Bruker Instrument Service vV6.2.3, *APEX3* v2018.1-0 (Bruker AXS), *SAINT* V8.38A (Bruker AXS Inc., 2016), *SHELXT* 2014/5 (Sheldrick, 2014), *SHELXL2017*/1 (Sheldrick, 2017), Bruker *SHELXTL*.

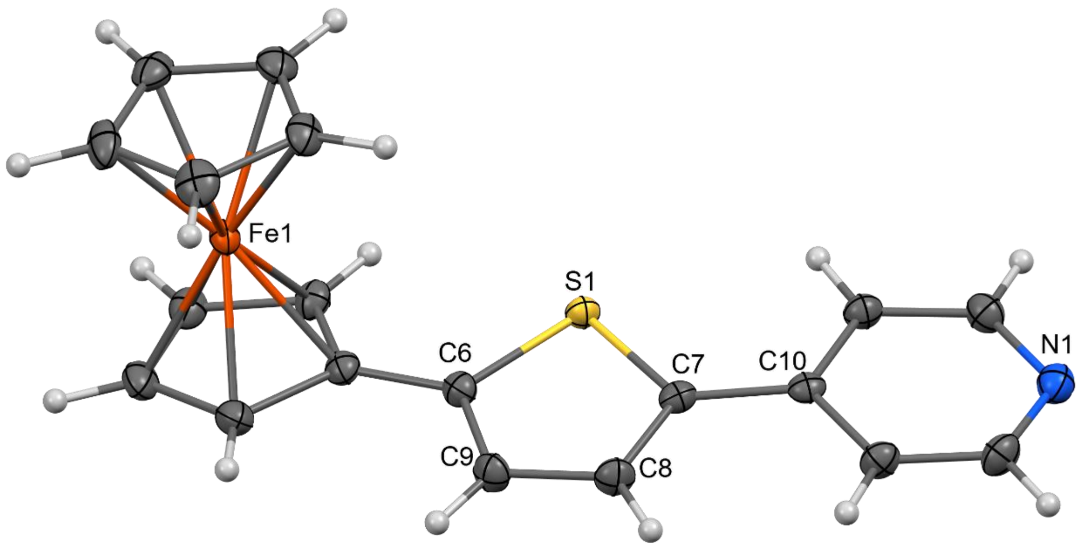

Figure S4. The molecular structure of **7a**, ORTEP view, 40% probability level. Selected interatomic distances [Å]: S1—C6 1.735(2), S1—C7 1.739(2), C7—C10 1.465(3), C1—C6 1.455(3), C8—C9 1.410(3).

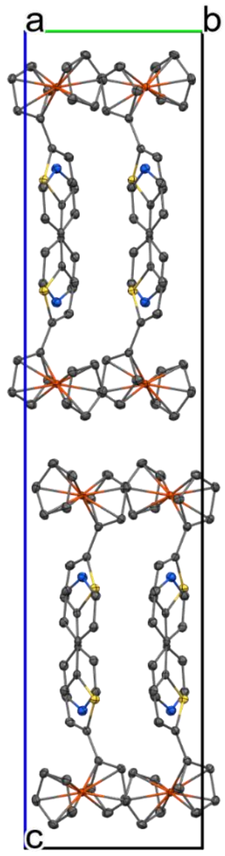

Figure S5. The supramolecular architecture of **7a**, view along *a*-axis

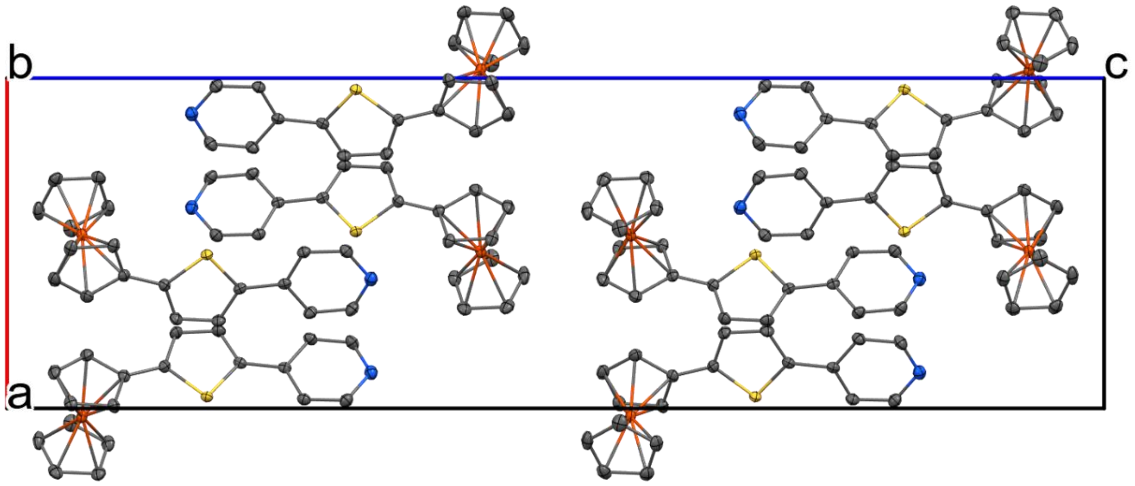

Figure S6. The supramolecular achritecture of **7a**, view along *b*-axis.

The X-ray data for colorless crystals of **6a** and **7a** were obtained at 150 K using Oxford Cryostream low-temperature device with a Bruker D8-Venture diffractometer equipped with Mo (Mo/K_α radiation; λ = 0.71073 Å) microfocus X-ray (IµS) source, Photon CMOS detector and Oxford Cryosystems cooling device was used for data collection. Obtained data were treated by XT-version 2014/5 and SHELXL-2017/1 software implemented in APEX3 v2016.9-0 (Bruker AXS) system.[3] $R_{int} = \sum|F_o^2 - F_{o,mean}^2|/\sum F_o^2$, $S = [\sum(w(F_o^2 - F_c^2)^2)/(N_{diffrs} - N_{params})]^{½}$ for all data, $R(F) = \sum||F_o| - |F_c||/\sum|F_o|$ for observed data, $wR(F^2) = [\sum(w(F_o^2 - F_c^2)^2)/(\sum w(F_o^2)^2)]^{½}$ for all data. Crystallographic data for all structural analysis have been deposited with the Cambridge Crystallographic Data Centre, CCDC nos. 2110542 and 2110543. Copies of this information may be obtained free of charge from The Director, CCDC, 12 Union Road, Cambridge CB2 1EY, UK (fax: +44-1223-336033; e-mail: deposit@ccdc.cam.ac.uk or www: http://www.ccdc.cam.ac.uk).

The frames for both complexes were integrated with the Bruker SAINT software package using a narrow-frame algorithm. Data were corrected for absorption effects using the Multi-Scan method (SADABS). The structures were solved and refined using the Bruker SHELXTL Software Package.

Hydrogen atoms were mostly localized on a difference Fourier map, however to ensure uniformity of treatment of crystal, all hydrogen were recalculated into idealized positions (riding model) and assigned temperature factors Hiso(H) = 1.2 Ueq (pivot atom). H atoms of C-H moieties employed in aromatic rings were placed with C-H distances of 0.93 Å. The structure of 7a has been solved as a two-component inversion twin.

[3] G. M. Sheldrick, *Acta Cryst. A* 2015, **71**, 3.

3. Electrochemistry

General method

The electrochemical behaviour of target chromophores were investigated in acetonitrile containing 0.1 M Bu_4NPF_6 in a three electrode cell by cyclic voltammetry (CV). The working electrode was glassy carbon disk (1 mm in diameter). As the reference and auxiliary electrodes were used leak-less Ag/AgCl electrode (SSCE) containing filling electrolyte (3.4 M KCl) and titanium rod with a thick coating of platinum, respectively. All peak potentials are given *vs*. SSCE. Voltammetric measurements were performed by using an integrated potentiostat system ER466 (eDAQ Europe, Warszawa, Poland) operated with EChem Electrochemistry software.

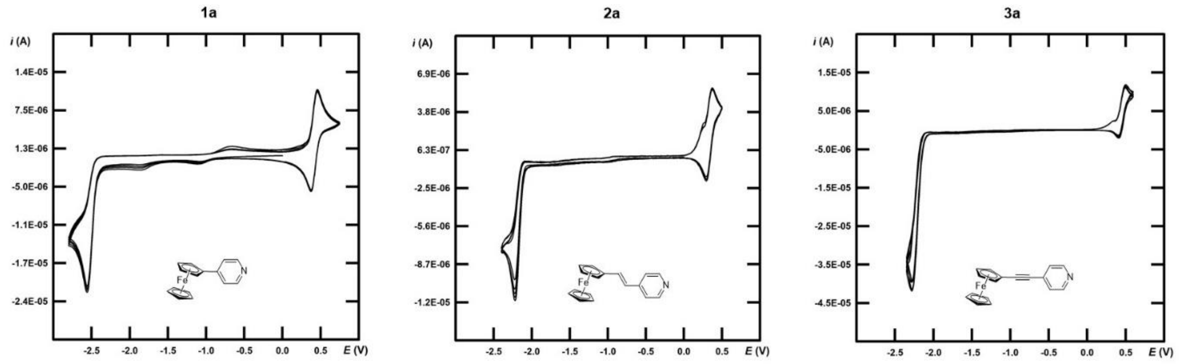

Figure S7. Cyclic voltammograms of chromophores **1a**–**3a** measured in acetonitrile containing 0.1 M Bu_4NPF_6 at glassy carbon electrode; v = 100 mV × s^{-1}.

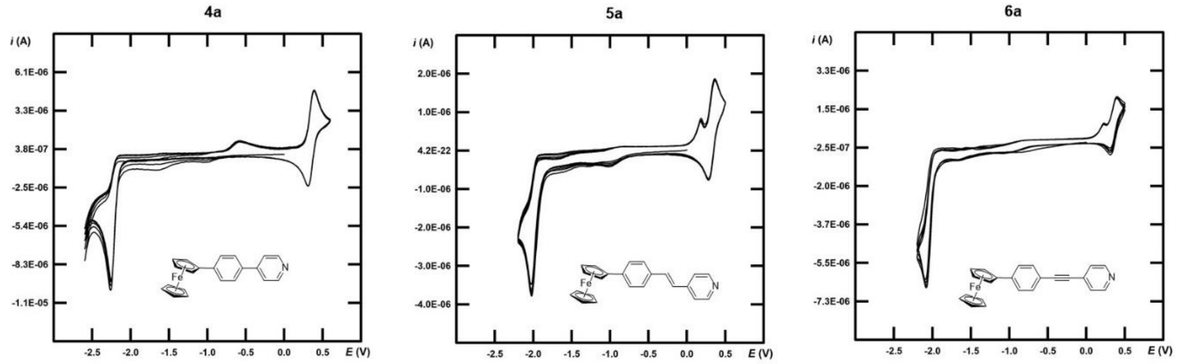

Figure S8. Cyclic voltammograms of chromophores **4a**–**6a** measured in acetonitrile containing 0.1 M Bu_4NPF_6 at glassy carbon electrode; v = 100 mV × s^{-1}.

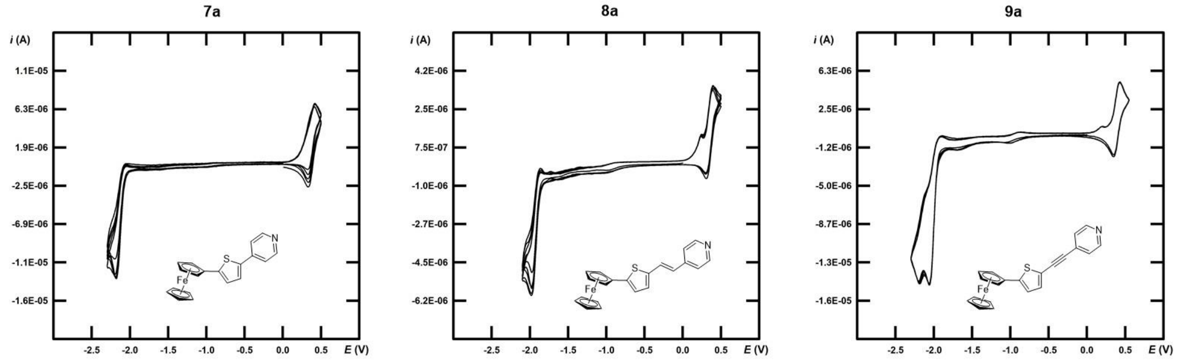

Figure S9. Cyclic voltammograms of chromophores **7a**–**9a** measured in acetonitrile containing 0.1 M Bu_4NPF_6 at glassy carbon electrode; v = 100 mV × s^{-1}.

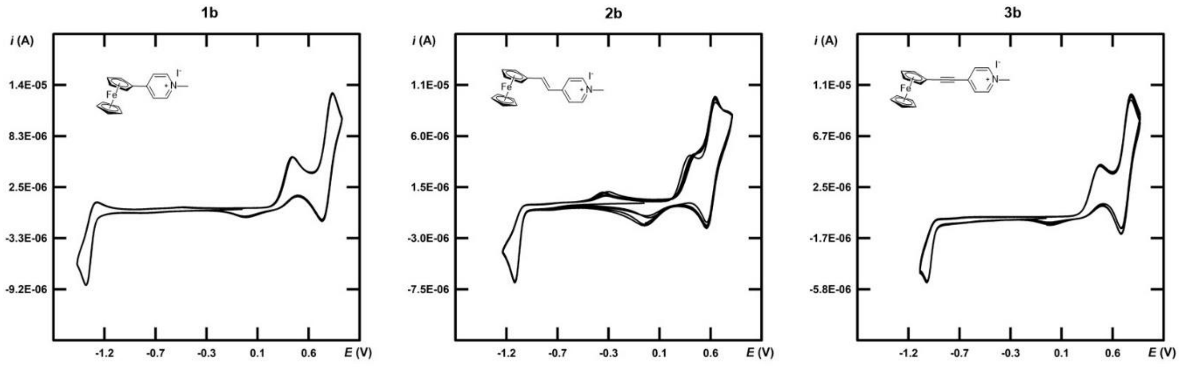

Figure S10. Cyclic voltammograms of chromophores **1b**–**3b** measured in acetonitrile containing 0.1 M Bu_4NPF_6 at glassy carbon electrode; v = 100 mV × s^{-1}.

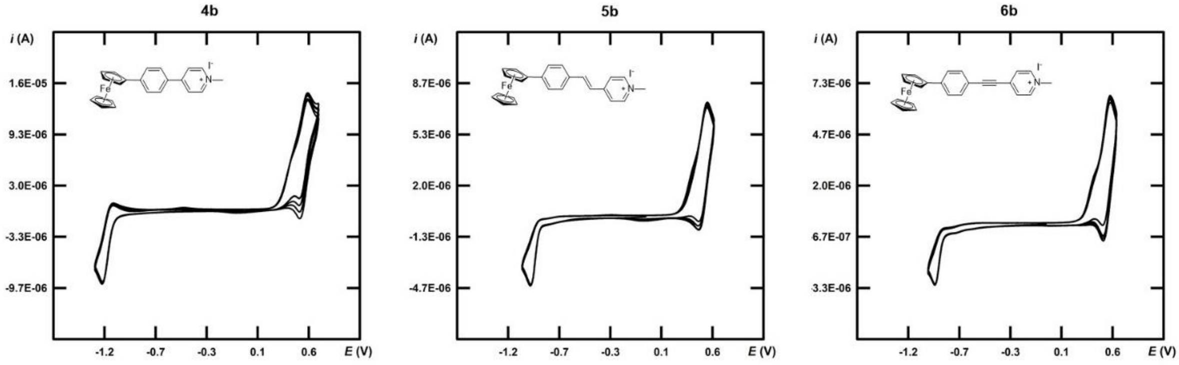

Figure S11. Cyclic voltammograms of chromophores **4b**–**6b** measured in acetonitrile containing 0.1 M Bu_4NPF_6 at glassy carbon electrode; v = 100 mV × s^{-1}.

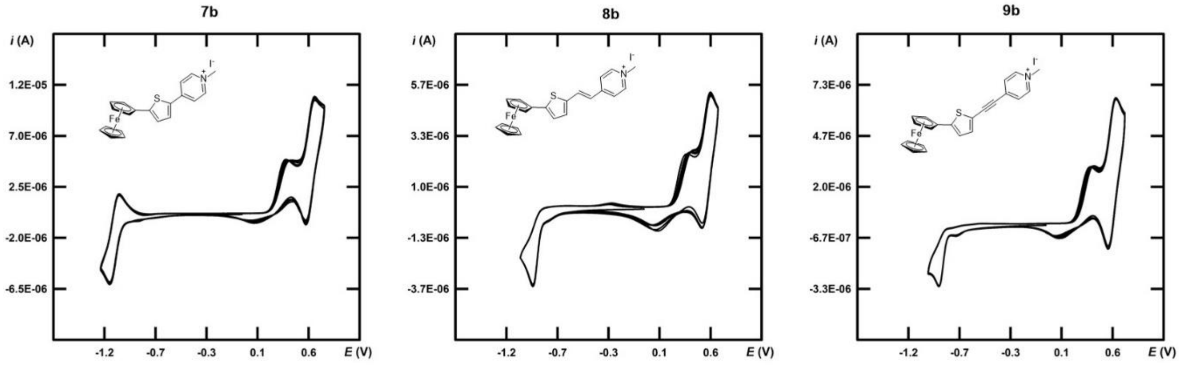

Figure S12. Cyclic voltammograms of chromophores **7b**–**9b** measured in acetonitrile containing 0.1 M Bu_4NPF_6 at glassy carbon electrode; v = 100 mV × s^{-1}.

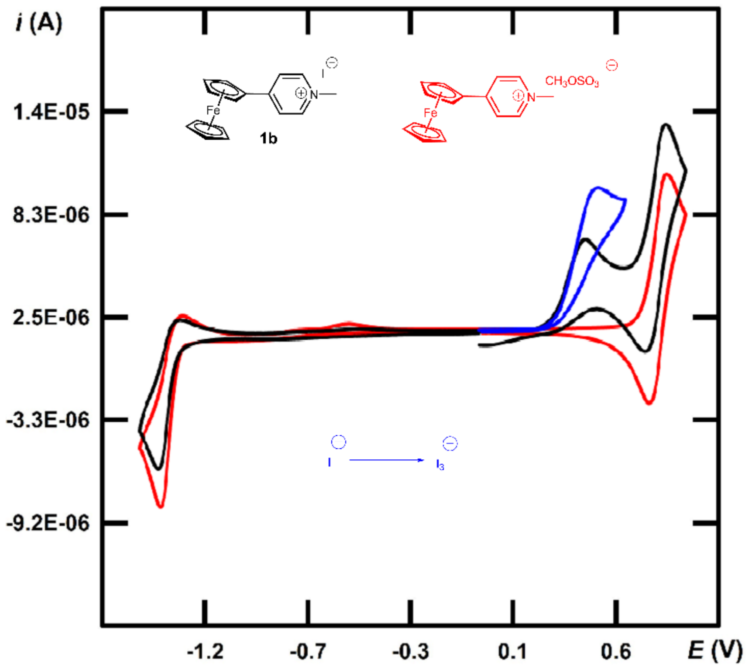

Figure S13. CV diagrams of **1b** (black) and its analogue with $MeOSO_3^-$ anion (red) along with the oxidation of I^- to I_3^- (blue).

4. Absorption spectra

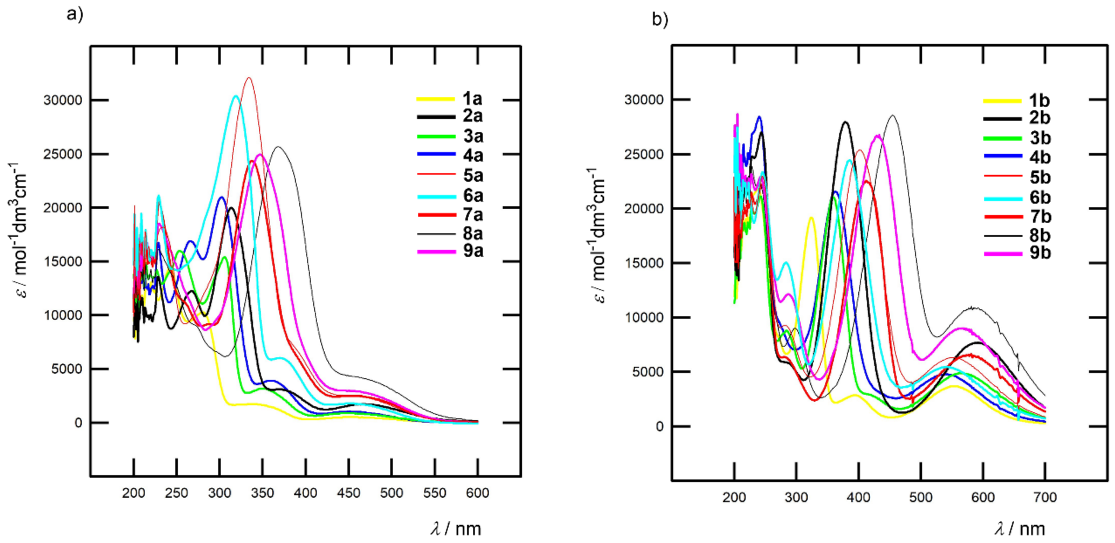

Figure S14. Electronic absorption spectra of **1a**–**9a** (a) and **1b**–**9b** (b) measured in DCM.

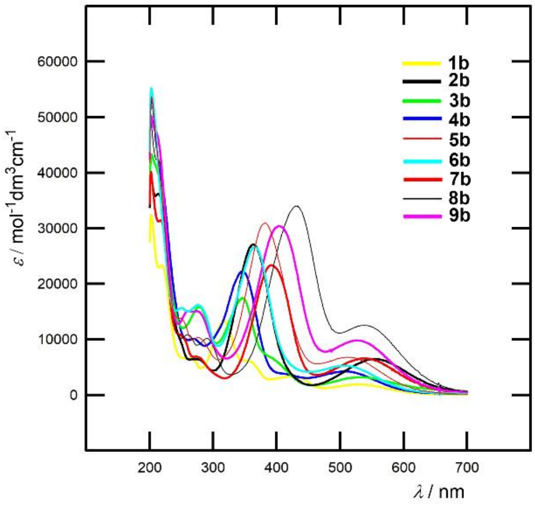

Figure S15. Electronic absorption spectra of **1b**–**9b** measured in CH_3OH.

Table S3. Optical properties of chromophores **1**–**9** measured in solvents of increased polarity.

	**DCM** (E_T^N = 0.309)		**ACN** (E_T^N = 0.460)		**MeOH** (E_T^N = 0.460)	
Comp.	λ_{max}^{HE} [nm (eV)] / ε (×10^3) [M^{-1} cm^{-1}]	λ_{max}^{LE} [nm (eV)] / ε (×10^3) [M^{-1} cm^{-1}]	λ_{max}^{HE} [nm (eV)] / ε (×10^3) [M^{-1} cm^{-1}]	λ_{max}^{LE} [nm (eV)] / ε (×10^3) [M^{-1} cm^{-1}]	λ_{max}^{HE} [nm (eV)] / ε (×10^3) [M^{-1} cm^{-1}]	λ_{max}^{LE} [nm (eV)] / ε (×10^3) [M^{-1} cm^{-1}]
**1a**	279 (4.44) / 10.3	466 (2.66) / 0.5	280 (4.43) / 10.5	456 (2.72) / 0.6	-	-
**2a**	313 (3.96) / 20.0	475 (2.61) / 1.7	312 (3.97) / 22.8	461 (2.69) / 1.9	-	-
**3a**	306 (4.05) /15.4	451 (2.75) / 0.9	303 (4.09) / 15.5	457 (2.71) / 1.1	-	-
**4a**	302 (4.11) / 21.0	452 (2.74) / 1.1	300 (4.13) / 23.3	460 (2.70) / 1.3	-	-
**5a**	334 (3.71) / 32.1	463 (2.68) / 2.4	331 (3.75) / 34.5	457 (2.71) / 2.5	-	-
**6a**	319 (3.89) / 30.4	457 (2.71) / 1.8	317 (3.91) / 32.7	460 (2.70) / 2.0	-	-
**7a**	338 (3.67) / 24.4	464 (2.67) / 2.5	336 (3.69) / 19.3	460 (2.70) / 2.0	-	-
**8a**	369 (3.36) / 25.7	471 (2.63) / 4.1	366 (3.39) / 29.2	462 (2.68) / 4.3	-	-
**9a**	348 (3.56) / 24.9	465 (2.67) / 2.8	345 (3.59) / 25.9	464 (2.67) / 3.0	-	-
**1b**	324 (3.83) / 19.2	556 (2.23) / 3.7	313 (3.96) / 15.9	525 (2.36) / 2.7	317 (3.91) / 13.3	534 (2.32) / 1.9
**2b**	378 (3.28) / 28.0	592 (2.09) / 7.7	361 (3.43) / 25.1	547 (2.27) / 6.3	363 (3.42) / 27.1	556 (2.23) / 6.4
**3b**	359 (3.45) / 21.1	566 (2.19) / 4.9	343 (3.62) / 20.1	531 (2.34) / 5.1	346 (3.58) / 17.5	532 (2.33) / 3.2
**4b**	364 (3.41) / 21.6	542 (2.29) / 4.8	344 (3.60) /22.2	501 (2.48) / 4.1	346 (3.58) / 22.3	508 (2.44) / 4.3
**5b**	402 (3.08) / 25.4	552 (2.25) / 6.3	378 (3.28) / 33.9	508 (2.44) / 7.2	382 (3.25) / 31.0	516 (2.40) / 6.8
**6b**	386 (3.21) / 24.5	542 (2.29) / 5.4	362 (3.43) / 29.7	504 (2.46) / 5.8	366 (3.39) / 26.6	506 (2.45) / 5.2
**7b**	412 (3.01) / 22.6	580 (2.14) / 6.5	390 (3.18) / 22.6	537 (2.31) / 6.2	392 (3.16) / 23.4	541 (2.29) / 6.5
**8b**	455 (2.73) / 28.6	585 (2.12) / 11.0	426 (2.91) / 32.3	532 (2.33) / 11.6	432 (2.87) / 34.0	537 (2.31) / 12.6
**9b**	433 (2.86) / 26.8	567 (2.19) / 9.0	402 (3.08) / 24.7	523 (2.37) / 8.0	404 (3.07) / 30.4	527 (2.35) / 9.8

5. DFT calculations

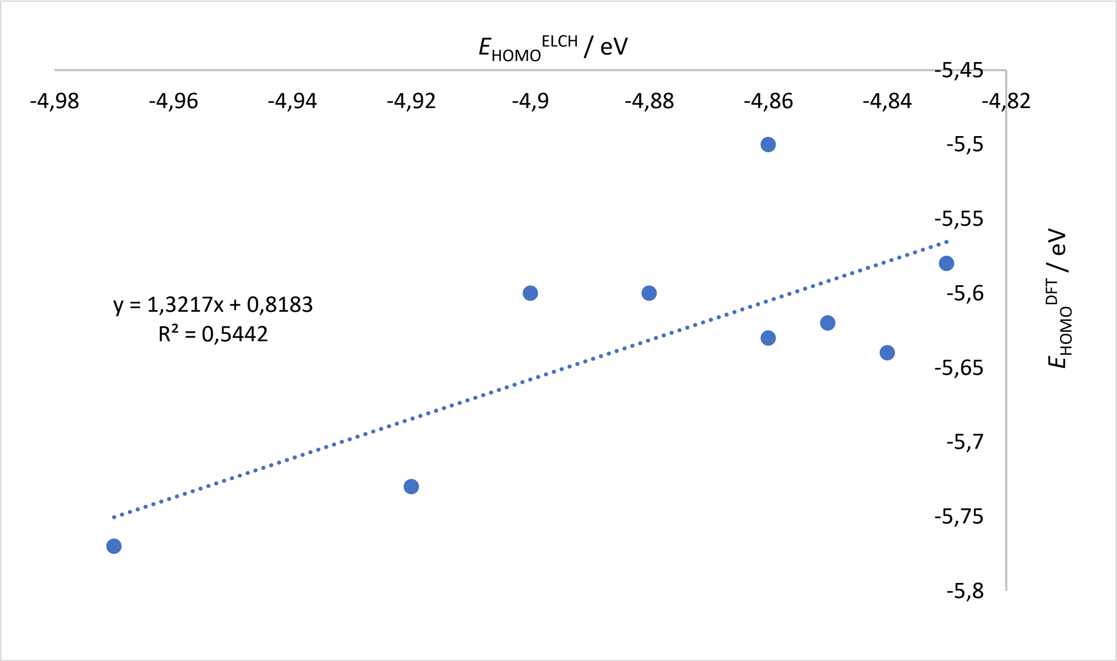

Figure S16. A correlation of the experimental (ELCH) and DFT-calculated HOMO energies of chromophores **1a**–**9a**.

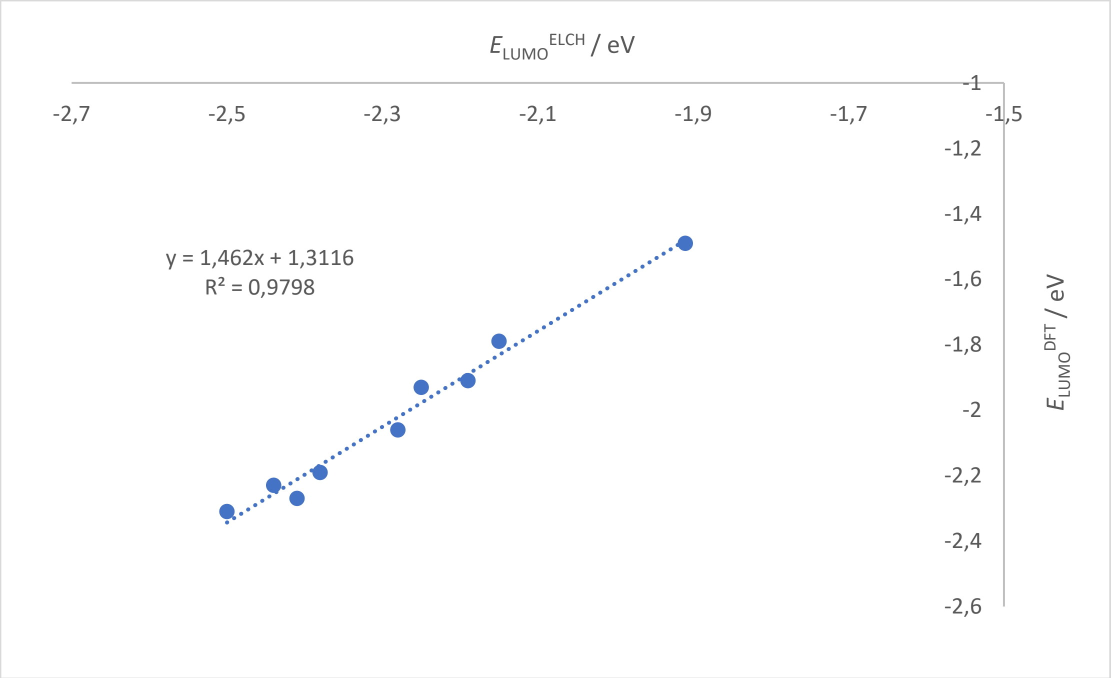

Figure S17. A correlation of the experimental (ELCH) and DFT-calculated LUMO energies of chromophores **1a**–**9a**.

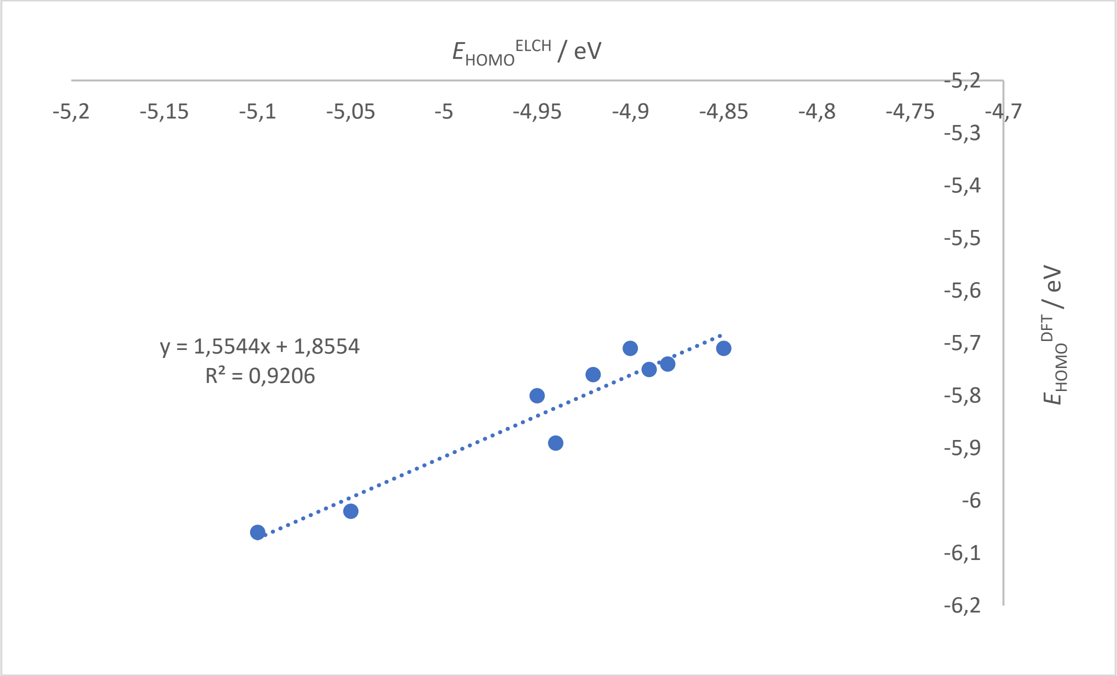

Figure S18. A correlation of the experimental (ELCH) and DFT-calculated HOMO energies of chromophores **1b**–**9b**.

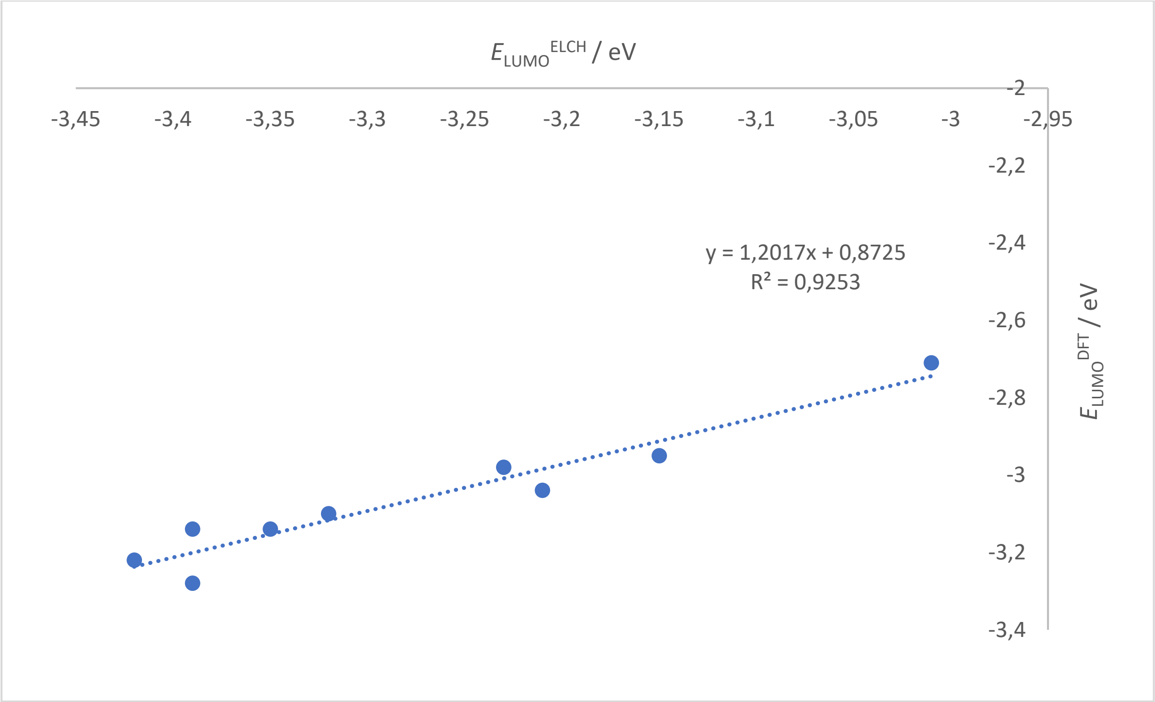

Figure S19. A correlation of the experimental (ELCH) and DFT-calculated LUMO energies of chromophores **1b**–**9b**.

The following localization of the frontier molecular orbitals were gained in the program OPChem.[4]

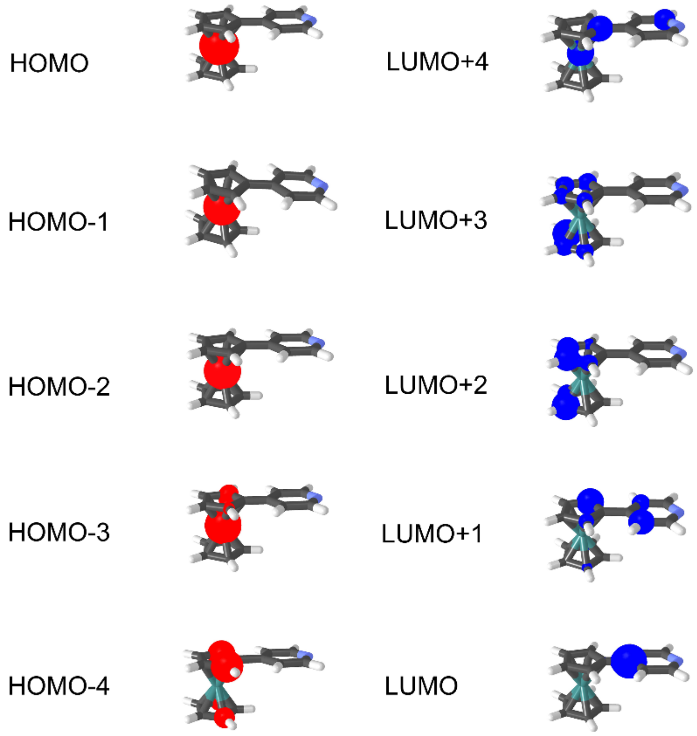

Figure S20. The HOMO (red) and the LUMO (blue) localizations in chromophore **1a**.

[4] O. Pytela, OPChem, Version 8.7. https://bures.upce.cz/OPgm/.

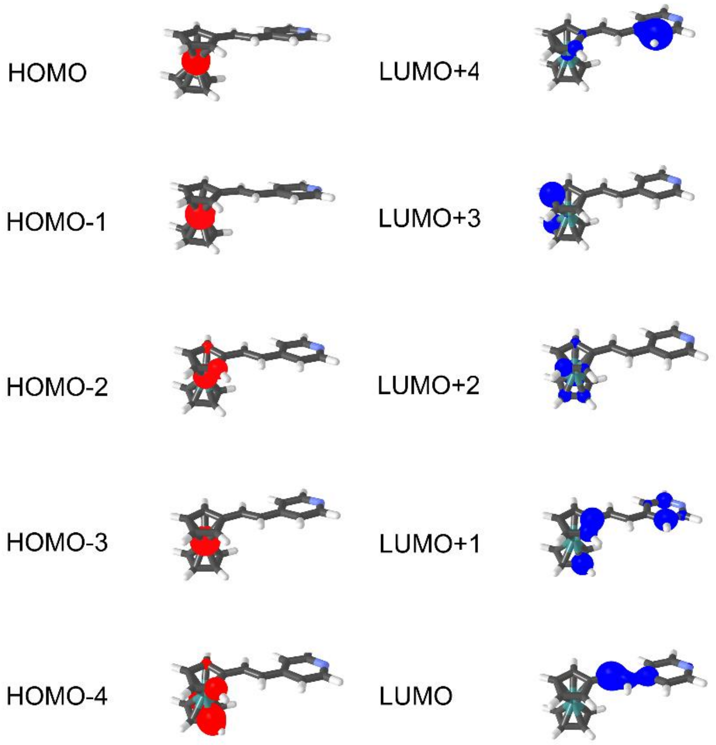

Figure S21. The HOMO (red) and the LUMO (blue) localizations in chromophore **2a**.

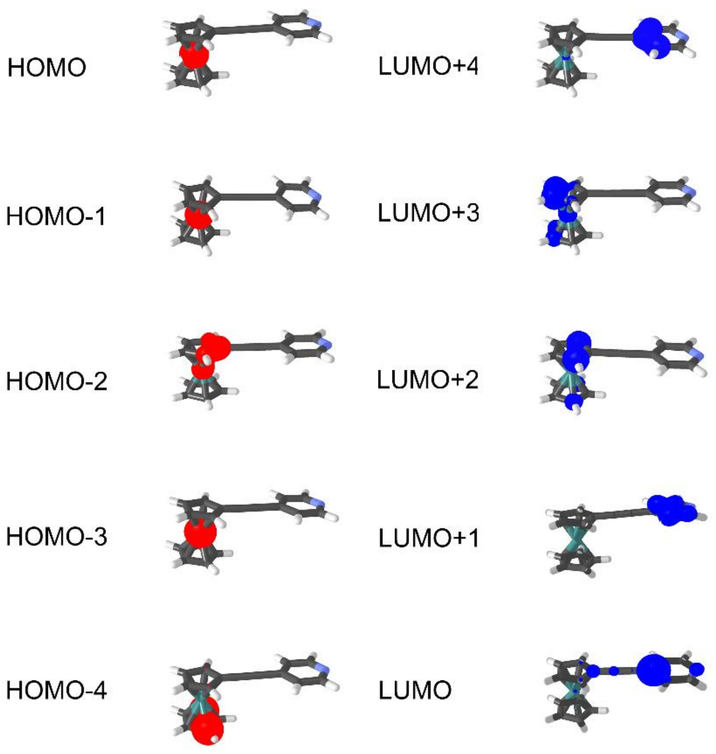

Figure S22. The HOMO (red) and the LUMO (blue) localizations in chromophore **3a**.

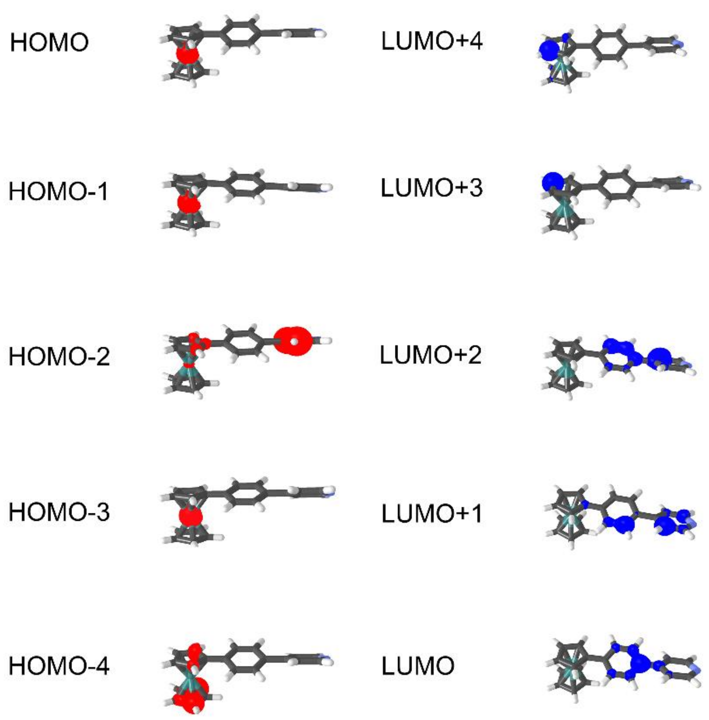

Figure S23. The HOMO (red) and the LUMO (blue) localizations in chromophore **4a**.

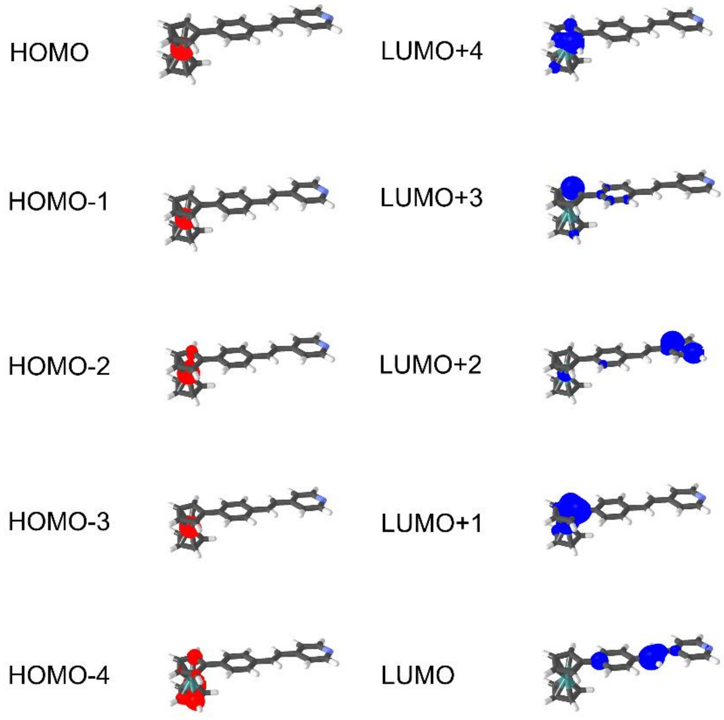

Figure S24. The HOMO (red) and the LUMO (blue) localizations in chromophore **5a**.

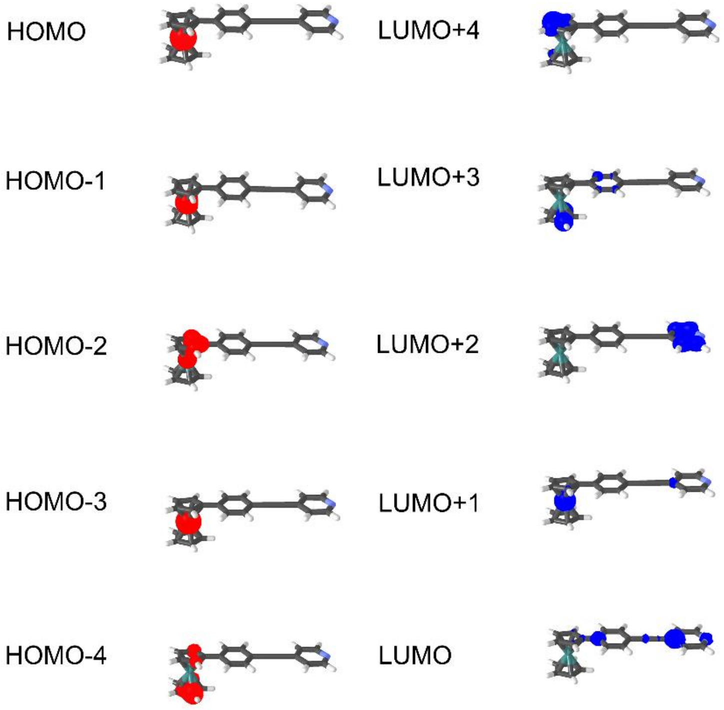

Figure S25. The HOMO (red) and the LUMO (blue) localizations in chromophore **6a**.

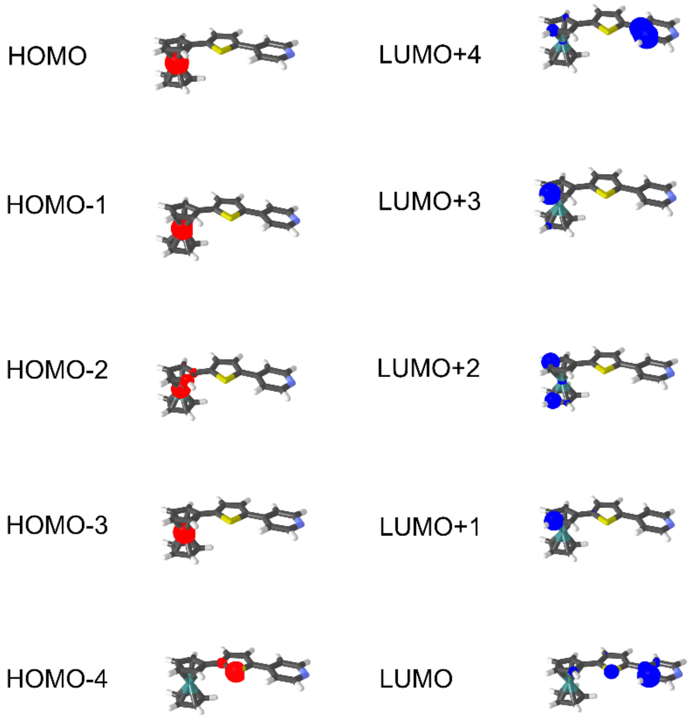

Figure S26. The HOMO (red) and the LUMO (blue) localizations in chromophore **7a**.

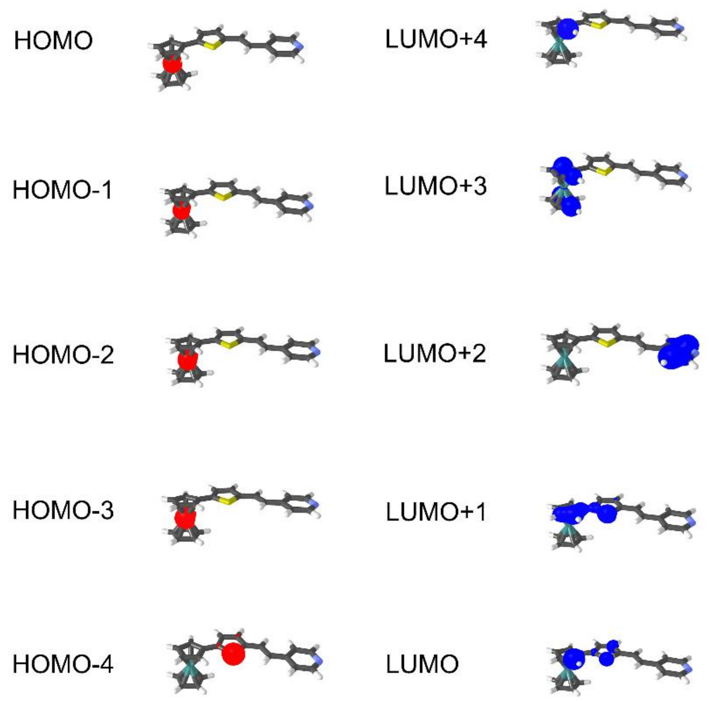

Figure S27. The HOMO (red) and the LUMO (blue) localizations in chromophore **8a**.

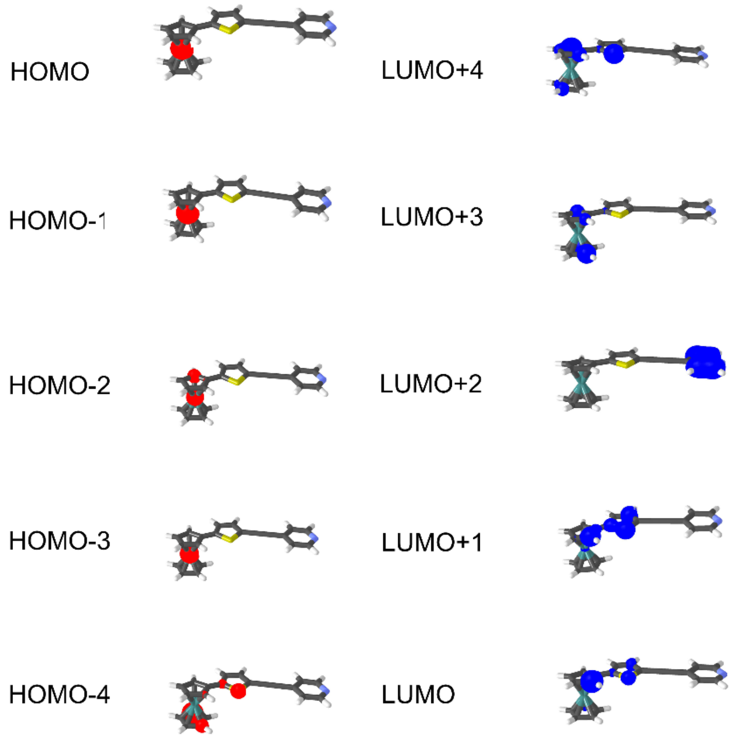

Figure S28. The HOMO (red) and the LUMO (blue) localizations in chromophore **9a**.

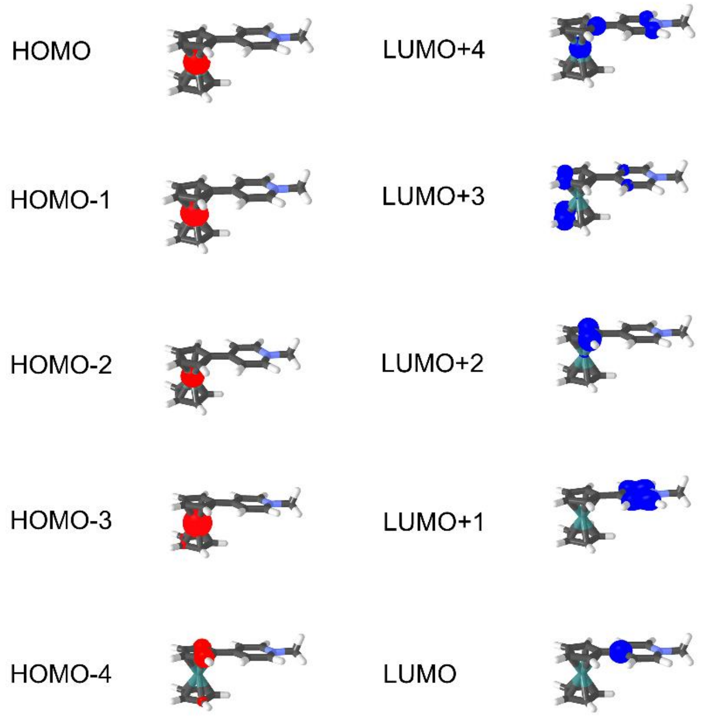

Figure S29. The HOMO (red) and the LUMO (blue) localizations in chromophore **1b** (the I^- anion has been omitted for clarity).

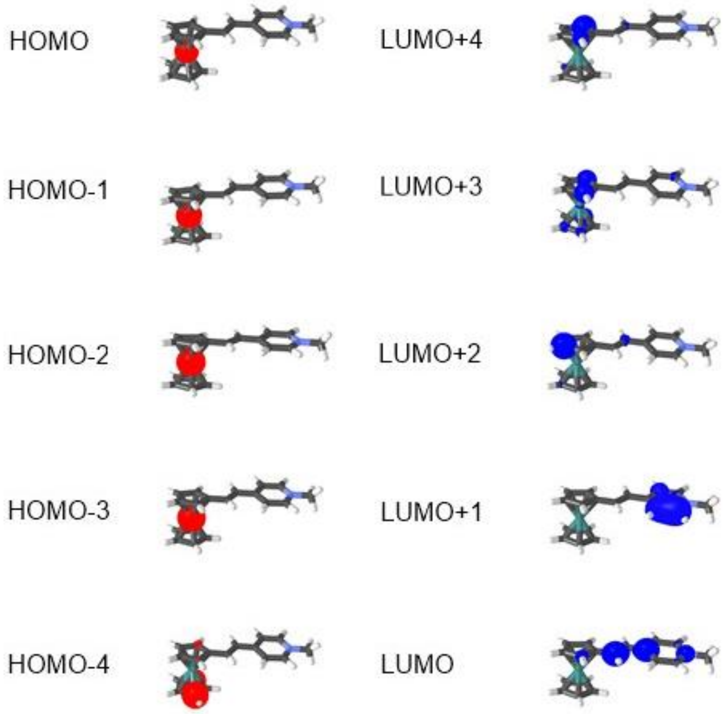

Figure S30. The HOMO (red) and the LUMO (blue) localizations in chromophore **2b** (the I^- anion has been omitted for clarity).

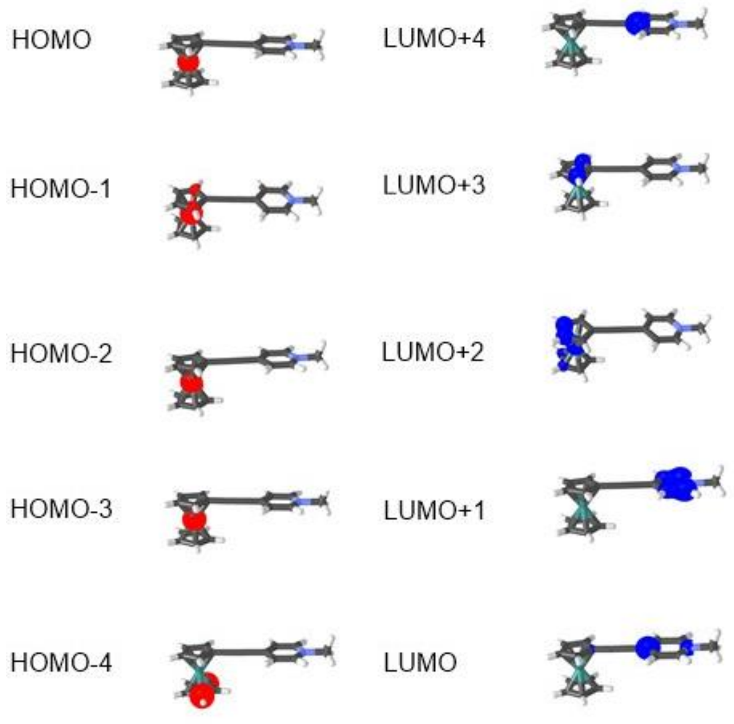

Figure S31. The HOMO (red) and the LUMO (blue) localizations in chromophore **3b** (the I^- anion has been omitted for clarity).

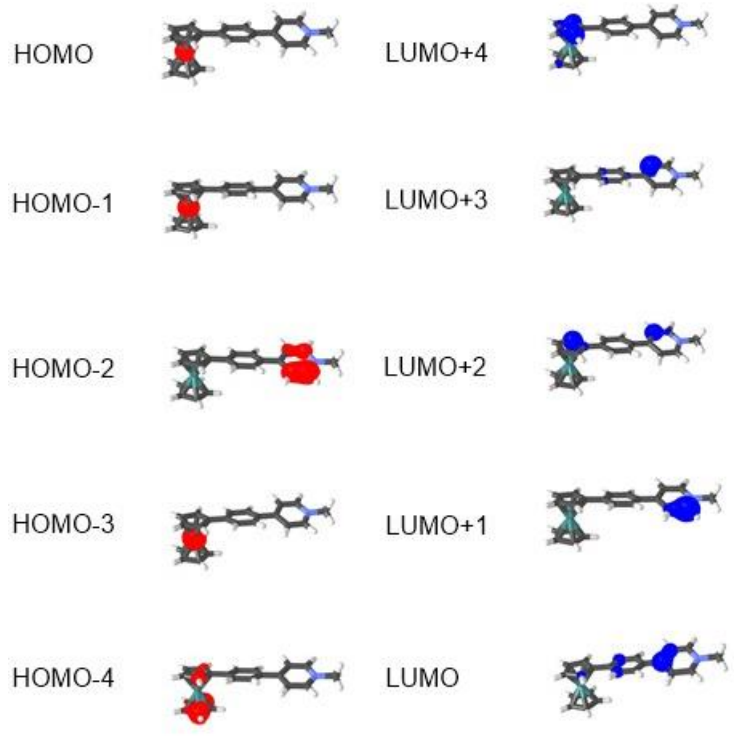

Figure S32. The HOMO (red) and the LUMO (blue) localizations in chromophore **4b** (the I^- anion has been omitted for clarity).

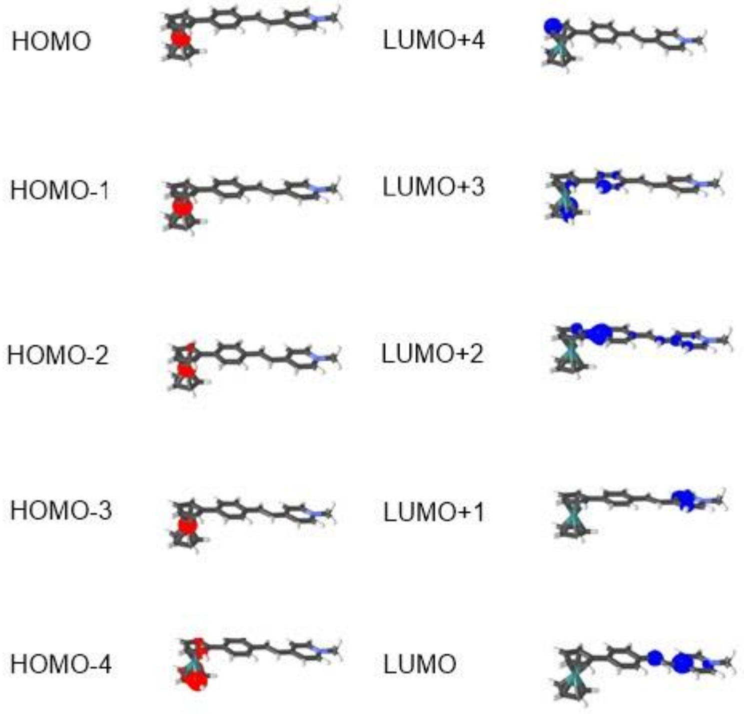

Figure S33. The HOMO (red) and the LUMO (blue) localizations in chromophore **5b** (the I$^-$ anion has been omitted for clarity).

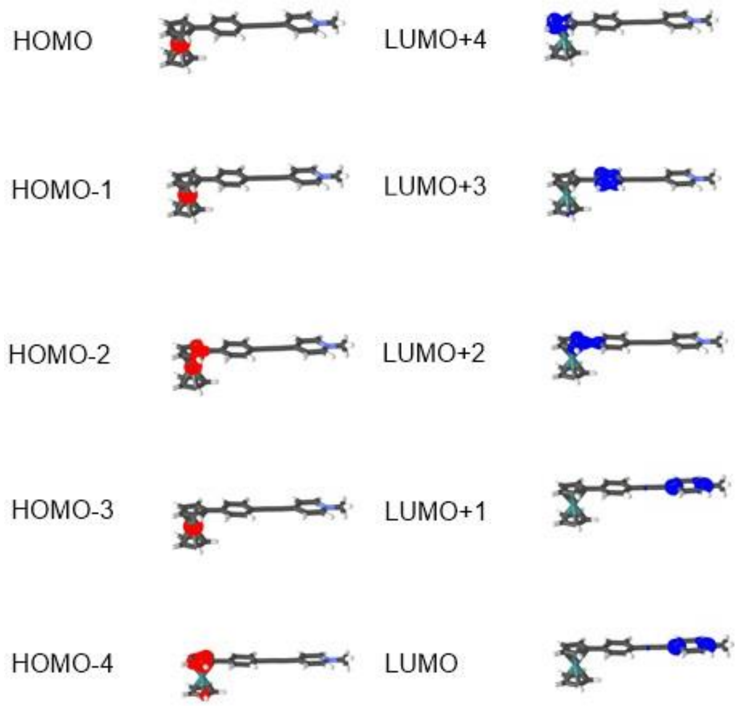

Figure S34. The HOMO (red) and the LUMO (blue) localizations in chromophore **6b** (the I$^-$ anion has been omitted for clarity).

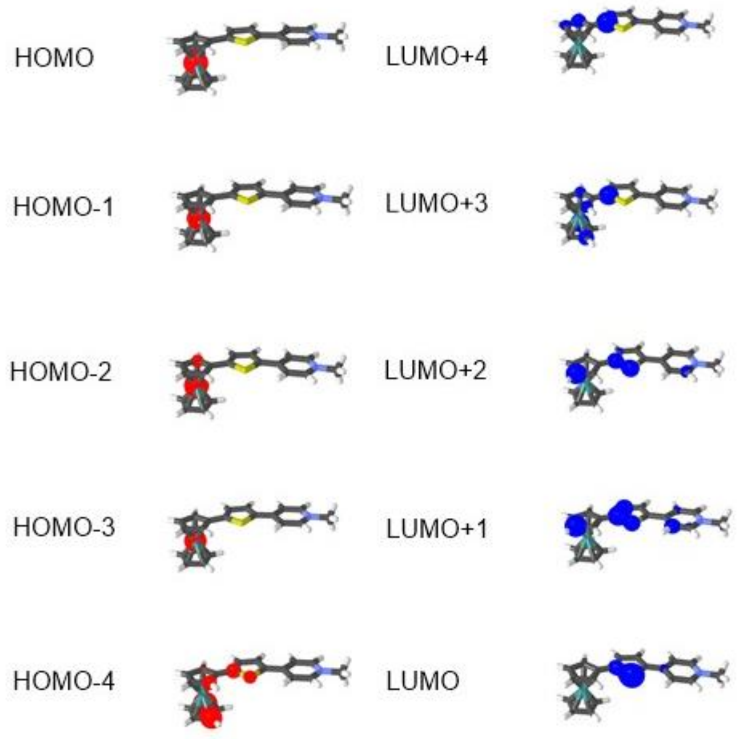

Figure S35. The HOMO (red) and the LUMO (blue) localizations in chromophore **7b** (the I^- anion has been omitted for clarity).

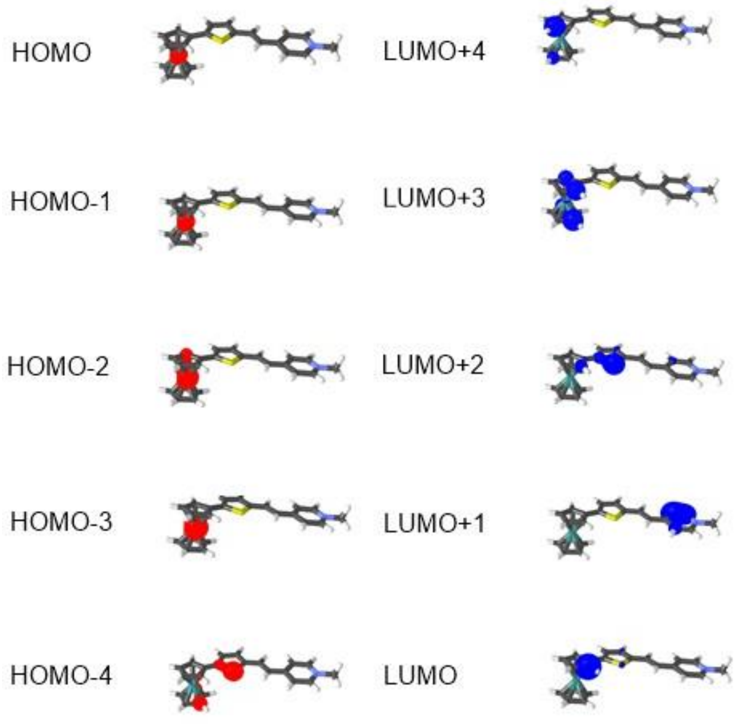

Figure S36. The HOMO (red) and the LUMO (blue) localizations in chromophore **8b** (the I^- anion has been omitted for clarity).

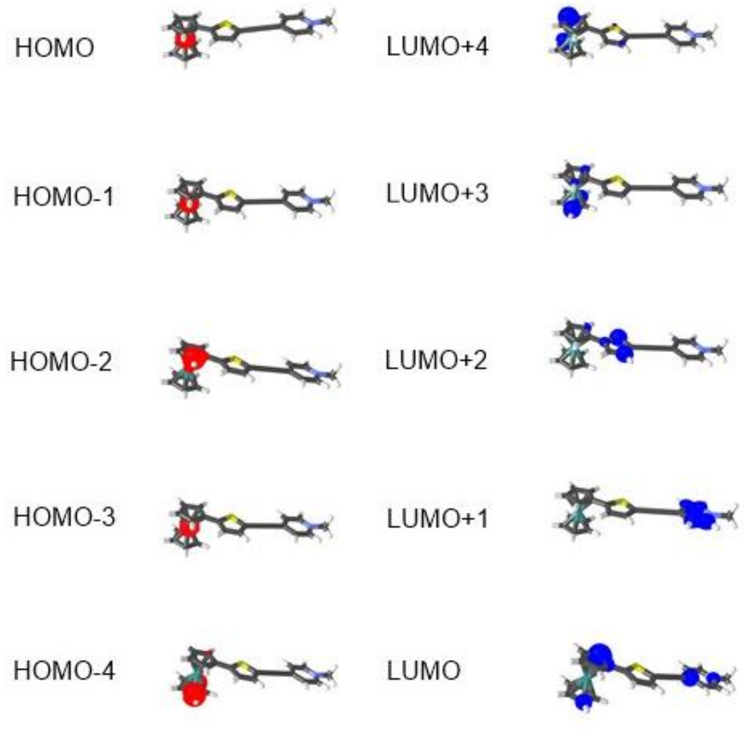

Figure S37. The HOMO (red) and the LUMO (blue) localizations in chromophore **9b** (the I^- anion has been omitted for clarity).

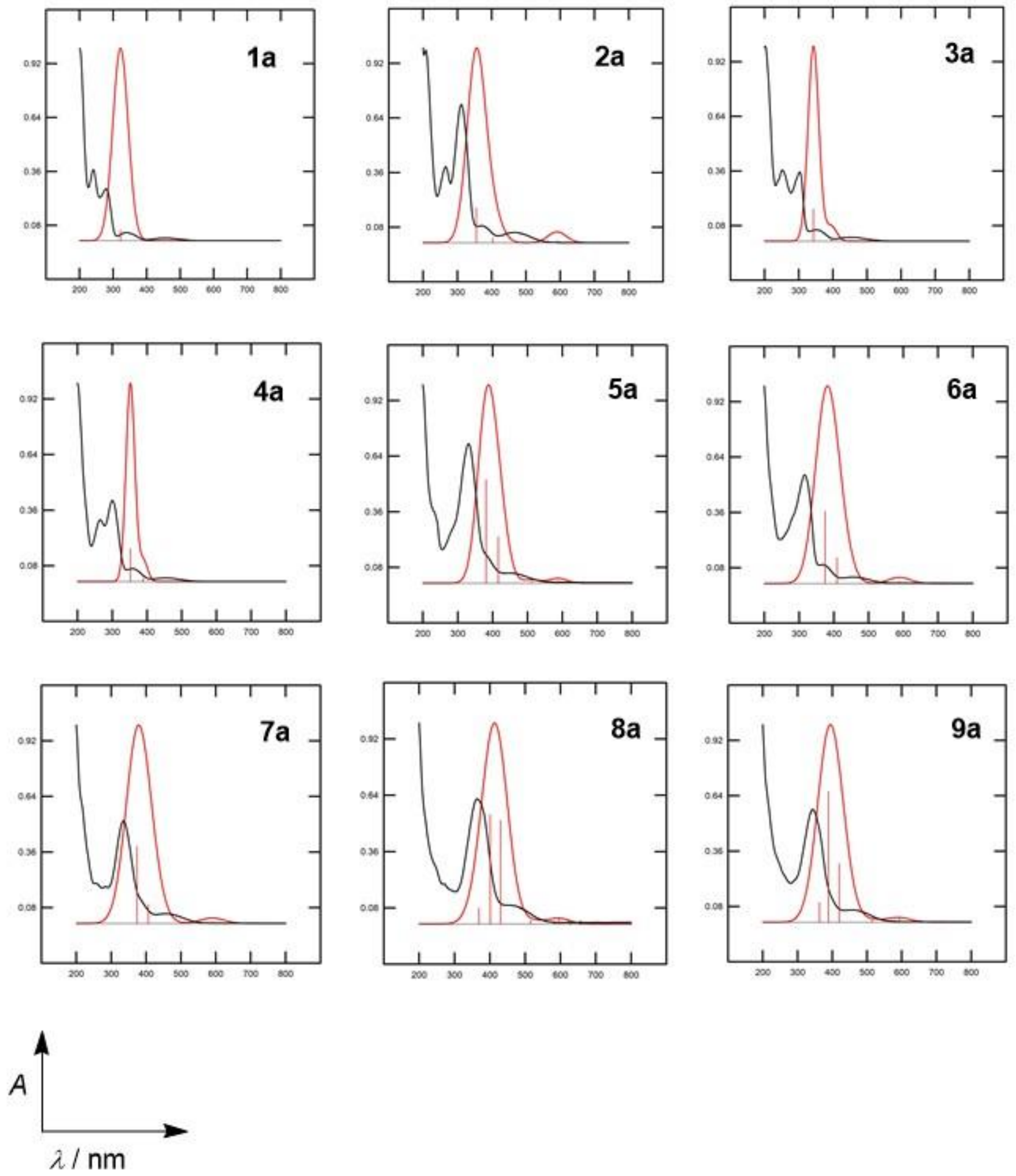

Figure S38. TD-DFT (n_{states} = 8) B3LYP/6-311+G(2d,f,p) calculated UV-Vis spectra of Fc-π-Py chromophores **1a**–**9a** in AN. Red vertical lines represent oscillator strengths (*f*).

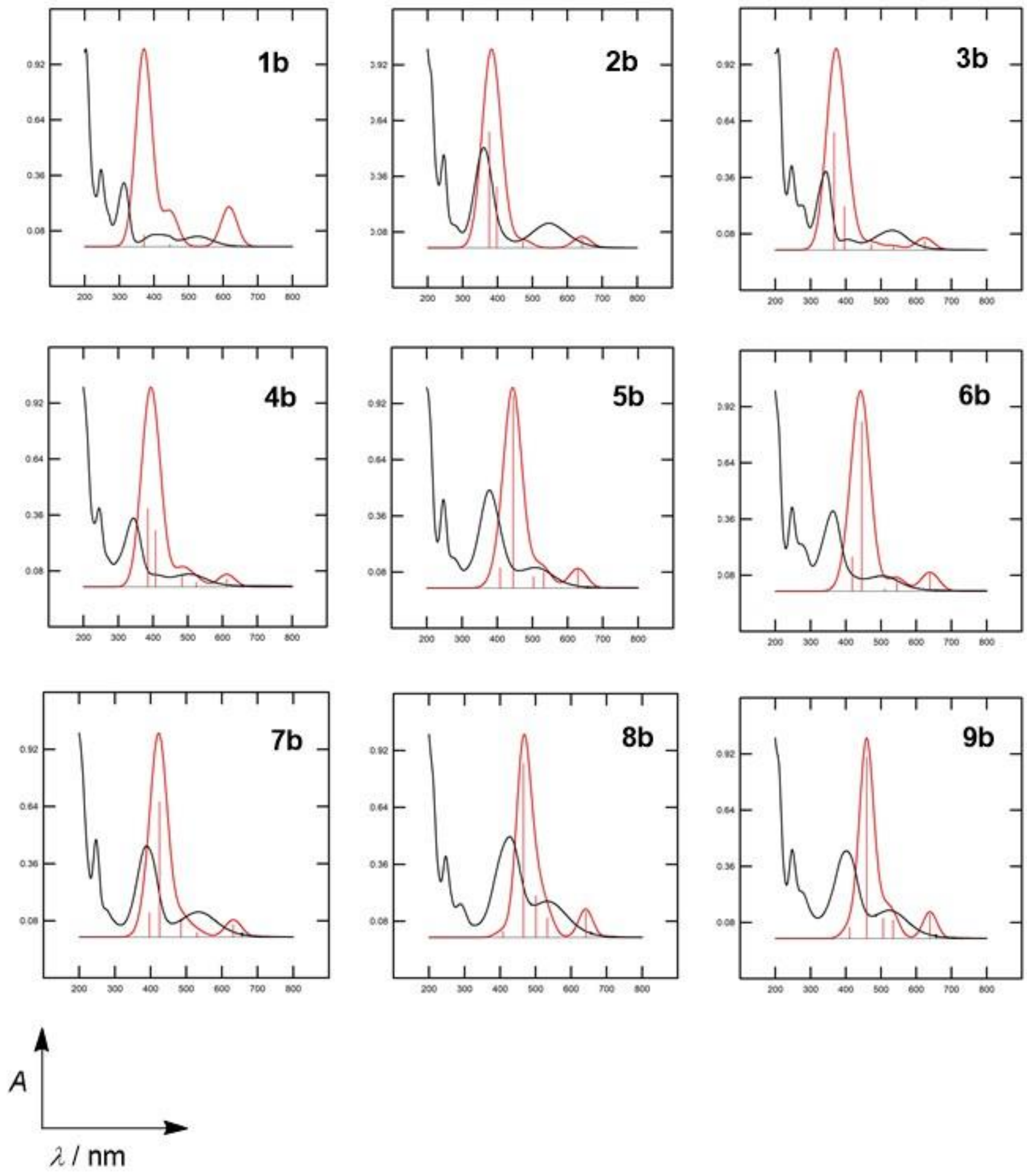

Figure S39. TD-DFT (n_{states} = 8) B3LYP/6-311+G(2d,f,p) calculated UV-Vis spectra of Fc-π-Py^{+} chromophores **1b**–**9b** in AN. Red vertical lines represent oscillator strengths (*f*).

6. NMR spectra

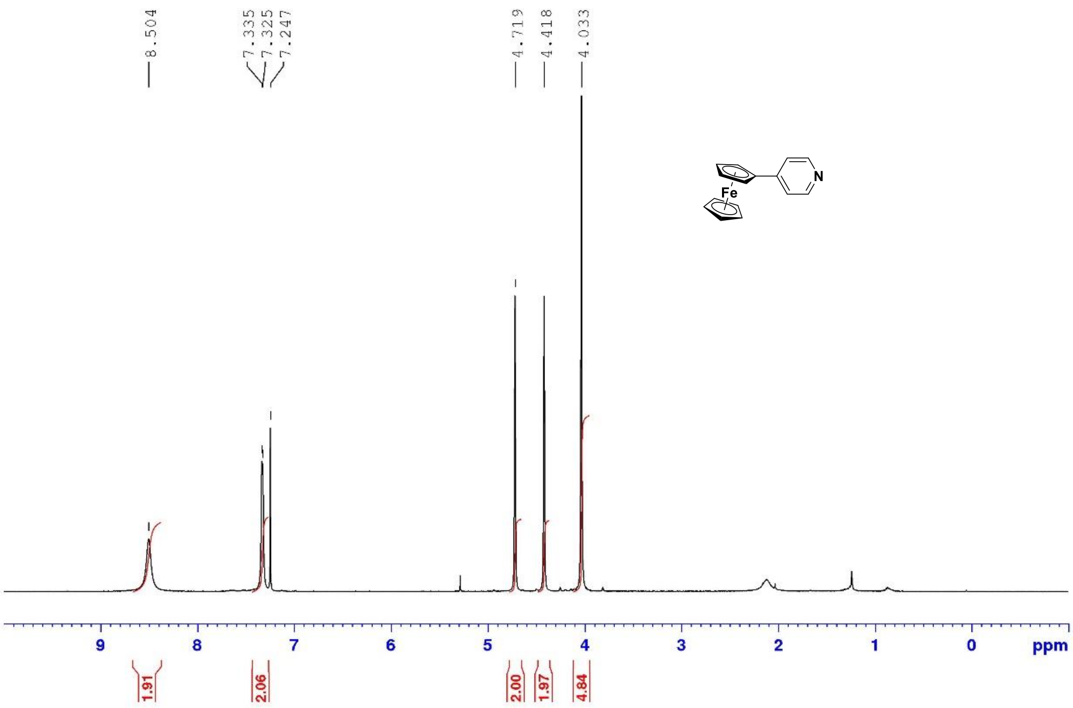

Figure S40. ^{1}H NMR spectrum of chromophore **1a** (400 MHz, $CDCl_3$, 25 °C).

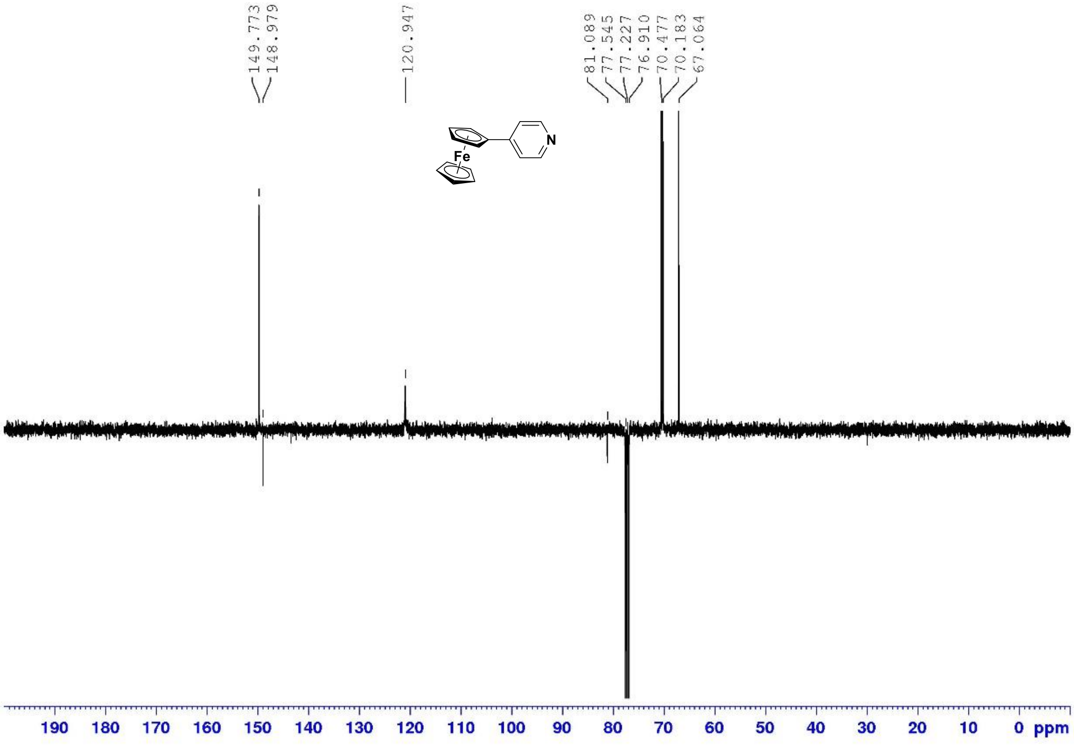

Figure S41. ^{13}C NMR APT spectrum of chromophore **1a** (100 MHz, $CDCl_3$, 25 °C).

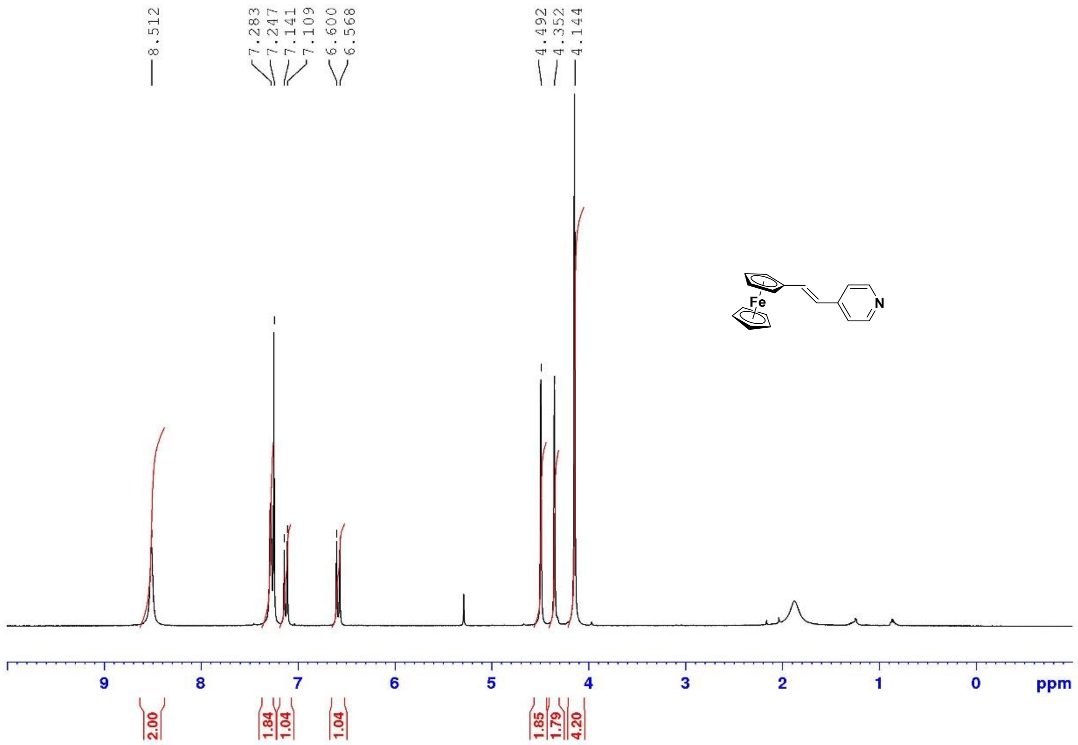

Figure S42. ^{1}H NMR spectrum of chromophore **2a** (500 MHz, $CDCl_3$, 25 °C).

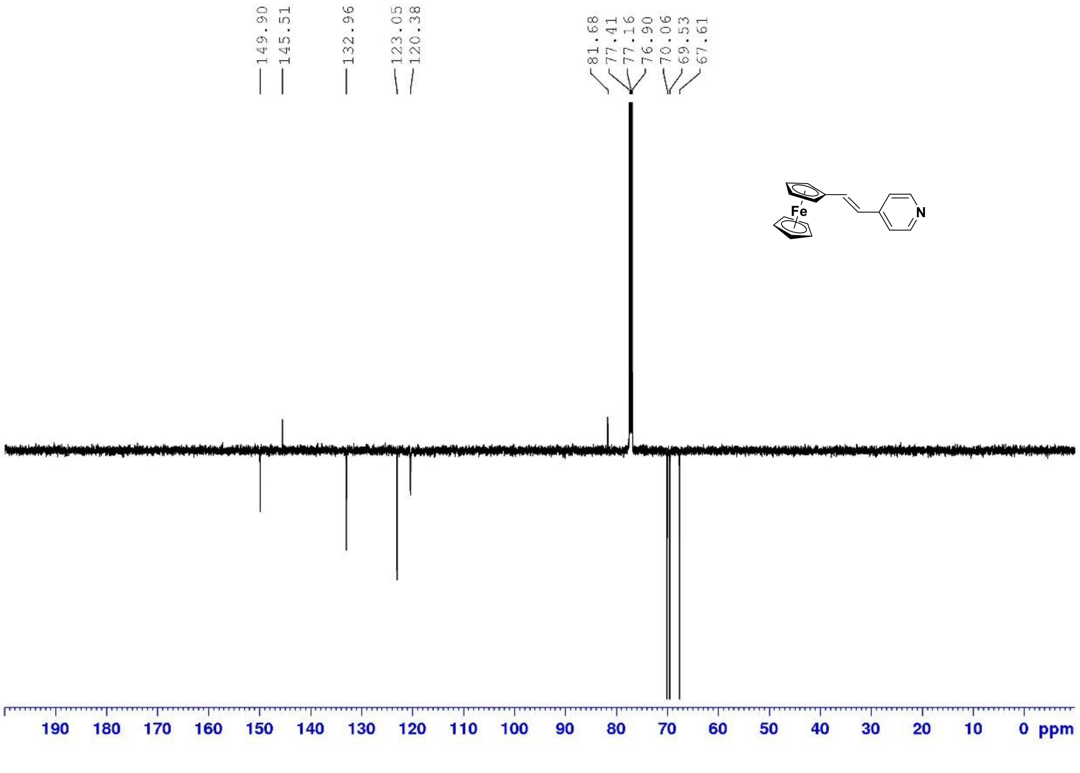

Figure S43. ^{13}C NMR APT spectrum of chromophore **2a** (125 MHz, $CDCl_3$, 25 °C).

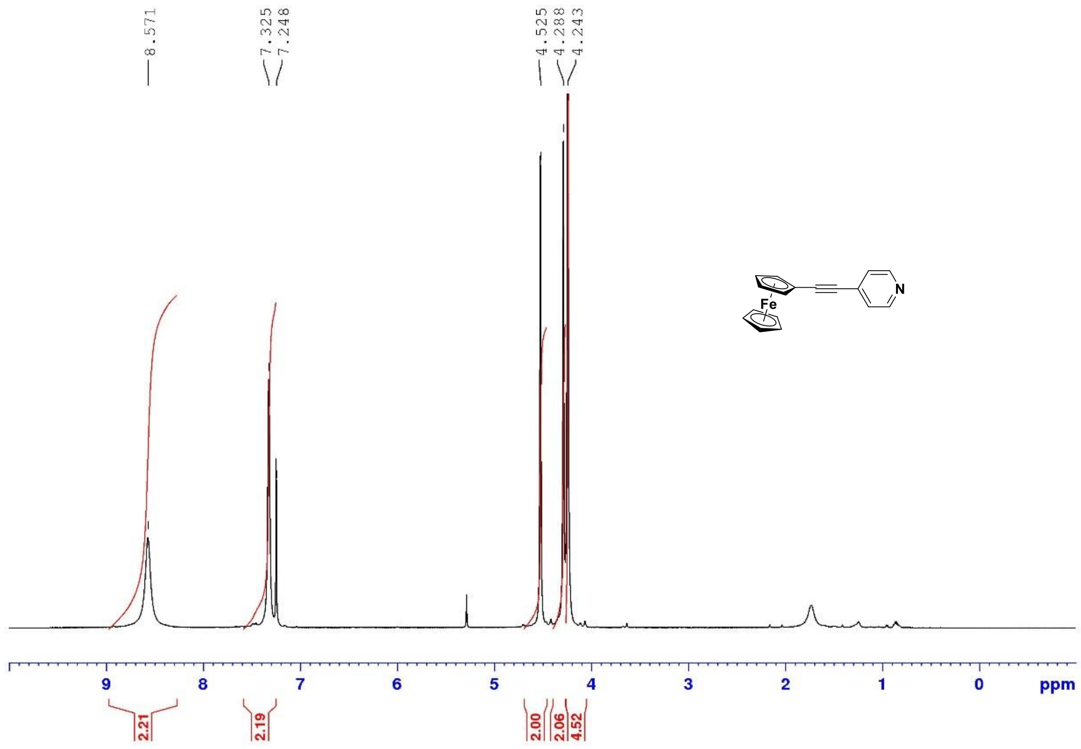

Figure S44. ^{1}H NMR spectrum of chromophore **3a** (500 MHz, $CDCl_3$, 25 °C).

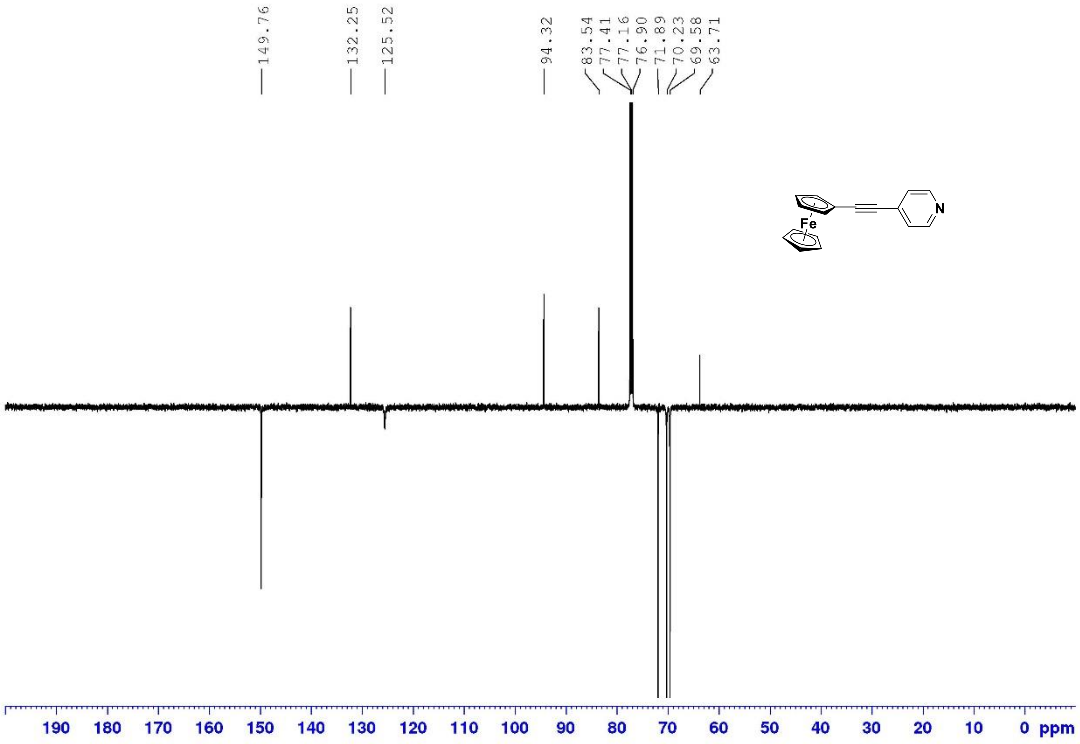

Figure S45. ^{13}C NMR APT spectrum of chromophore **3a** (125 MHz, $CDCl_3$, 25 °C).

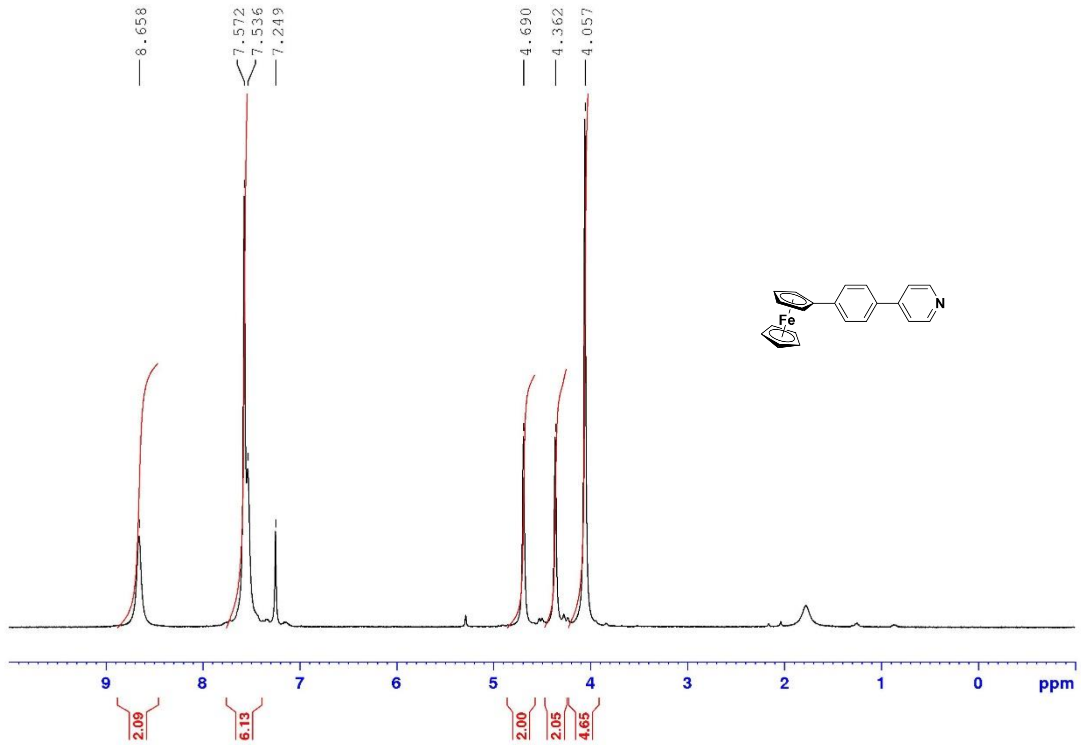

Figure S46. ^{1}H NMR spectrum of chromophore **4a** (400 MHz, $CDCl_3$, 25 °C).

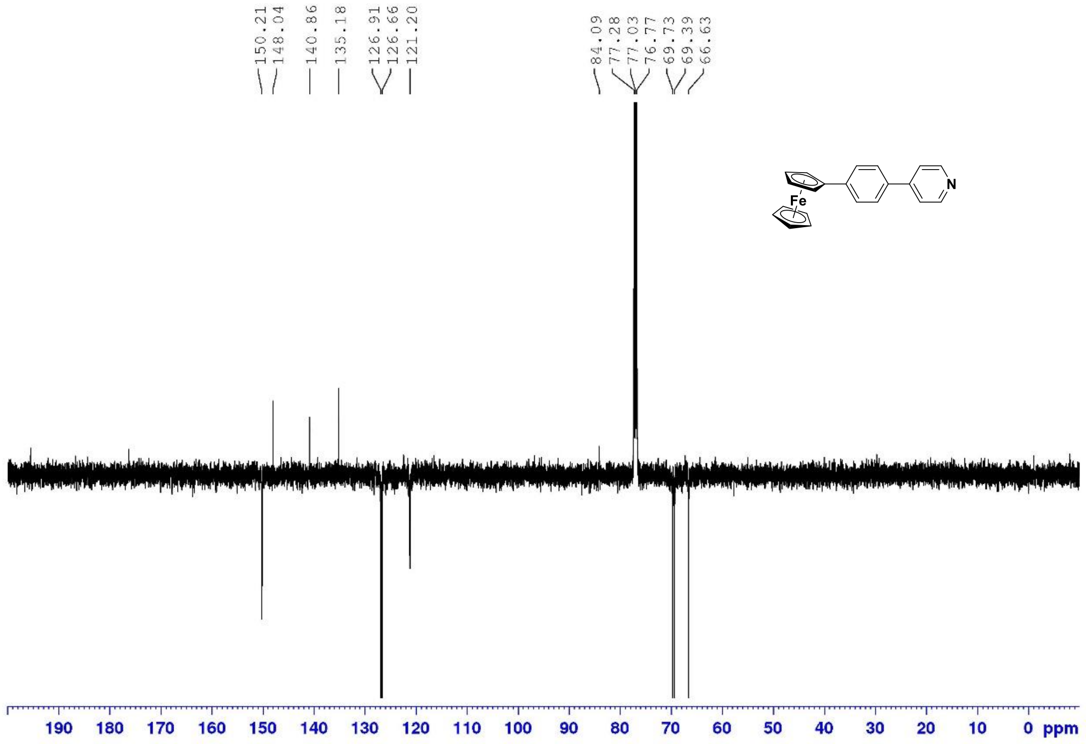

Figure S47. ^{13}C NMR APT spectrum of chromophore **4a** (125 MHz, $CDCl_3$, 25 °C).

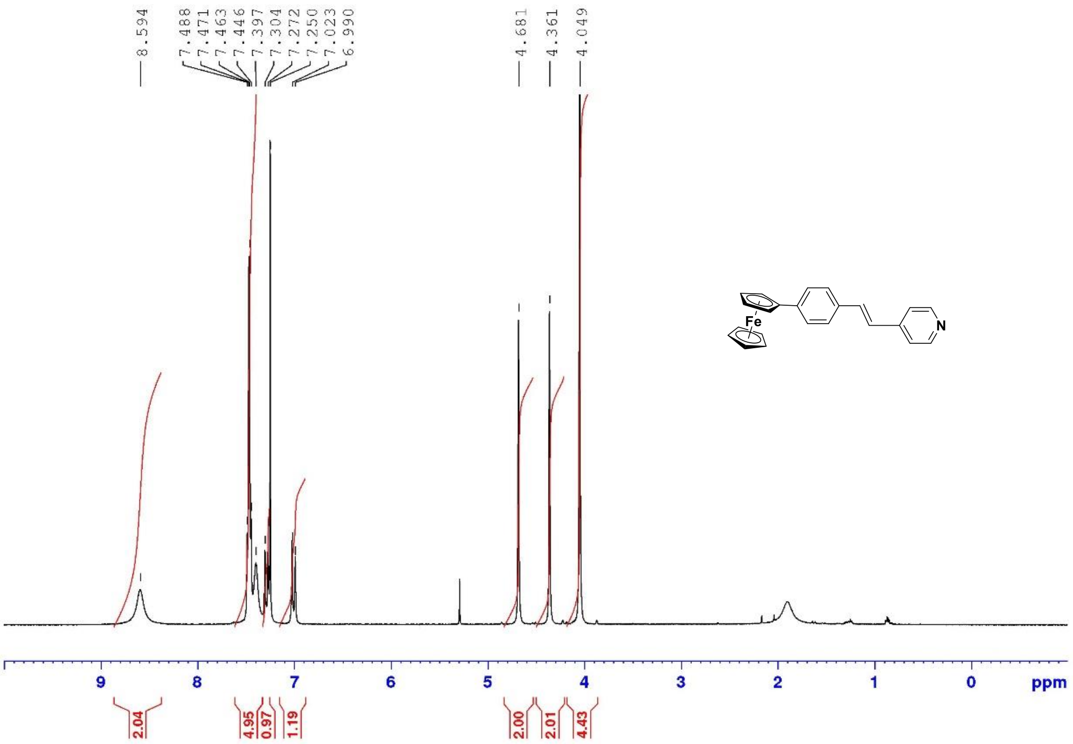

Figure S48. ^{1}H NMR spectrum of chromophore **5a** (500 MHz, $CDCl_3$, 25 °C).

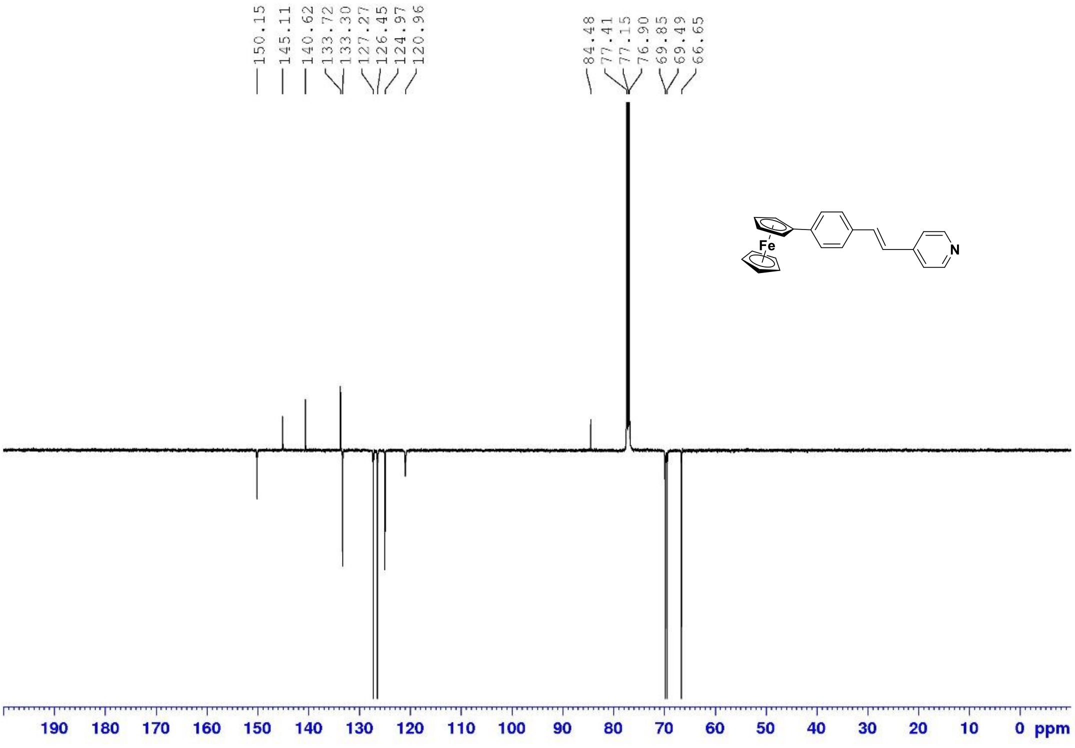

Figure S49. ^{13}C NMR APT spectrum of chromophore **5a** (125 MHz, $CDCl_3$, 25 °C).

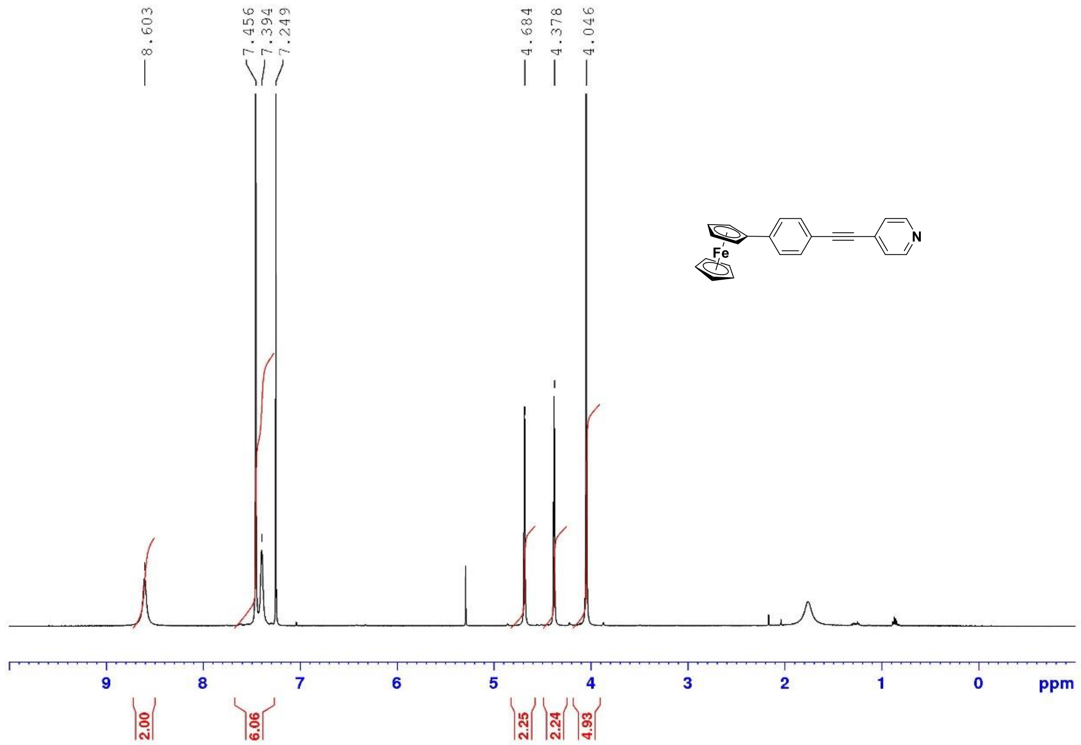

Figure S50. ^{1}H NMR spectrum of chromophore **6a** (500 MHz, $CDCl_3$, 25 °C).

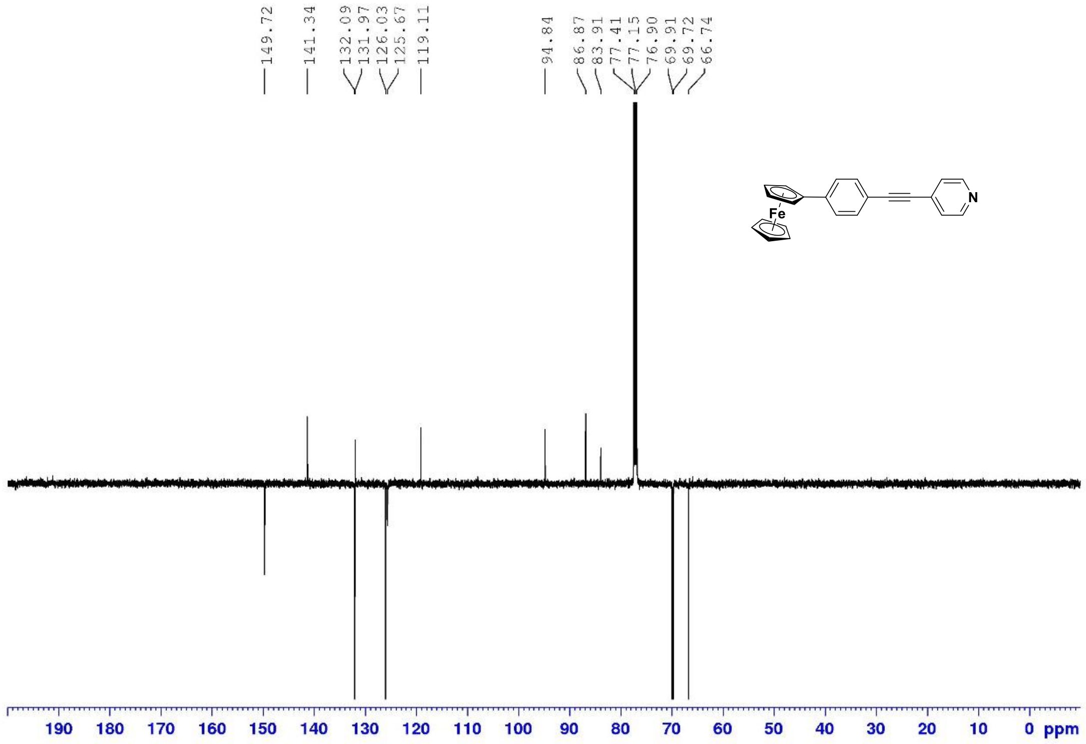

Figure S51. ^{13}C NMR APT spectrum of chromophore **6a** (125 MHz, $CDCl_3$, 25 °C).

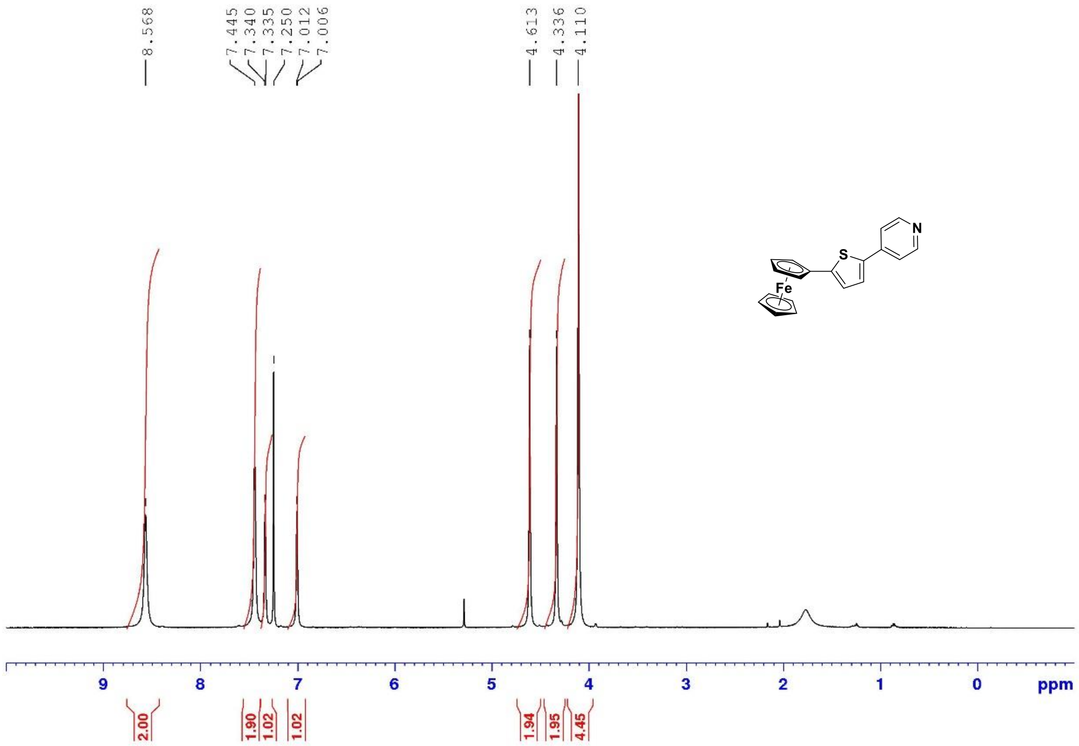

Figure S52. ^{1}H NMR spectrum of chromophore **7a** (500 MHz, $CDCl_3$, 25 °C).

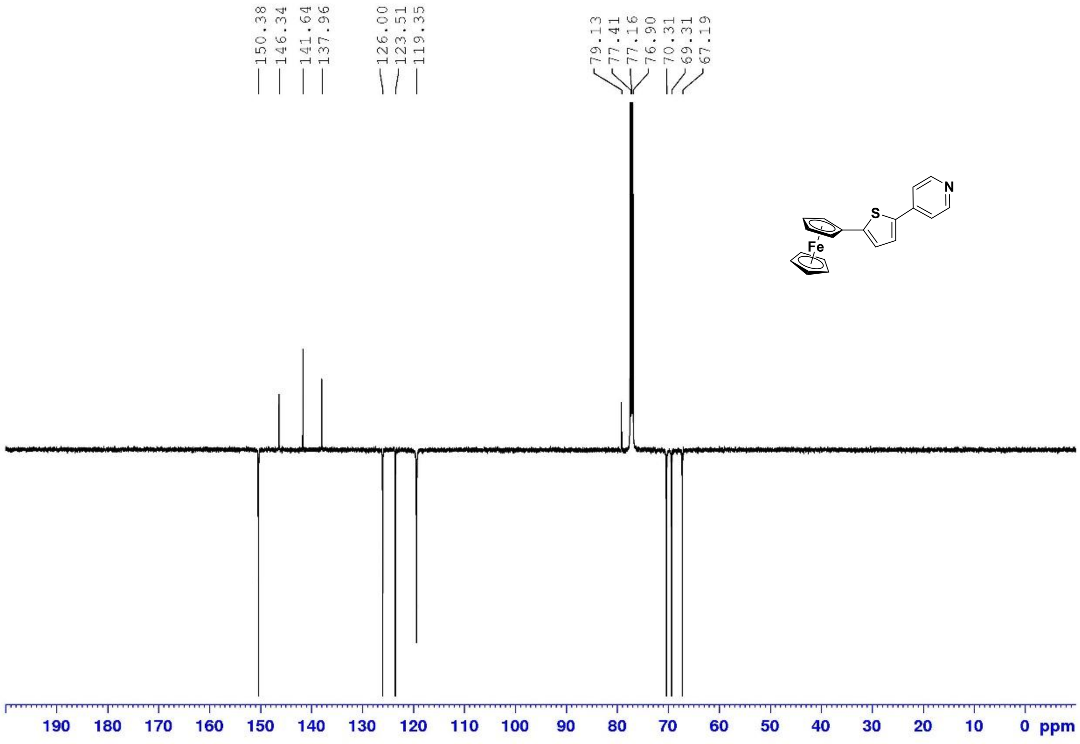

Figure S53. ^{13}C NMR APT spectrum of chromophore **7a** (125 MHz, $CDCl_3$, 25 °C).

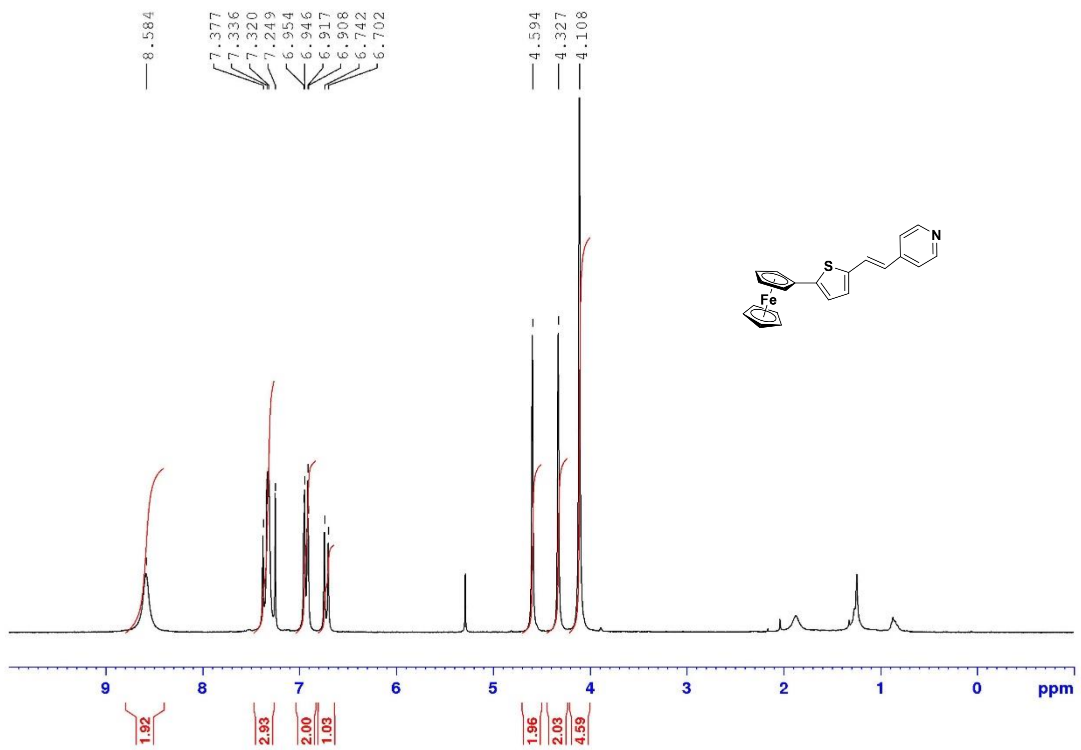

Figure S54. ^{1}H NMR spectrum of chromophore **8a** (400 MHz, $CDCl_3$, 25 °C).

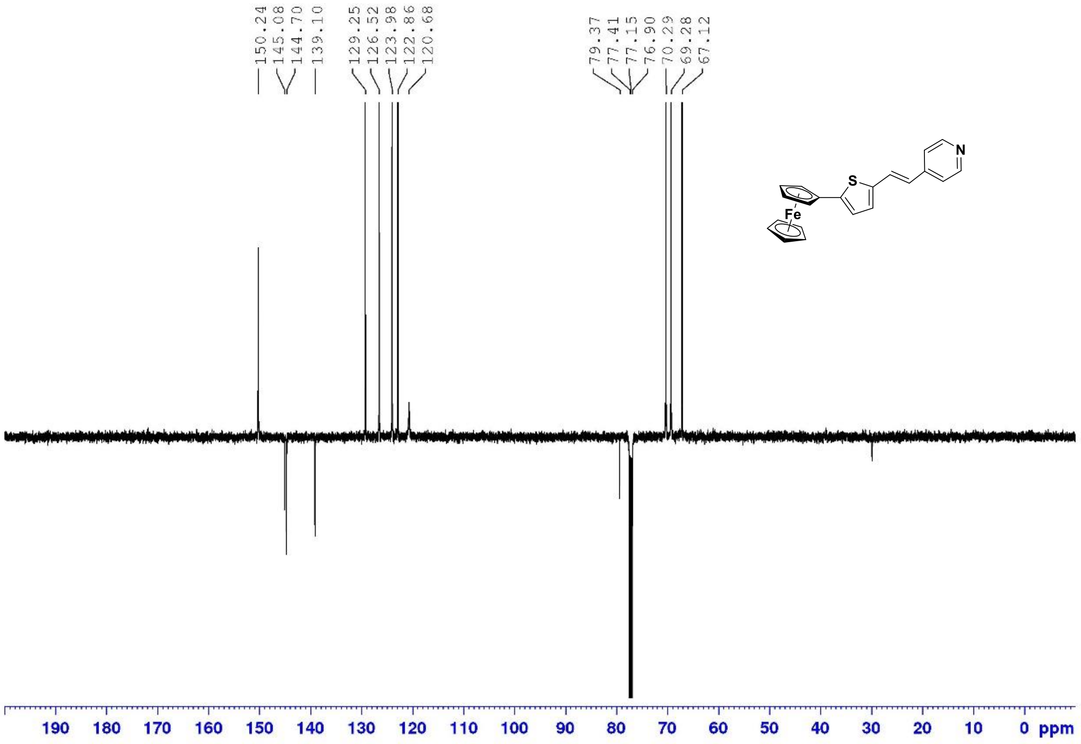

Figure S55. ^{13}C NMR APT spectrum of chromophore **8a** (125 MHz, $CDCl_3$, 25 °C).

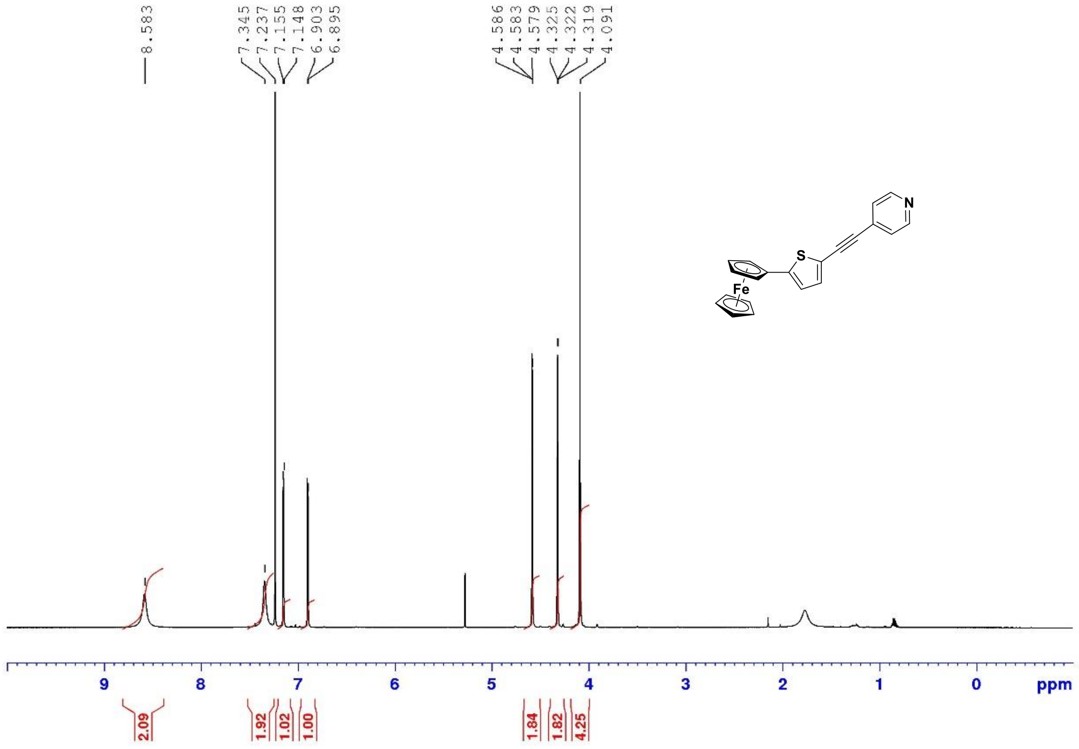

Figure S56. ^{1}H NMR spectrum of chromophore **9a** (500 MHz, $CDCl_3$, 25 °C).

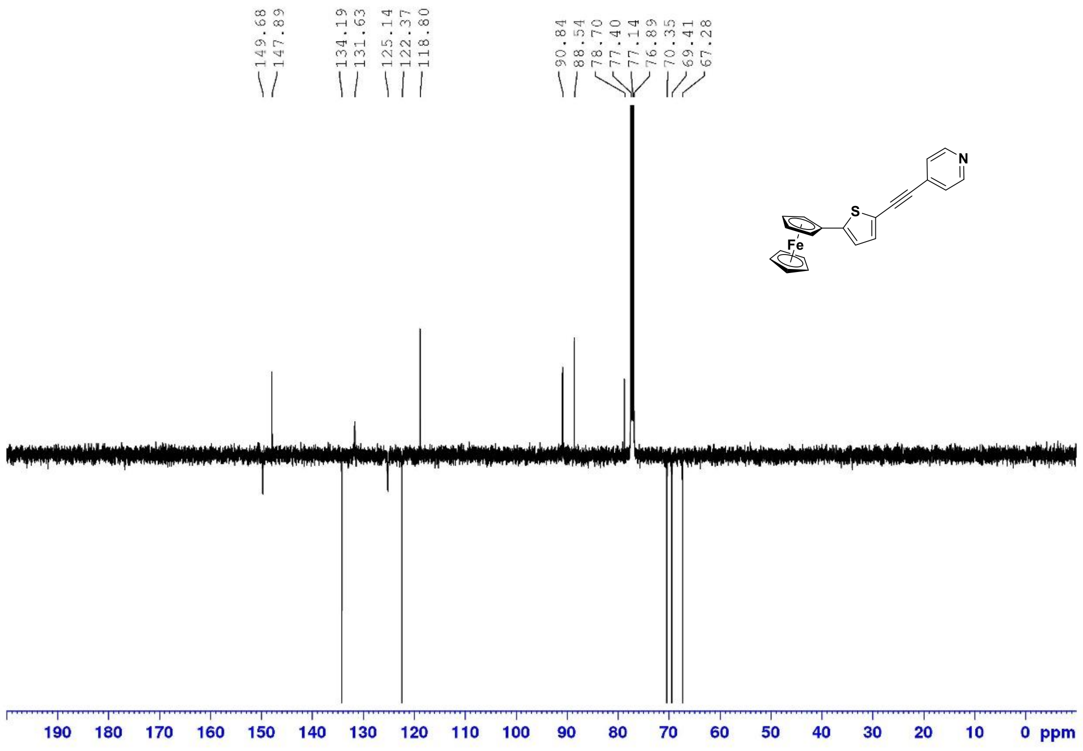

Figure S57. ^{13}C NMR APT spectrum of chromophore **9a** (125 MHz, $CDCl_3$, 25 °C).

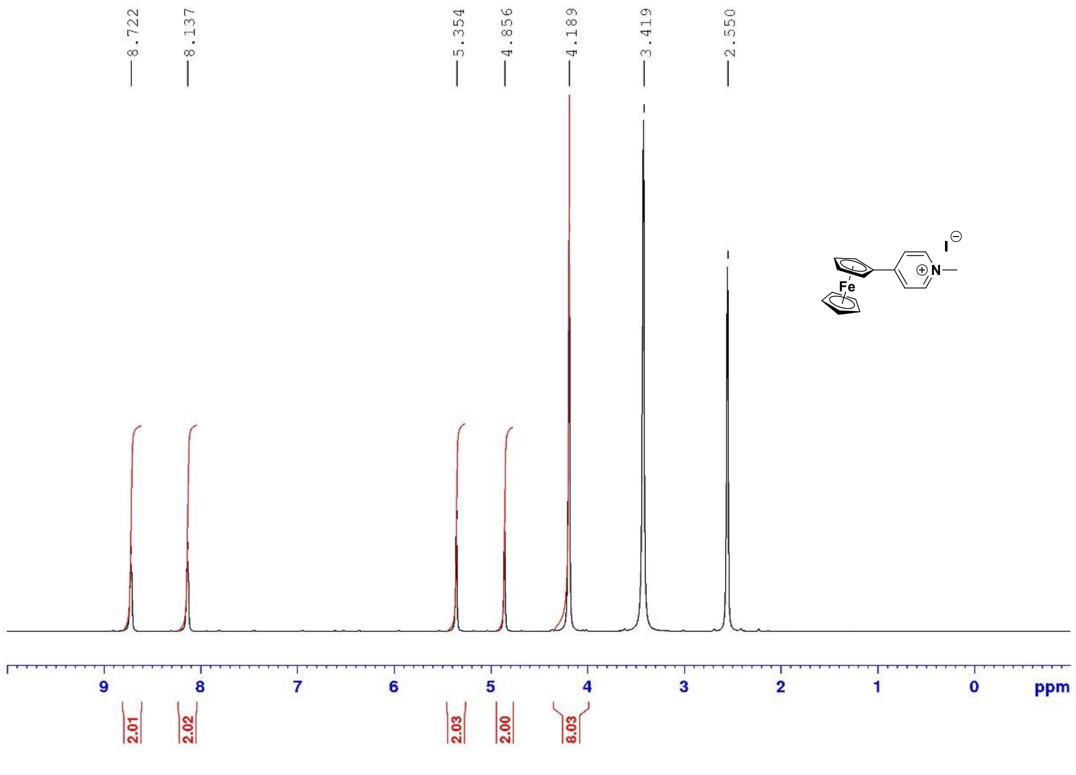

Figure S58. ^{1}H NMR spectrum of chromophore **1b** (500 MHz, DMSO-D$_6$, 25 °C).

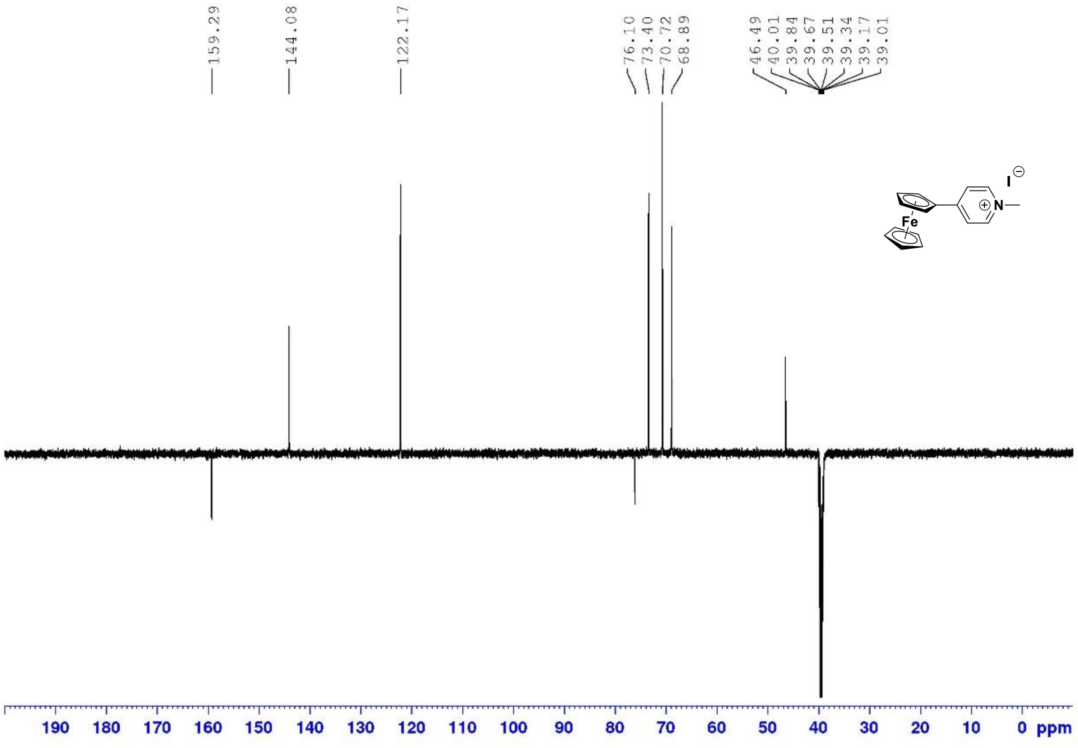

Figure S59. ^{13}C NMR APT spectrum of chromophore **1b** (125 MHz, DMSO-D$_6$, 25 °C).

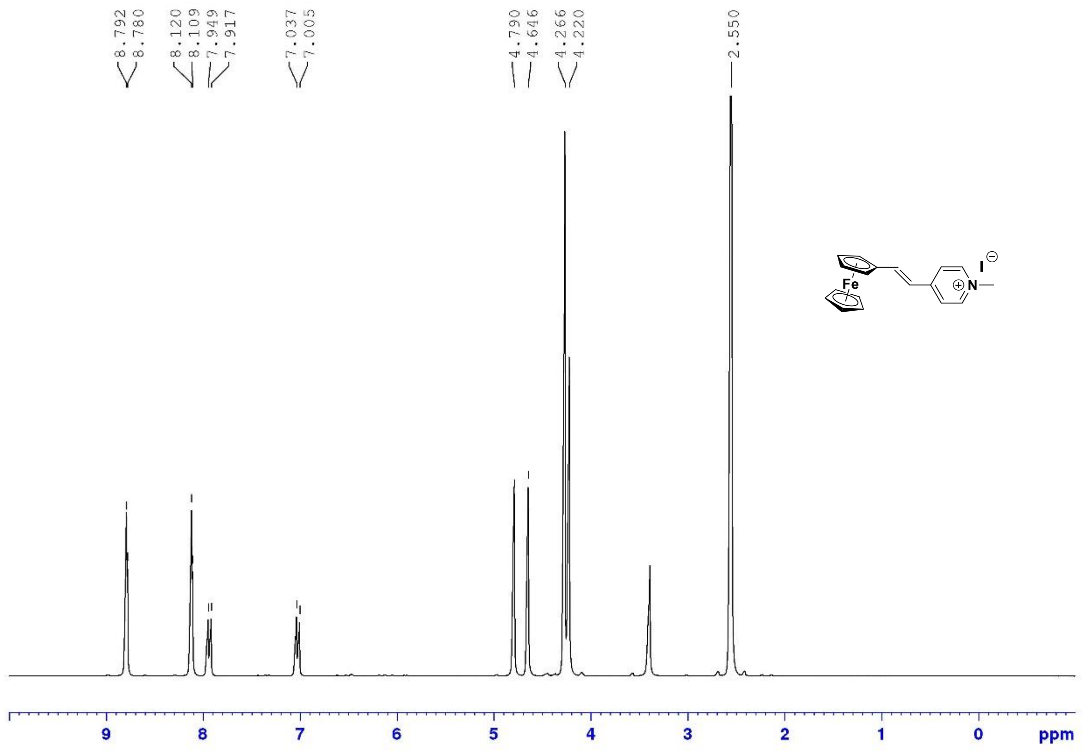

Figure S60. ^{1}H NMR spectrum of chromophore **2b** (500 MHz, DMSO-D_6, 25 °C).

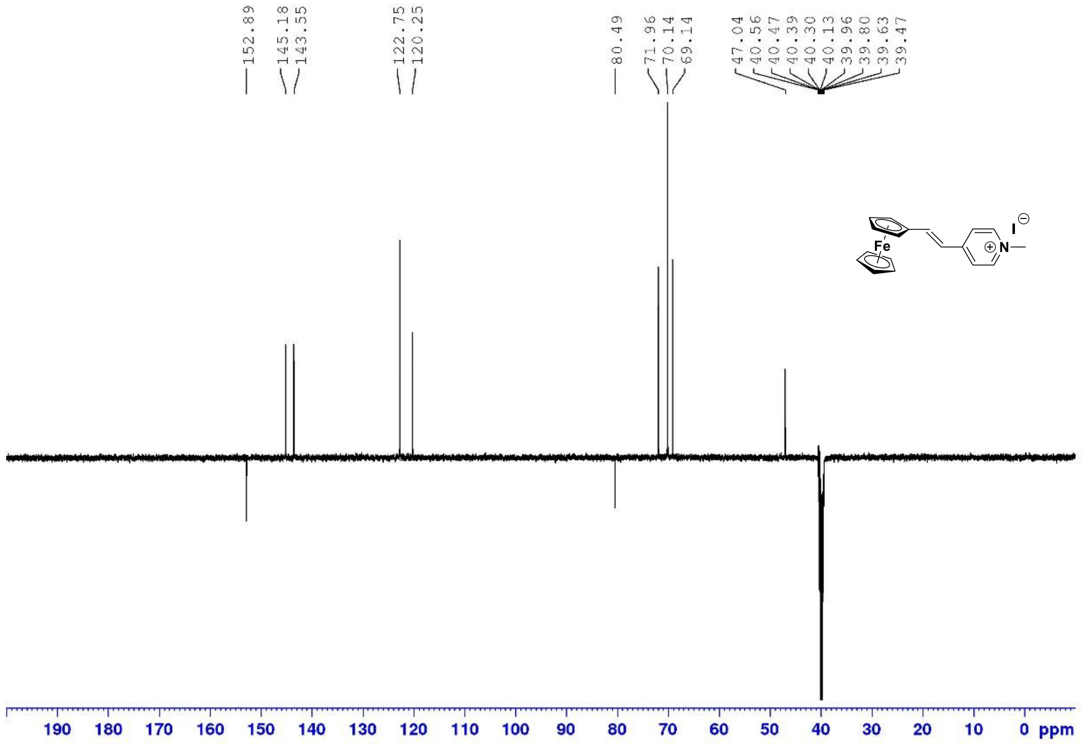

Figure S61. ^{13}C NMR APT spectrum of chromophore **2b** (125 MHz, DMSO-D_6, 25 °C).

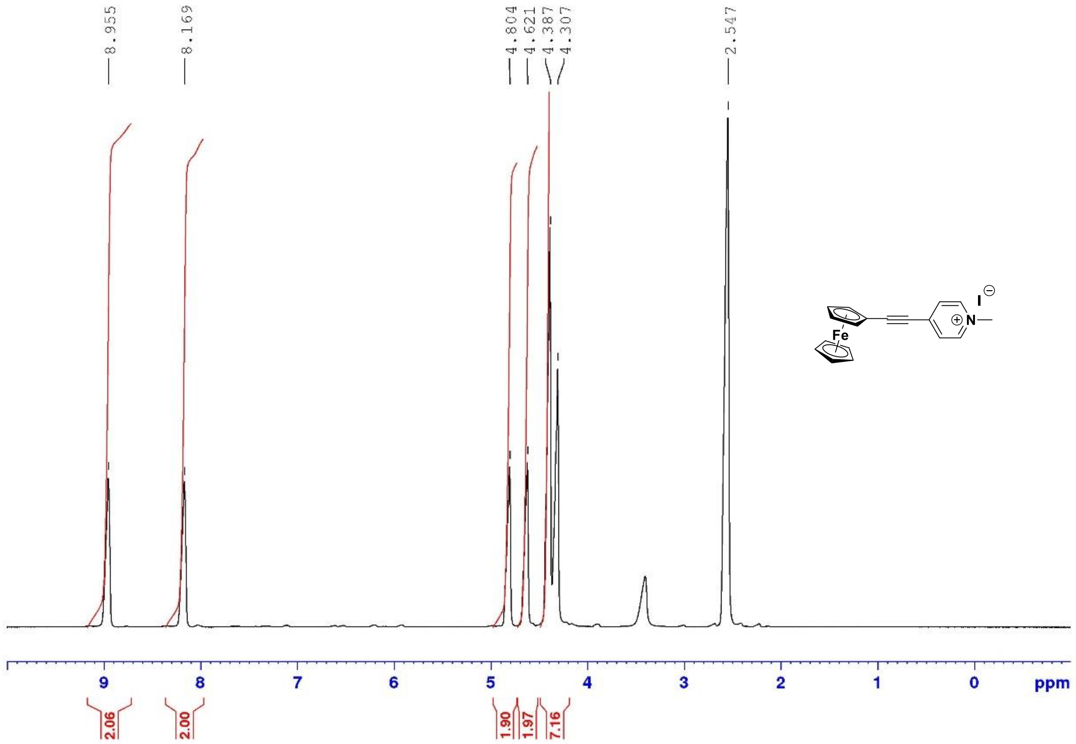

Figure S62. ^{1}H NMR spectrum of chromophore **3b** (500 MHz, DMSO-D$_6$, 25 °C).

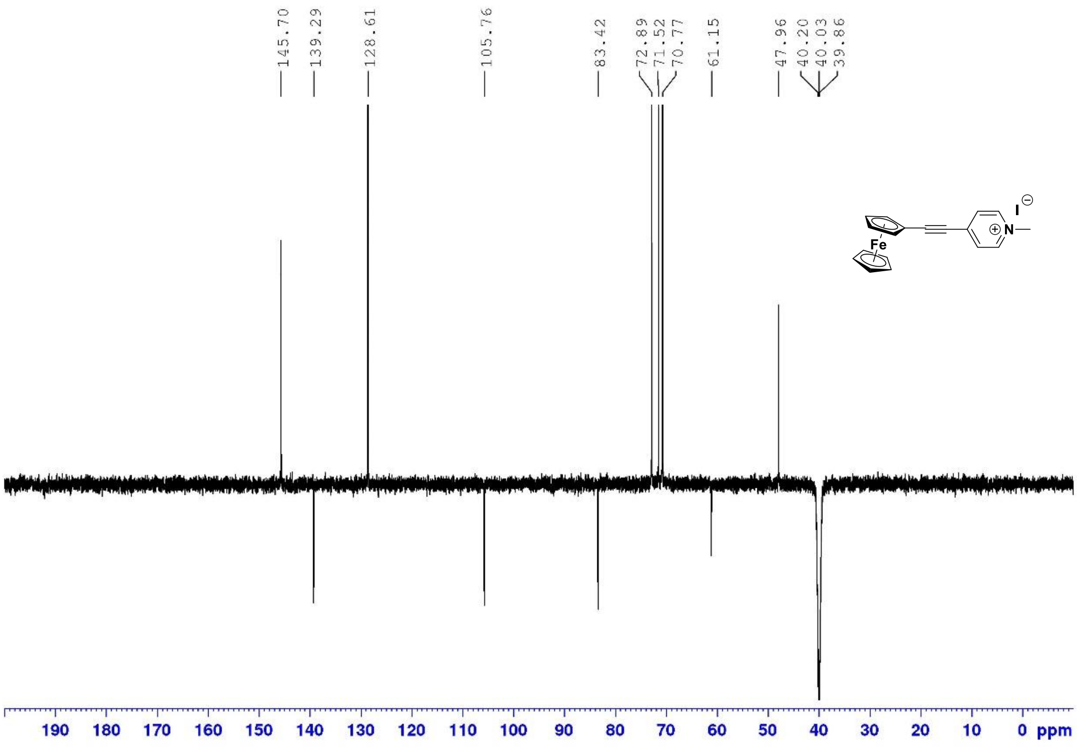

Figure S63. ^{13}C NMR APT spectrum of chromophore **3b** (125 MHz, DMSO-D$_6$, 25 °C).

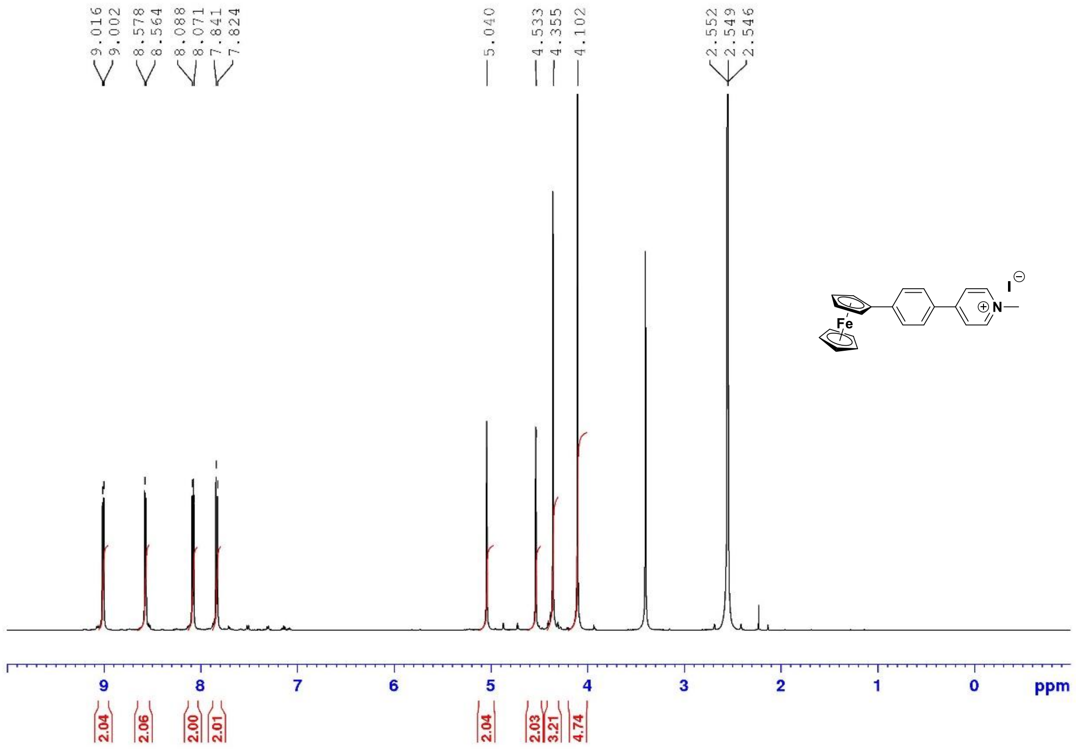

Figure S64. ^{1}H NMR spectrum of chromophore **4b** (500 MHz, DMSO-D_6, 25 °C).

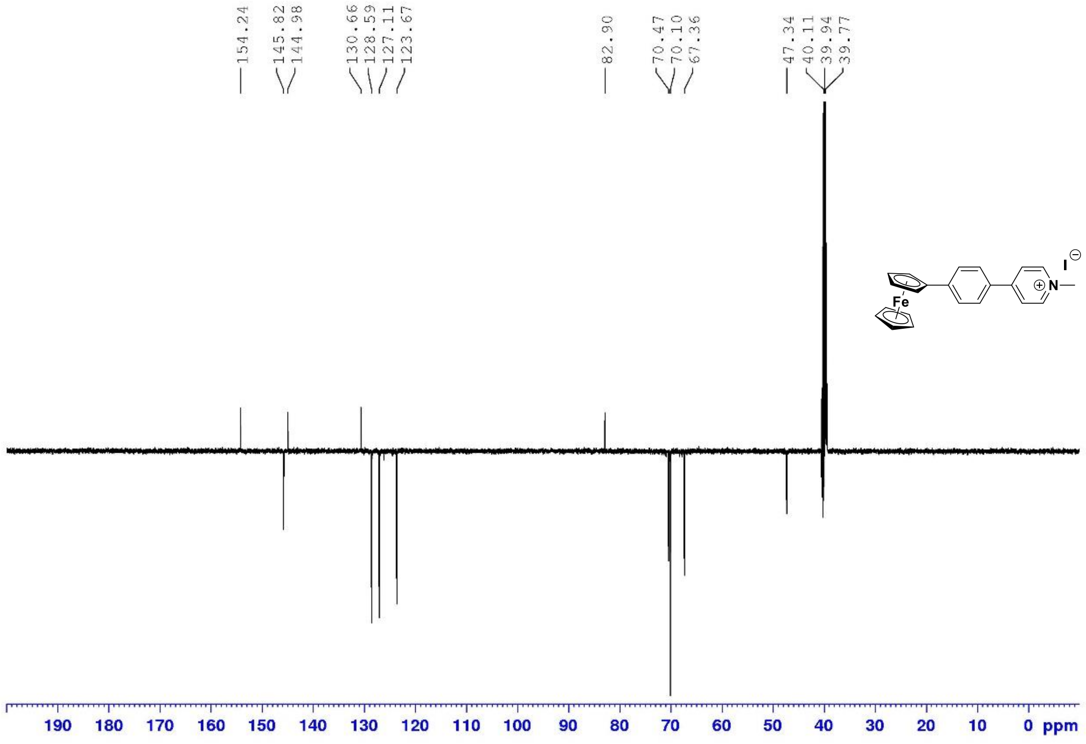

Figure S65. ^{13}C NMR APT spectrum of chromophore **4b** (125 MHz, DMSO-D_6, 25 °C).

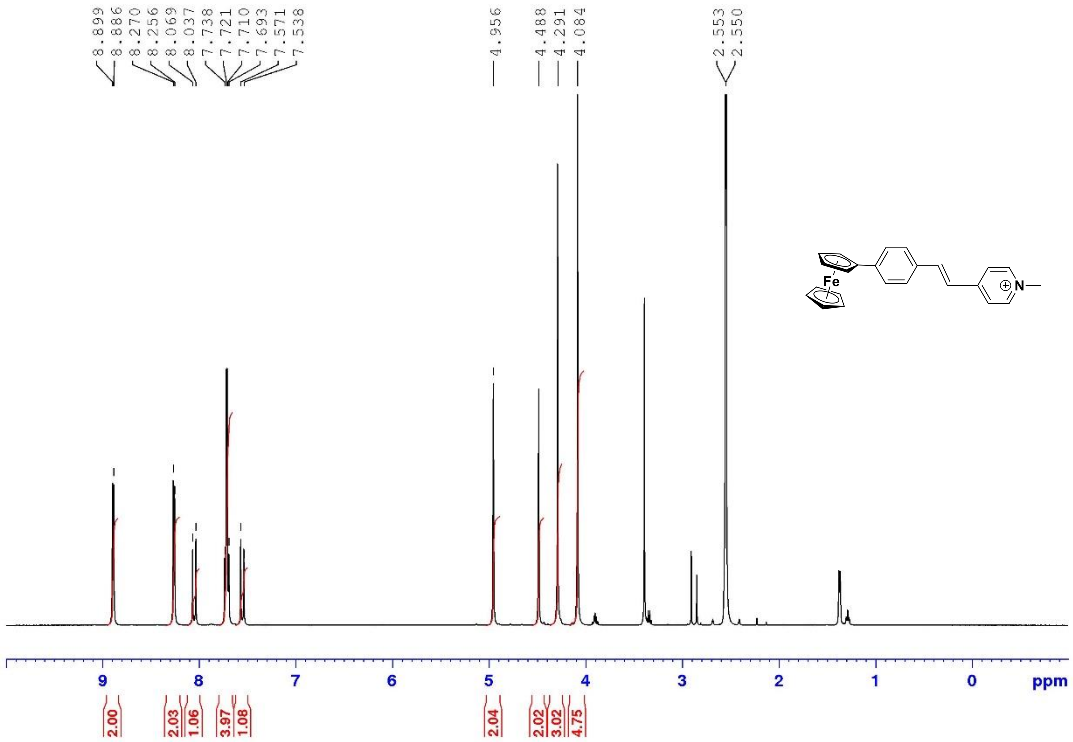

Figure S66. ^{1}H NMR spectrum of chromophore **5b** (500 MHz, DMSO-D_6, 25 °C).

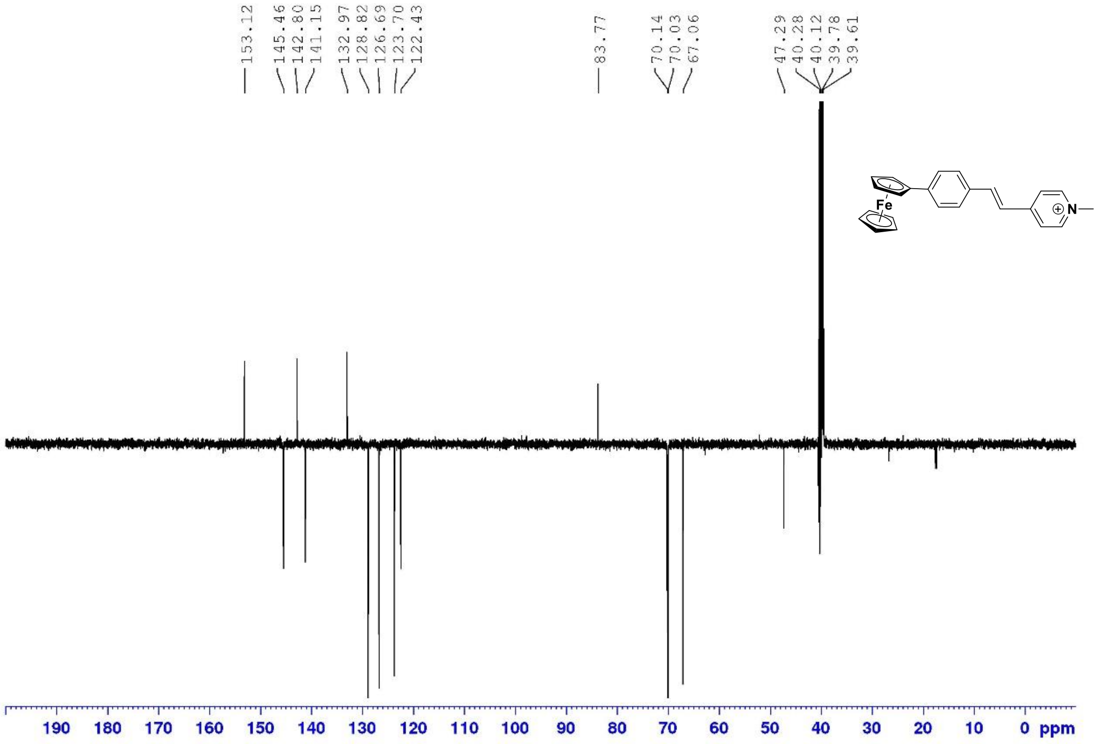

Figure S67. ^{13}C NMR APT spectrum of chromophore **5b** (125 MHz, DMSO-D_6, 25 °C).

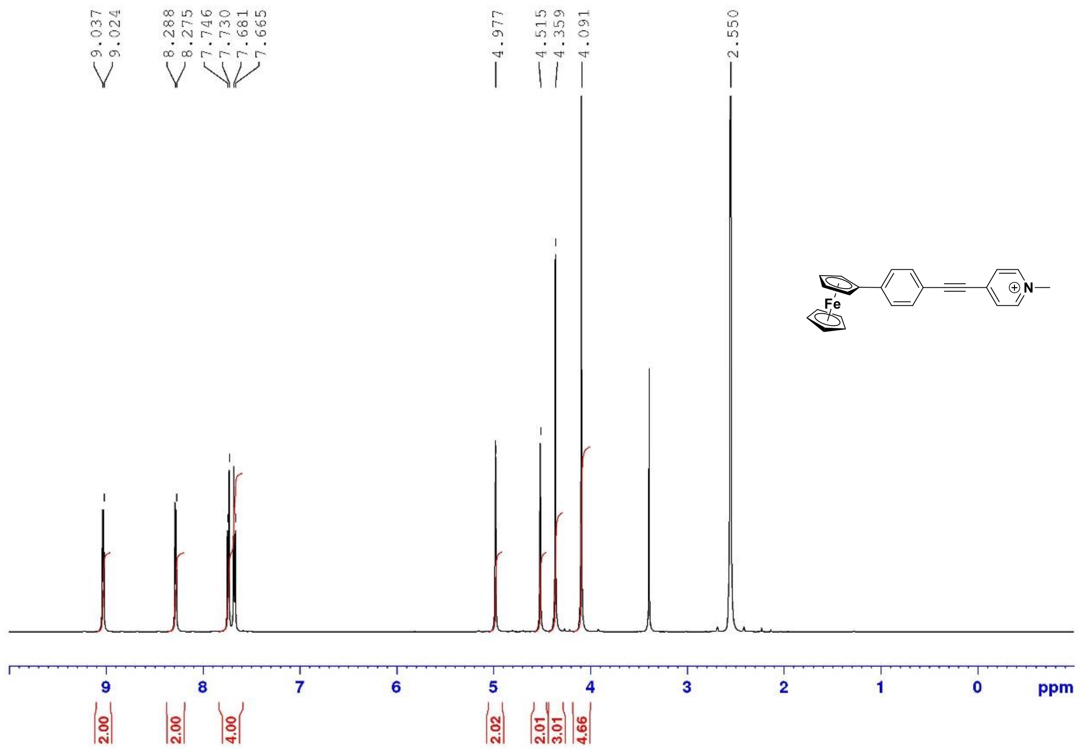

Figure S68. ^{1}H NMR spectrum of chromophore **6b** (500 MHz, DMSO-D$_6$, 25 °C).

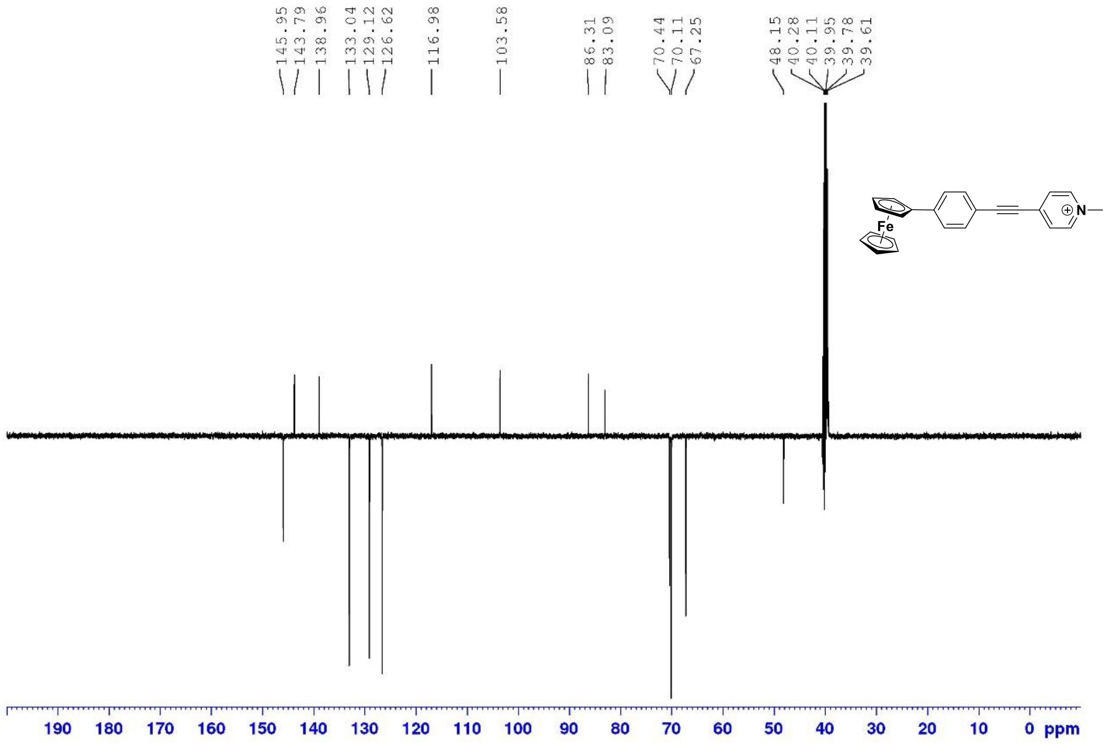

Figure S69. ^{13}C NMR APT spectrum of chromophore **6b** (125 MHz, DMSO-D$_6$, 25 °C).

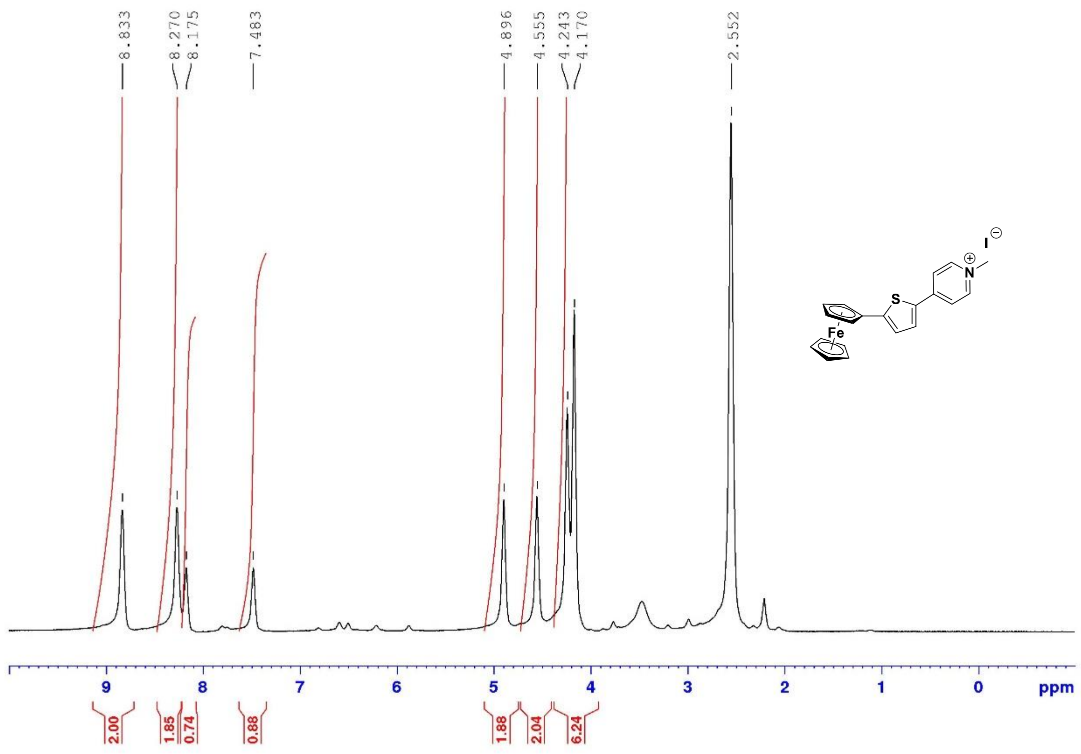

Figure S70. ^{1}H NMR spectrum of chromophore **7b** (500 MHz, DMSO-D$_6$, 25 °C).

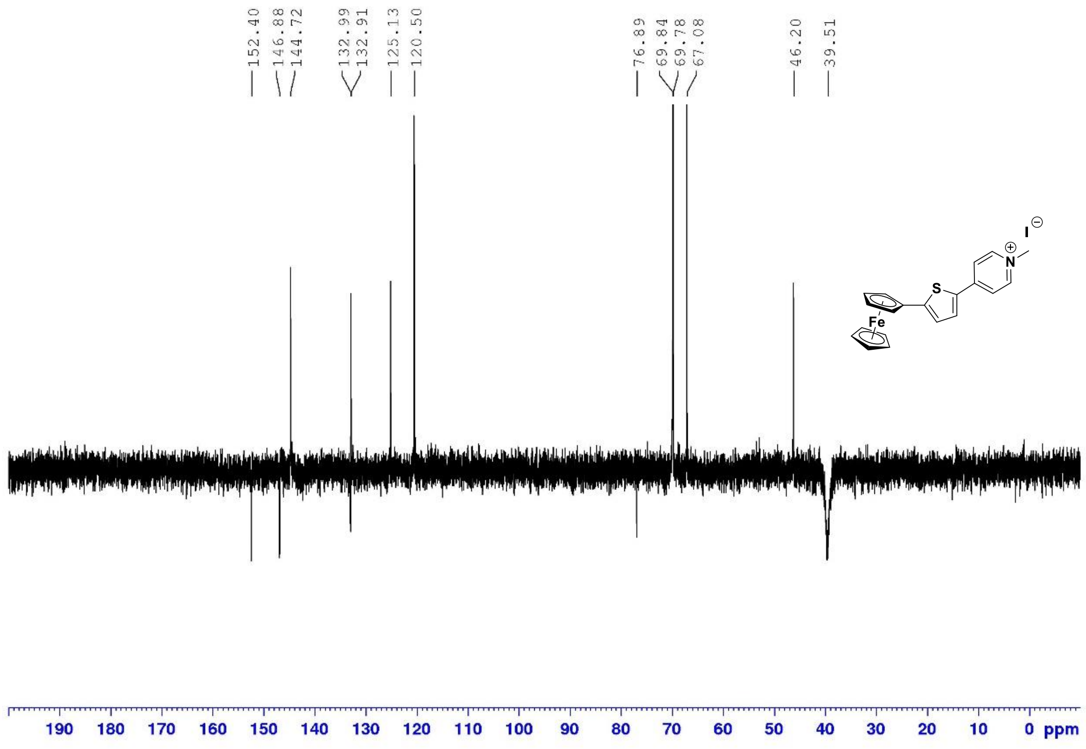

Figure S71. ^{13}C NMR APT spectrum of chromophore **7b** (125 MHz, DMSO-D$_6$, 25 °C).

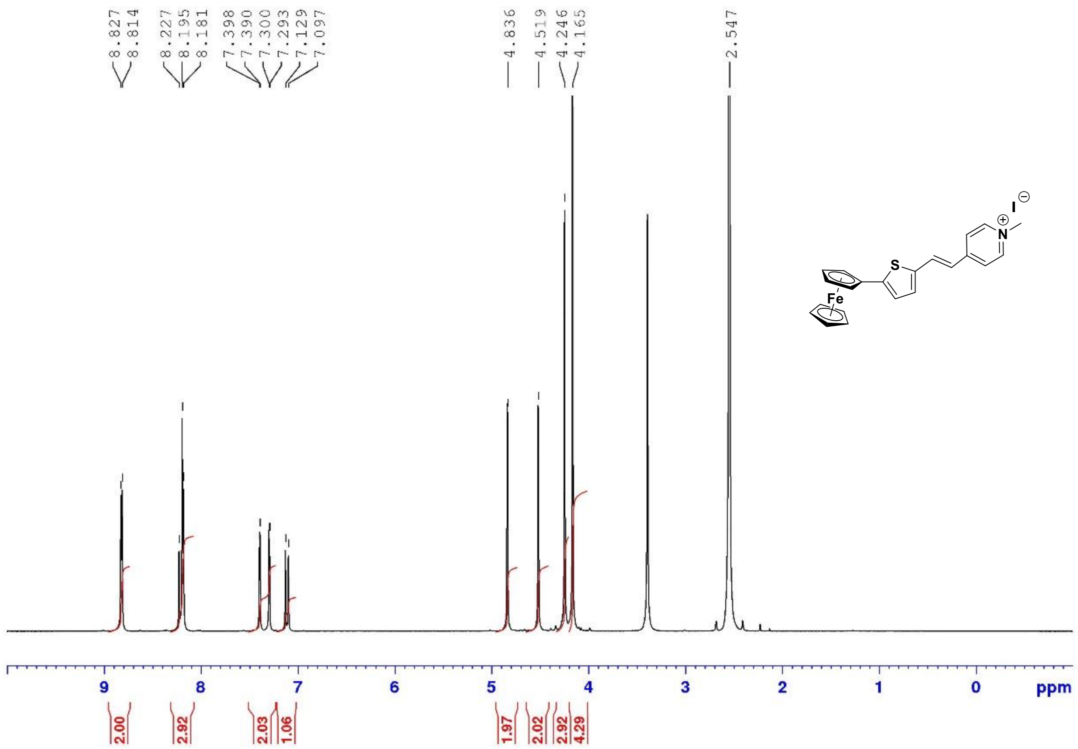

Figure S72. ^{1}H NMR spectrum of chromophore **8b** (500 MHz, DMSO-D_6, 25 °C).

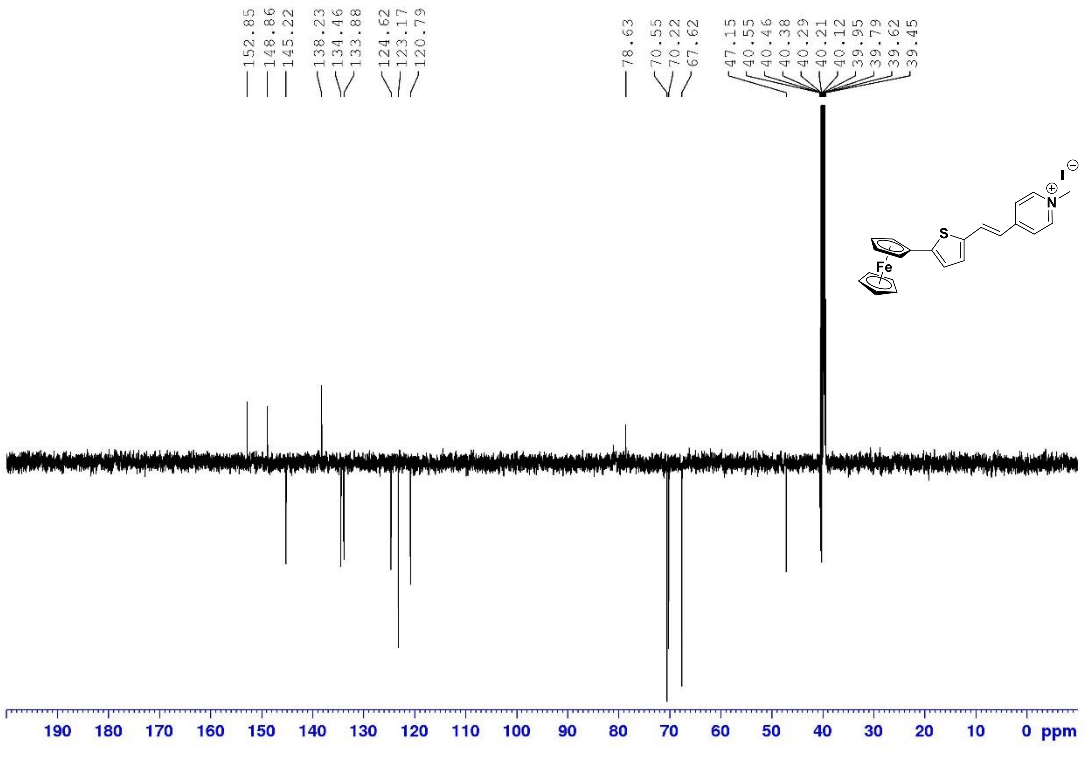

Figure S73. ^{13}C NMR APT spectrum of chromophore **8b** (125 MHz, DMSO-D_6, 25 °C).

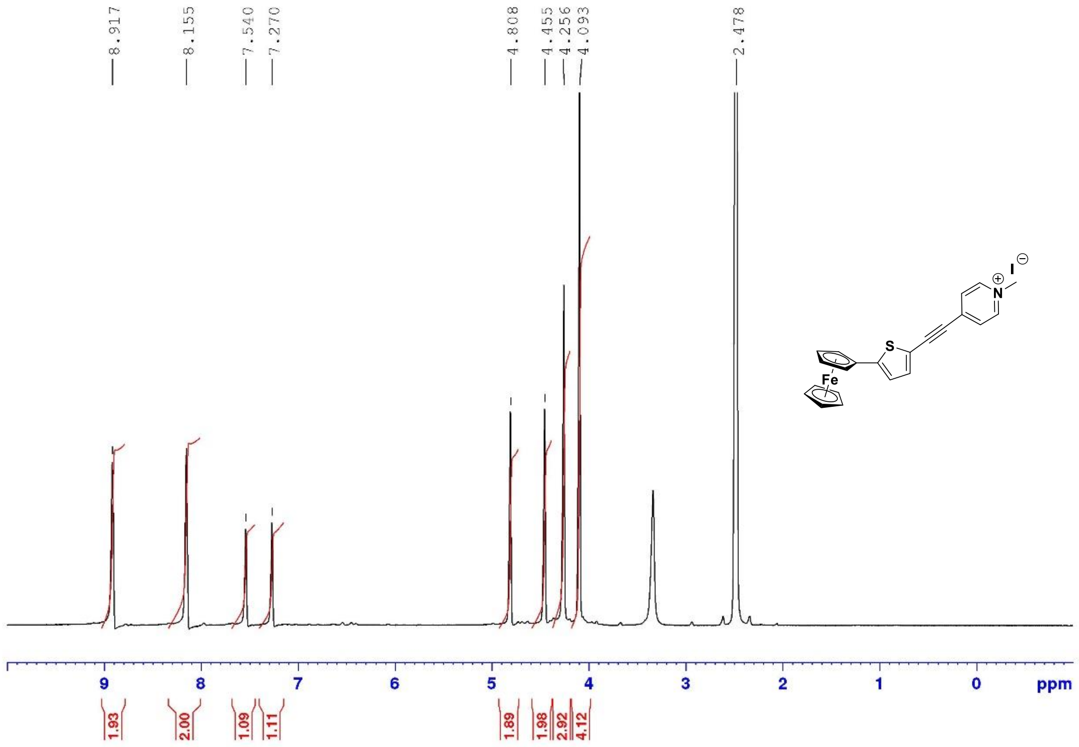

Figure S74. ^{1}H NMR spectrum of chromophore **9b** (500 MHz, DMSO-D$_6$, 25 °C).

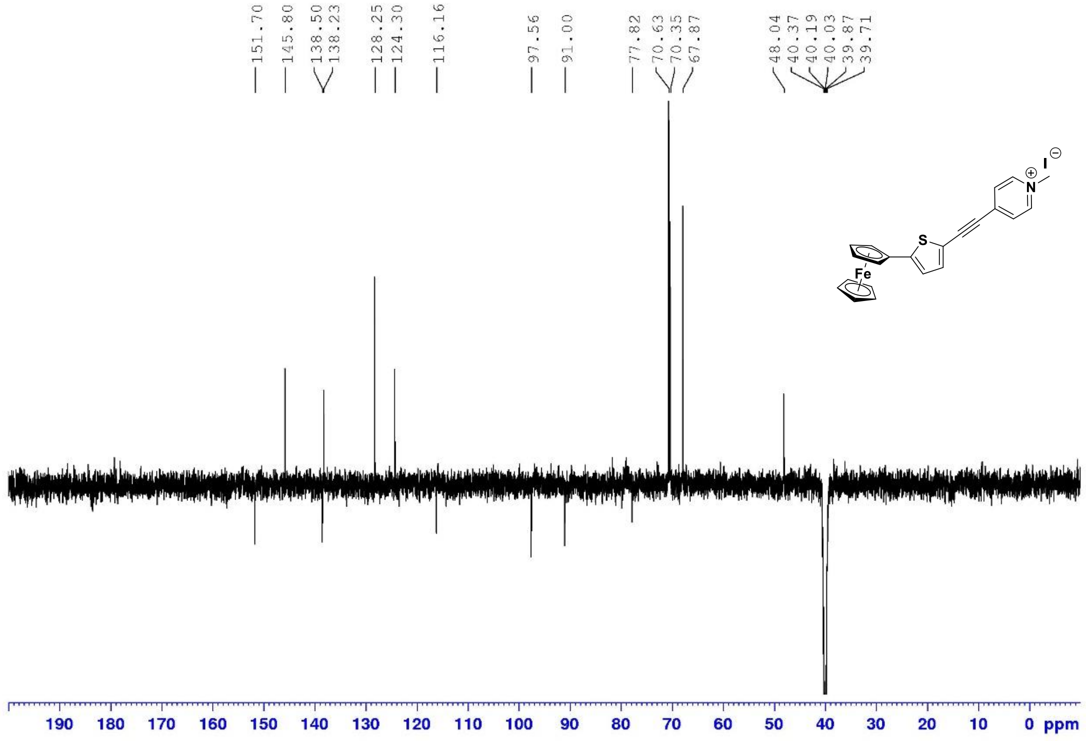

Figure S75. ^{13}C NMR APT spectrum of chromophore **9b** (125 MHz, DMSO-D$_6$, 25 °C).

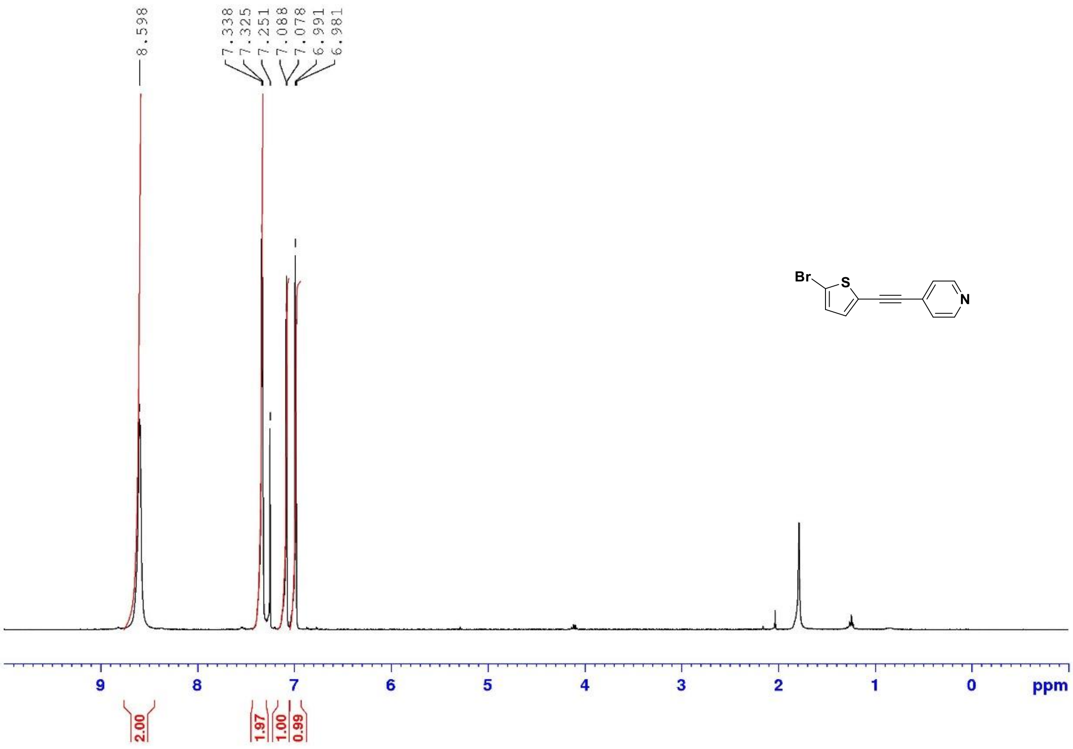

Figure S76. ^{1}H NMR spectrum of intermediate **17** (400 MHz, $CDCl_3$, 25 °C).

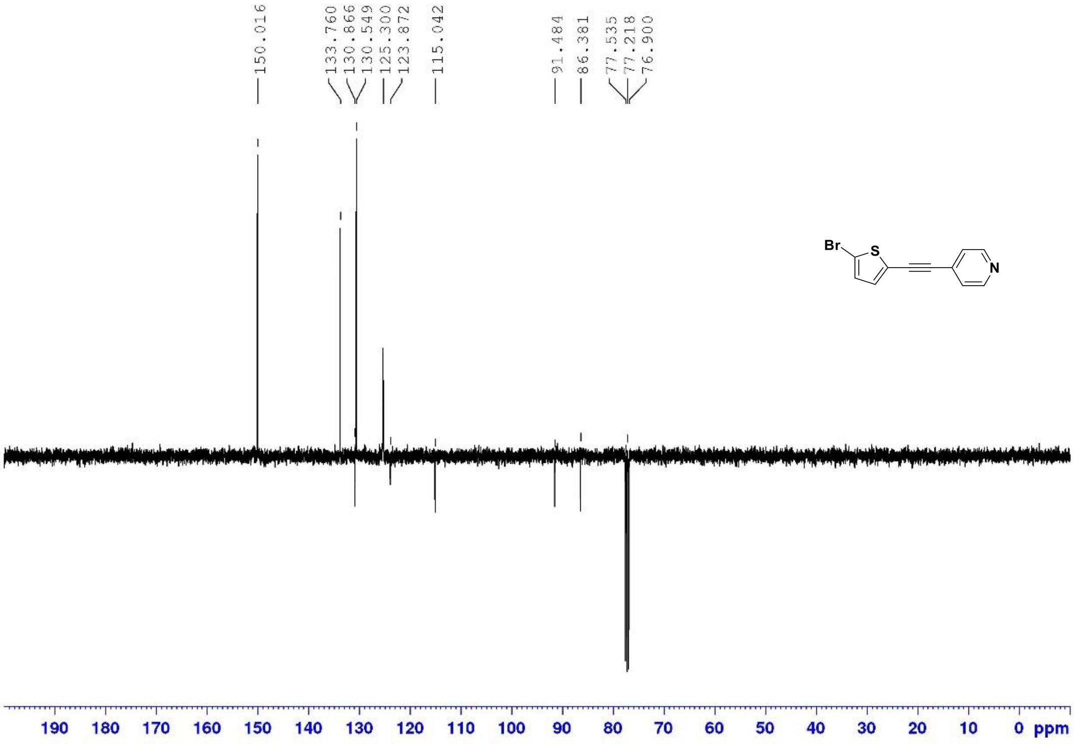

Figure S77. ^{13}C NMR APT spectrum of intermediate **17** (100 MHz, $CDCl_3$, 25 °C).

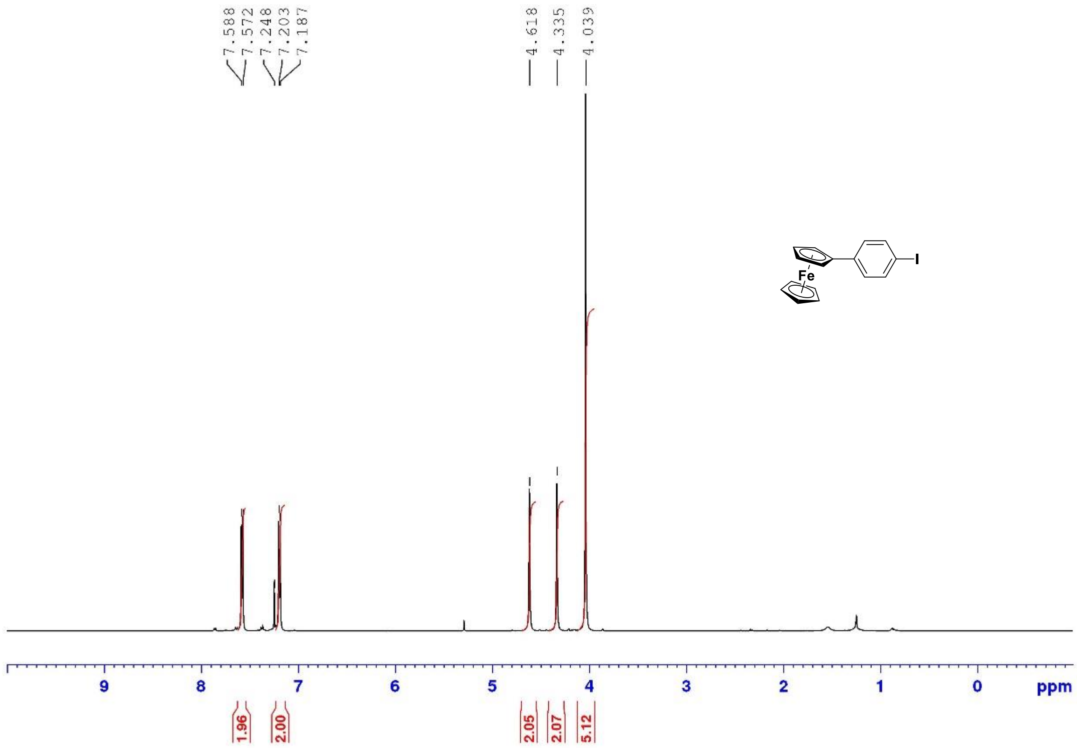

Figure S78. ^{1}H NMR spectrum of intermediate **18** (500 MHz, $CDCl_3$, 25 °C).

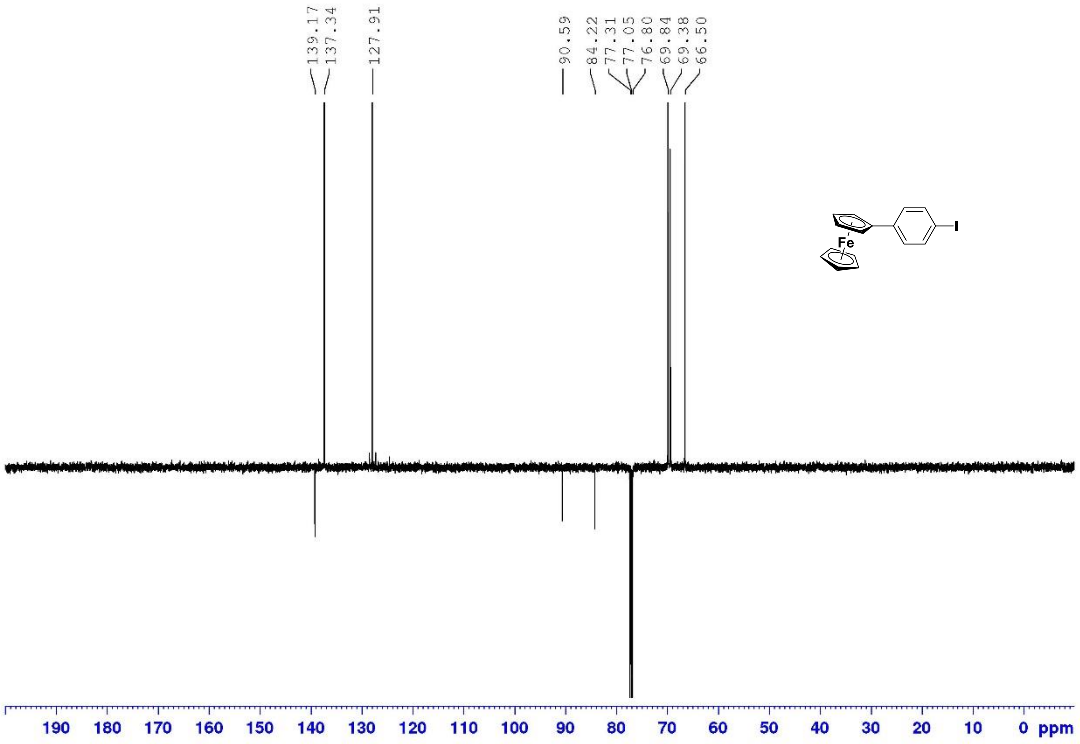

Figure S79. ^{13}C NMR APT spectrum of intermediate **18** (125 MHz, $CDCl_3$, 25 °C).
